# Recent advances in synthetic approaches for bioactive cinnamic acid derivatives

**DOI:** 10.3762/bjoc.21.85

**Published:** 2025-05-28

**Authors:** Betty A Kustiana, Galuh Widiyarti, Teni Ernawati

**Affiliations:** 1 Research Center for Pharmaceutical Ingredients and Traditional Medicine, National Research and Innovation Agency of Indonesia (BRIN), KST BJ Habibie, Kawasan Puspiptek, 452 Building, Tangerang Selatan 15314, Indonesia

**Keywords:** biologically active, cinnamic acid derivatives, environmentally sustainable, synthetic methodologies

## Abstract

Cinnamic acid derivatives represent a significant class of biologically active compounds exhibiting a broad spectrum of activities, such as antifungal, antidengue, antimetastatic, antimicrobial, antibacterial, and anticancer properties. Their preparation has attracted considerable attention due to their versatile applications across the pharmaceutical, food, and chemical sectors. This review elucidates the functional groups of cinnamic acid that are instrumental in the rational design of biologically active derivatives. A comprehensive representative of recent advancements in synthetic methodologies over the past five years is presented, particularly emphasizing the active scaffolds of bioactive cinnamic acid derivatives. The review provides a strategic overview of alternative synthetic routes and highlights the latest innovations, including more efficient, highly selective, and environmentally sustainable approaches. Given the widespread incorporation of the cinnamic acid framework in various therapeutic agents, this review delivers critical insights into a molecular design for hit-to-lead optimization, offering detailed synthetic strategies for diverse functional modifications. By critically examining these methodologies, the paper underscores their role in expanding the utility of cinnamic acid derivatives and addressing prevailing challenges.

## Review

### 1 Introduction

**Figure 1:**
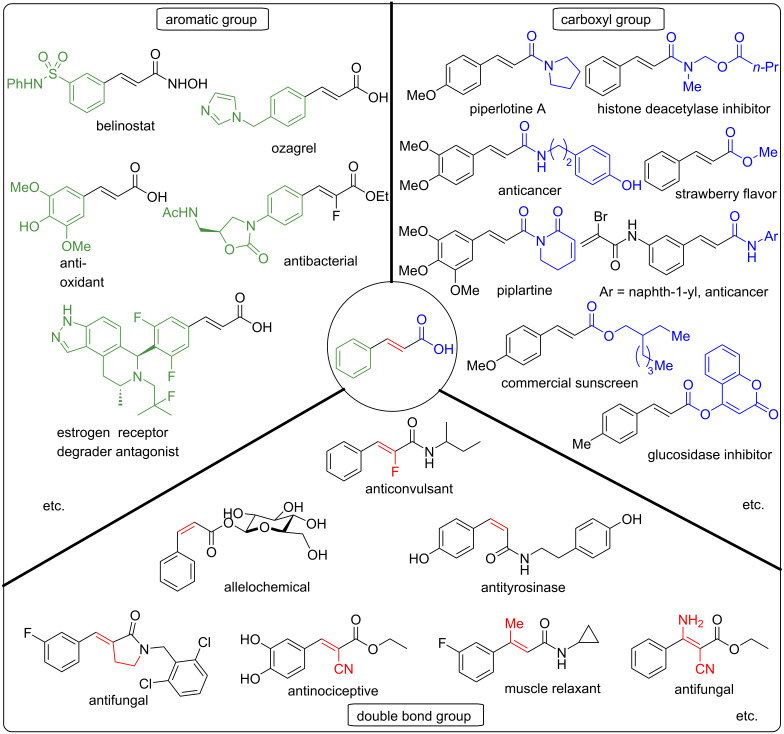
Biologically active cinnamic acid derivatives.

Cinnamic acid is a naturally occurring plant metabolite frequently found in honey, fruits, and vegetables [1]. Cinnamic acid is biosynthesized through a shikimate pathway, catalyzed by the phenylalanine ammonia-lyase (PAL) enzyme. Generally, cinnamic acid derivatives possess a wide range of bioactivities, such as flavor, fragrance, and therapeutic activities (Figure 1). Several reported activities of cinnamic acid include antibacterial and antifungal properties [1–4], antidengue [5], antimetastatic [6], neuroprotective synergy and angiogenesis effects [7], antileishmaniasis [8], anticancer [9], thromboxane (TXA2) synthetase inhibition [10], antinociceptive [11], histone deacetylase inhibition [12], α-glucosidase inhibition [13] , tyrosinase inhibition [14], allelochemical [15], anticonvulsant [16], antioxidant [17], antiplatelet aggregation [18], anti-inflammatory [19], and UV absorption [20].

**Scheme 1:**
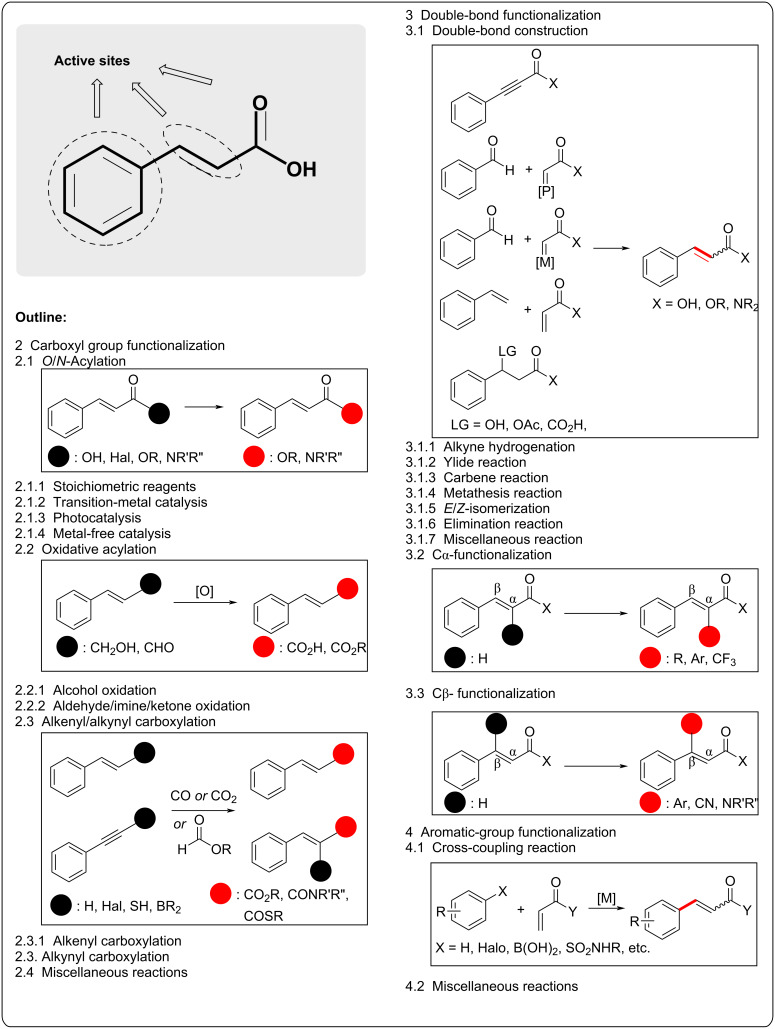
General synthetic strategies for cinnamic acid derivatizations.

Cinnamic acid possesses three distinct functional groups: carboxyl, alkenyl, and aromatic (Scheme 1). Therefore, highly selective reaction strategies are immensely desired for cinnamic acid derivatizations, preventing other functional groups from interfering. This review is partitioned into specific methodologies based on the functional groups of cinnamic acid reported in the last five years: modifying the carboxyl group can involve several pathways, such as *O*/*N*-acylation, oxidative acylation, alkenyl/alkynyl carboxylation, and other reactions. Altering the double bond can be approached through double-bond construction, alkyne hydrogenation, ylide and carbene reaction, metathesis, *E*/*Z* isomerization, and other methods, including Cα and Cβ functionalizations. Preparing various functional group-tethered aromatic groups can be achieved by directly installing an aromatic group via cross-coupling reactions and other reaction types.

Recent advancements in synthetic chemistry and molecular engineering have created significant opportunities to modify the structural framework of cinnamic acid, resulting in novel derivatives with improved therapeutic potential and enhanced biological efficacy, thus triggering their broader applications in the pharmaceutical industry. In addition, innovative synthetic methodologies, including environmentally sustainable approaches [21–23], advanced catalytic systems [24–26], photocatalysis [27–29], and cutting-edge technologies, such as flow chemistry [30–31], have contributed significantly to the efficient and cost-effective production of cinnamic acid derivatives.

This review focuses on diverse, one-step strategies to access cinnamic acid derivatives with more efficient, highly selective, and sustainable approaches. By examining recent advancements in the design and synthesis of these derivatives, this study aims to offer direct synthetic guidance and important insights into the rational design of novel cinnamate molecules with promising potential as future drug candidates. The reaction mechanisms will be discussed briefly.

### 2 Carboxyl group functionalization

#### 2.1 *O*/*N*-acylations

**Scheme 2:**
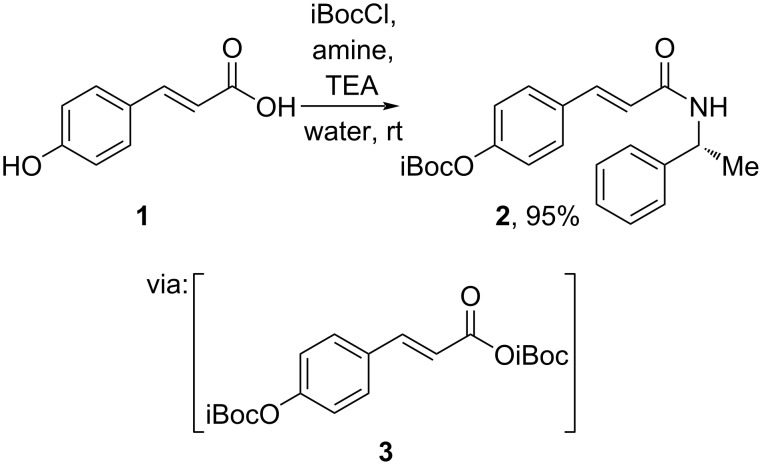
Cinnamic acid coupling via isobutyl anhydride formation.

**2.1.1 Stoichiometric reagents:** Anhydride formation is one reliable method to activate the carboxylic group of cinnamic acid. For instance, in 2020, Longobardo and DellaGreca utilized isobutyl chloroformate in water to construct an *O*-protected amide derivative **2** of hydroxycinnamic acid **1** with an excellent yield (Scheme 2) [32]. The formed anhydride **3** was smoothly converted to the amide at room temperature. This method provides a green approach by allowing cinnamic acid derivatization in water as a benign solvent.

**Scheme 3:**
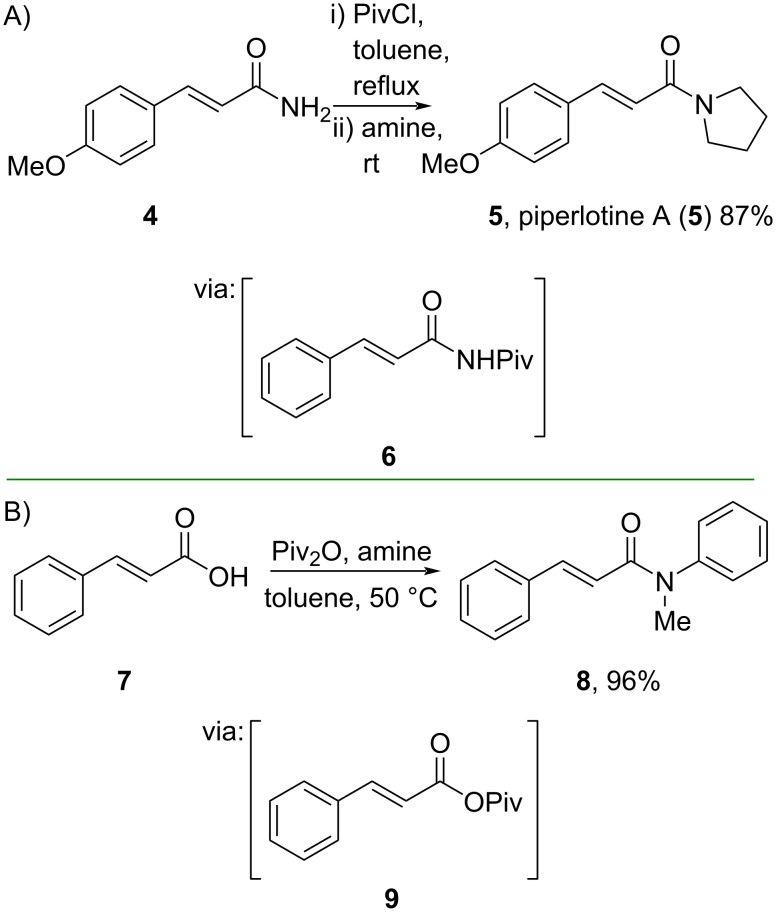
Amidation reaction via *O*/*N*-pivaloyl activation.

Similarly, Rajendran and Rajan (2023) reported a one-pot transamidation of cinnamamide **4** by utilizing pivaloyl chloride via the *N*-pivaloyl-activated amide **6** to give piperlotine A (**5**), the secondary metabolite of black pepper (*Piper nigrum*) reported to show antibacterial and bioinsecticidal activities, in good yield (Scheme 3A) [33–34]. In the akin process, Xu and co-workers (2023) reported a carboxyl group activation of cinnamic acid (**7**) by applying pivalic anhydride in a single step to afford the corresponding amide **8** in excellent yield (Scheme 3B) [35]. Moreover, pivalic anhydride is easier to handle than its chloride counterpart.

**Scheme 4:**
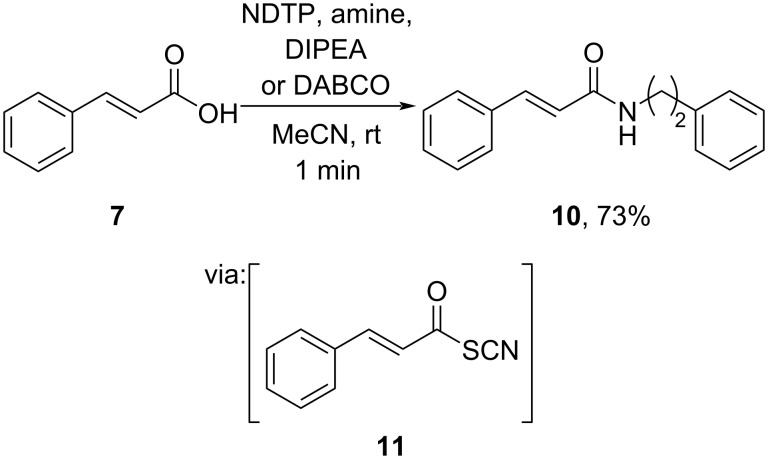
Cinnamic acid amidation using TCCA/PPh_3_ reagent.

Wang and co-workers (2022) utilized 5-nitro-4,6-dithiocyanatopyrimidine (NDTP) as the coupling reagent for cinnamic acid amidation with swift reaction time (Scheme 4) [22]. The reaction of cinnamic acid (**7**) with NDTP resulted in the active acyl thioester **11** followed by a reaction with an amine to give the corresponding amide **10**. The byproduct of the coupling reagent could be recycled by adding POCl_3_ and KSCN, thus promoting the reagent’s sustainability by reducing waste.

**Scheme 5:**
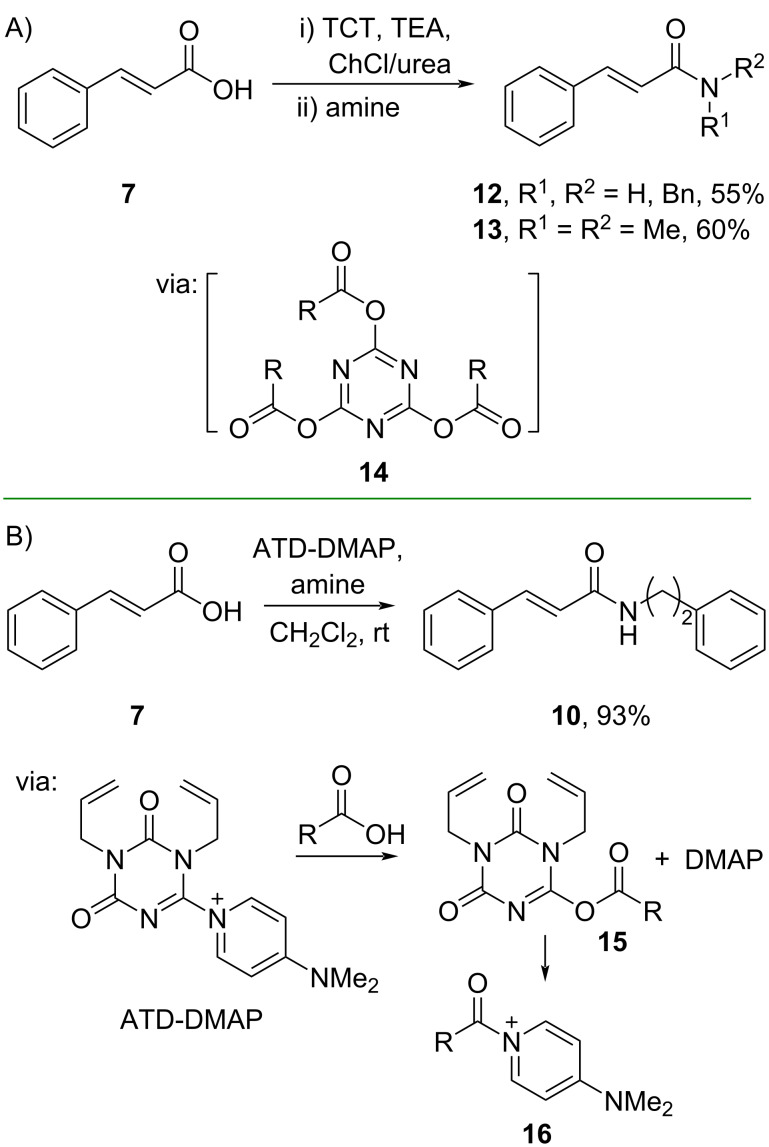
Cinnamic acid amidation using triazine-based reagents.

Carboxyl group activation can also be achieved by using triazine-based reagents. For example, Saberi and Salimiyan (2019) applied 2,4,6-trichloro-1,3,5-triazine (TCT) to perform amidation of cinnamic acid (**7**) in a deep eutectic solvent of choline chloride/urea (ChCl/urea) to give amides **12** and **13** in moderate yields via triacylated triazine **14** as the active ester (Scheme 5A) [36]. The TCT reagent and ChCl/urea solvent are known for their non-toxicity and low cost, promoting their wide applications in organic reactions. Similarly, Kunishima and co-workers (2021) utilized (*N*,*N*’-dialkyl)triazinedione-4-(dimethylamino)pyridine (ATD-DMAP) for the amidation of cinnamic acid (**7**) to generate the corresponding amide **10** in excellent yield (Scheme 5B) [37]. Mechanistically, the carboxyl group attacks the electrophilic triazinedione, releasing DMAP to give ester **15** which reacts with DMAP to afford the active *N*-acylpyridinium species **16**.

**Scheme 6:**
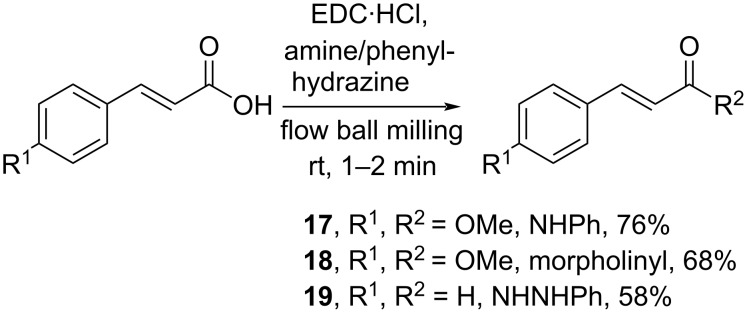
Cinnamic acid amidation using continuous flow mechanochemistry.

In addition, *N*-(3-dimethylaminopropyl)-*N*’-ethylcarbodiimide hydrochloride (EDC·HCl), a common coupling reagent, has been applied for a continuous flow mechanochemistry synthesis of cinnamic acid derivatives. Herein, Kulkarni and Atapalkar (2023) converted cinnamic acids into the corresponding amides **17** and **18** and hydrazide **19** in moderate yields (Scheme 6) [30]. Impressively, the reaction capacity could be increased to produce 100 g of the amide products with 90% yields.

**Scheme 7:**
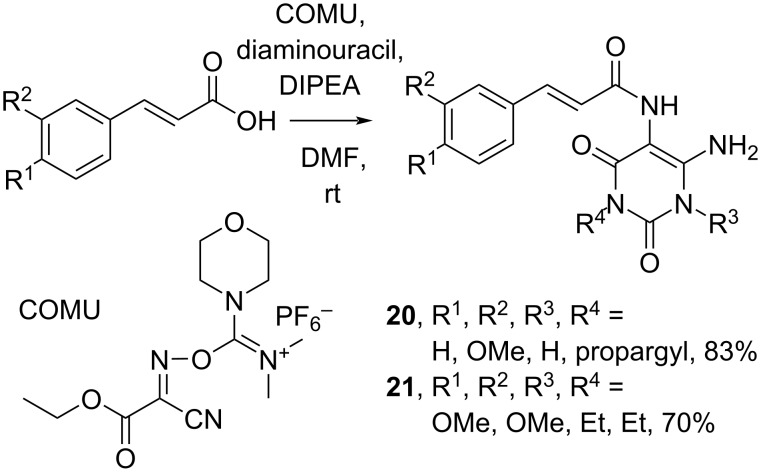
Cinnamic acid amidation using COMU as coupling reagent.

Müller and co-workers (2019) reported the synthesis of 6-amino-5-carboxamidouracil derivatives **20** and **21**, precursors for A_2A_ antagonists, in good yields by using non-hazardous (1-cyano-2-ethoxy-2-oxoethylidenaminooxy)dimethylamino(morpholino)carbenium hexafluorophosphate (COMU) as the coupling reagent (Scheme 7) [38].

**Scheme 8:**
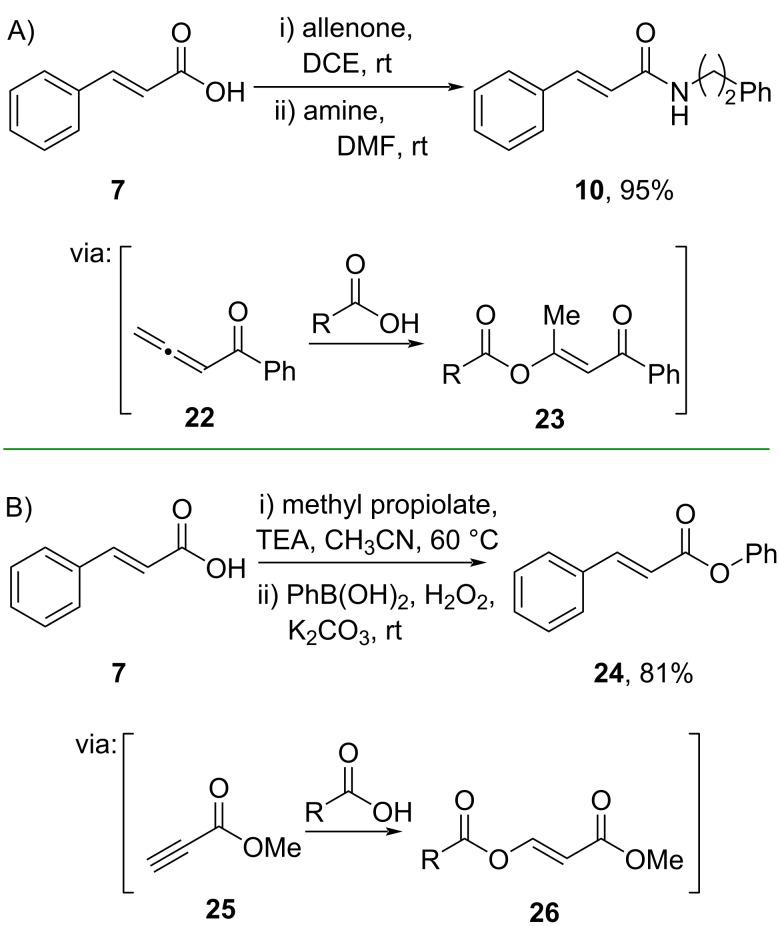
Cinnamic acid amidation using allenone coupling reagent.

By mimicking the electrophilic sp-carbon center of common coupling reagents (e.g., DCC, EDC), Zhao and co-workers (2021) employed allenone **22** as a coupling reagent for amidation [39]. Herein, cinnamic acid was smoothly converted to its corresponding amide **10** in a one-pot two-step amidation in 5 min reaction time via the formation of the isolable enol ester intermediate **23** (Scheme 8A). Similarly, Feng and co-workers (2019) studied one-pot two-step esterification of cinnamic acid (**7**) by applying electrophilic sp carbon center of methyl propiolate (**25**) as the coupling reagent via in situ formation of an active enol ester **26**. The phenol was formed in situ during the second step from phenylboronic acid oxidation utilizing H_2_O_2_ (30%) as green oxidant (Scheme 8B) [40].

**Scheme 9:**
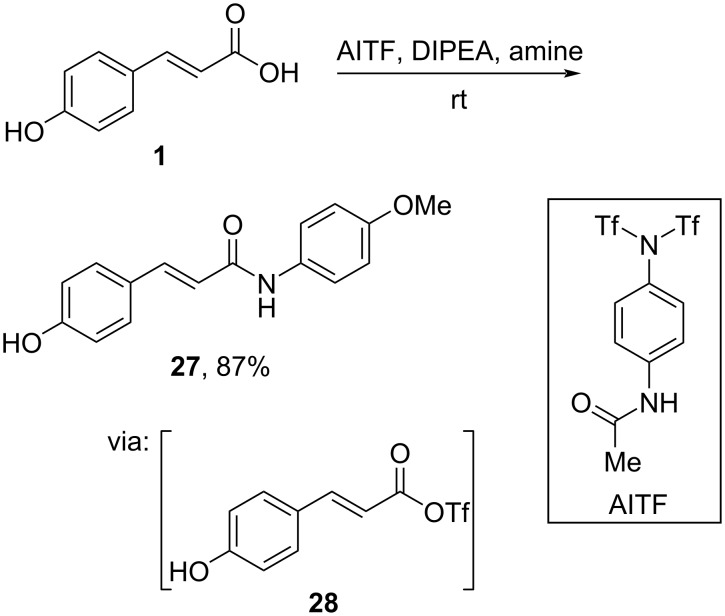
Cinnamic acid amidation using 4-acetamidophenyl triflimide as reagent.

Sureshbabu and co-workers (2023) activated the carboxyl group of 4-hydroxycinnamic acid (**1**) by selectively reacting it with a triflate surrogate, 4-acetamidophenyl triflimide (AITF), to generate the intermediate reactive acyl triflic anhydride **28** which afforded the corresponding amide **27** in good yield (Scheme 9) [41].

**Scheme 10:**
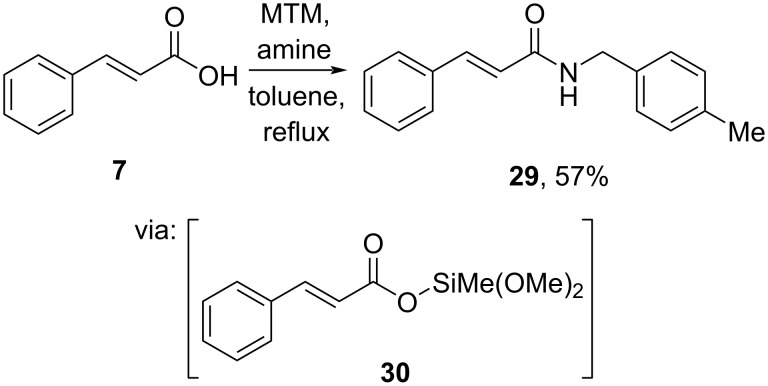
Cinnamic acid amidation using methyltrimethoxysilane (MTM).

On the other hand, Braddock and co-workers (2022) employed methyltrimethoxysilane (MTM) to activate the carboxyl group of cinnamic acid (**7**) to generate the reactive silyl ester **30**, which converted to the corresponding amide **29** upon reaction with an amine on a gram-scale operation (Scheme 10) [42].

**Scheme 11:**
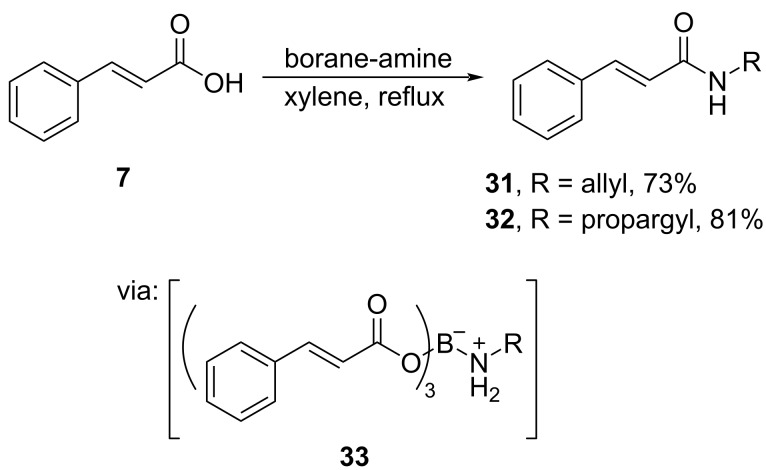
Cinnamic acid amidation utilizing amine–borane reagent.

Ramachandran and co-workers (2020) performed the amidation of cinnamic acid (**7**) by utilizing stoichiometric amine–BH_3_ reagent via triacyloxyborane–amine complex **33** to obtain the corresponding amides **31** and **32** in good yields (Scheme 11) [43].

**Scheme 12:**
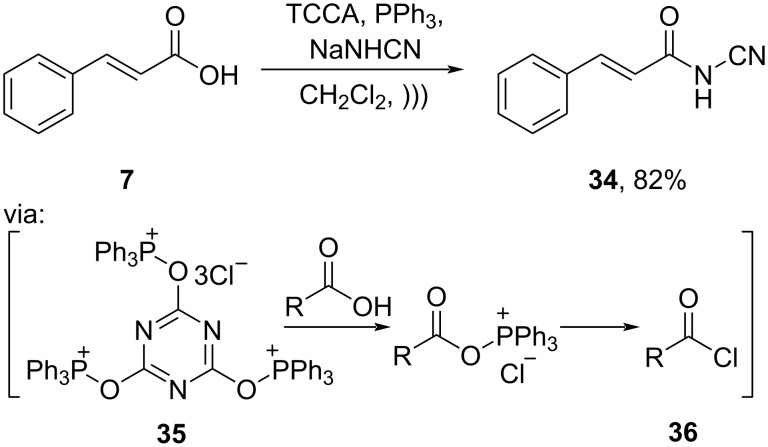
Cinnamic acid amidation using TCCA/PPh_3_ reagent.

Moreover, acid halides are widely applied for esterification and amidation. For example, Pattarawarapan and co-workers (2020) reported the amidation of cinnamic acid (**7**) by using trichloroisocyanuric acid/triphenylphosphine (TCCA/PPh_3_) assisted by ultrasound to give the corresponding amide **34** in good yield (Scheme 12) [44]. Herein, PPh_3_ attacks chloride atoms in TCCA to subsequently generate phosphonium intermediate **35**, followed by the formation of reactive acid chloride **36**.

**Scheme 13:**
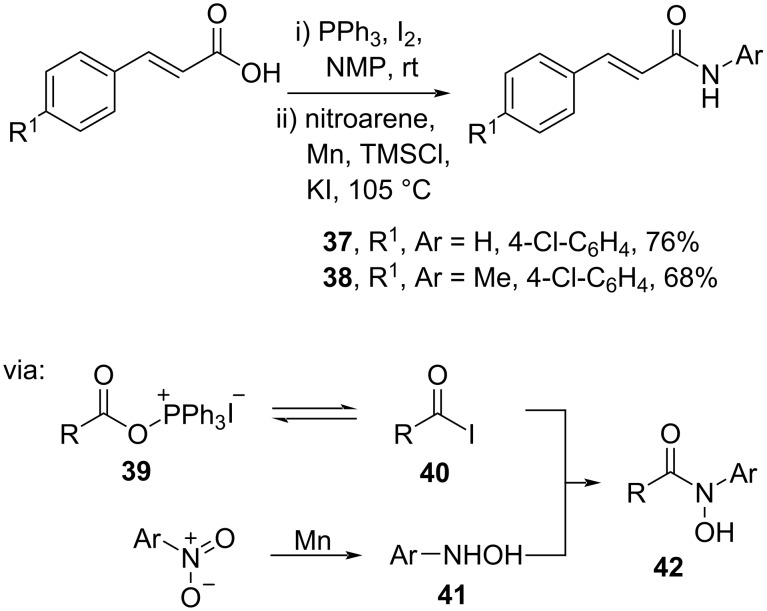
Cinnamic acid amidation using PPh_3_/I_2_ reagent.

On the other hand, Ma and co-workers (2019) converted cinnamic acids to the corresponding amides **37** and **38** in good yields via the active acyloxyphosphonium iodide species **39** in equilibrium with the acid iodide **40** (Scheme 13) [45]. Subsequently, **39/40** was coupled with **41** prepared via in situ nitro reduction using Mn powder to afford the amide intermediate **42**.

**Scheme 14:**
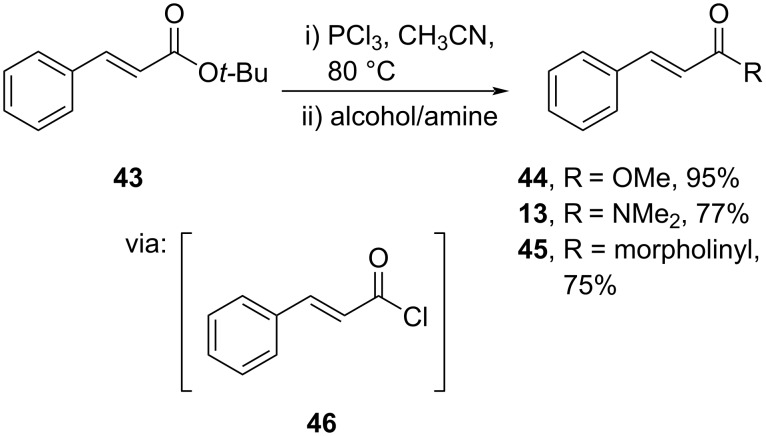
Cinnamic acid amidation using PCl_3_ reagent.

The acid halide once again was applied for cinnamic acid amidation. Xiao and co-workers (2021) performed a transesterification and aminolysis of the *tert*-butyl ester **43** simply by using PCl_3_ through in situ generation of the active acid chloride **46** to obtain the corresponding ester **44** and amide products **13** and **45** in good yields (Scheme 14) [46]. In addition, this method has successfully been conducted in gram-scale reactions.

**Scheme 15:**
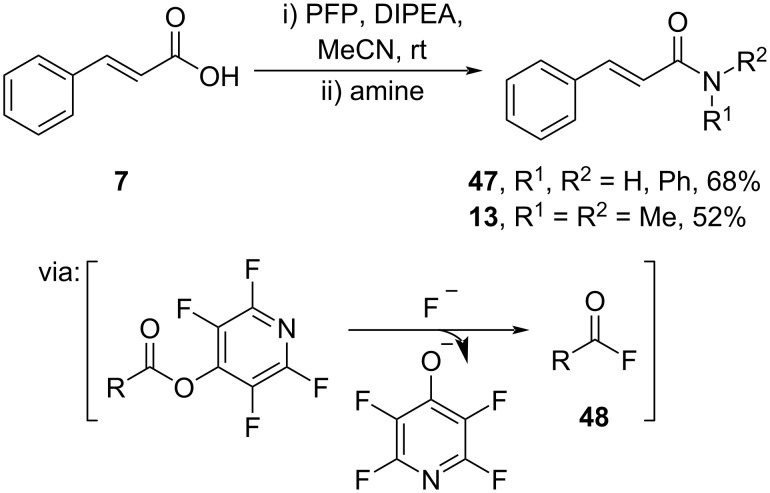
Cinnamic acid amidation utilizing pentafluoropyridine (PFP) as reagent.

Cobb and Brittain (2021) reported the amidation of cinnamic acid (**7**) by applying electrophilic pentafluoropyridine (PFP) via in situ formation of an active acid fluoride **48** to afford the corresponding amides **13** and **47** in moderate yields (Scheme 15) [47]. In addition, the amidation process could be scaled up to a gram scale to give **47** in 90% yield.

**Scheme 16:**
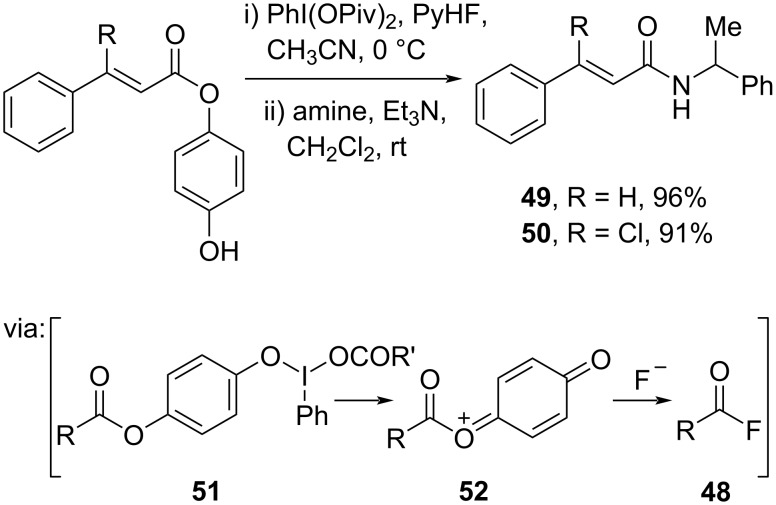
Cinnamic acid amidation using hypervalent iodine(III).

Maruoka and co-workers (2021) converted cinnamic esters into active acid fluorides **48** by utilizing hypervalent iodine(III) of PhI(OPiv)_2_ and py·HF as the fluoride source to afford the corresponding amides **49** and **50** in excellent yields (Scheme 16) [48]. Herein, the hypervalent iodine(III) reagent reacted with the phenol group to give intermediates **51** and **52** followed by the fluoride attack.

**Scheme 17:**
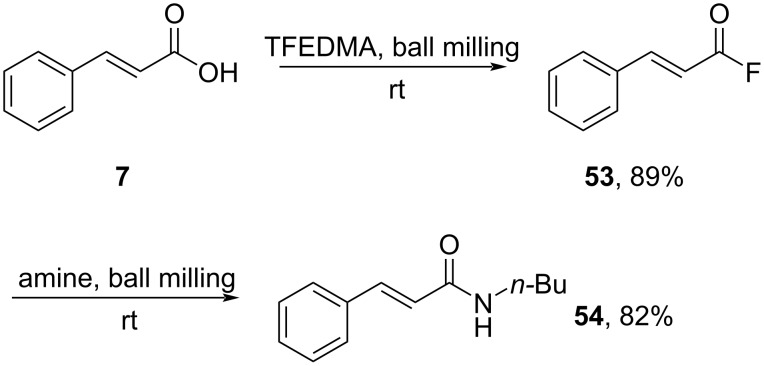
Mechanochemical amidation using 1,1,2,2-tetrafluoroethyl-*N*,*N*-dimethylamine (TFEDMA) reagent.

Shibata and co-workers (2024) developed an amidation process by utilizing 1,1,2,2-tetrafluoroethyl-*N*,*N*-dimethylamine (TFEDMA) proceeding via an active acid fluoride in a mechanochemical fashion [49]. In this method, cinnamic acid was reacted with TFEDMA under solvent-free conditions to afford the corresponding acid fluoride **53** in good yield. This was followed by the amidation step to give the corresponding amide **54** in good yield (Scheme 17). The method was also conducted on a gram-scale operation.

**Scheme 18:**
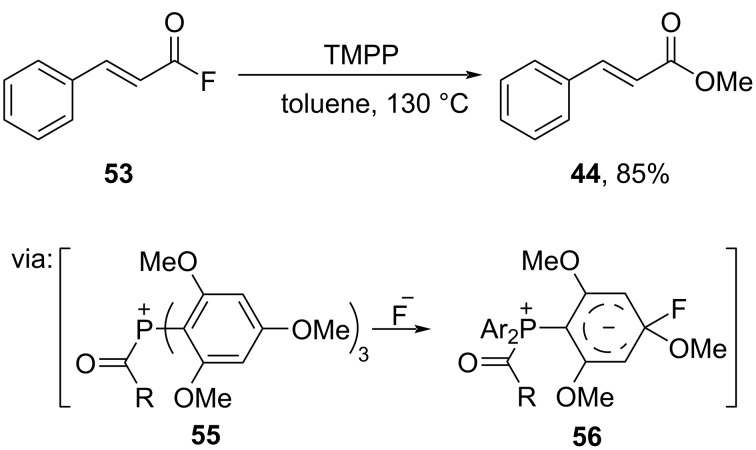
Methyl ester preparation using tris(2,4,6-trimethoxyphenyl)phosphine (TMPP).

Nishihara and co-workers (2020) performed methoxylation of acid fluoride **53** by using tris(2,4,6-trimethoxyphenyl)phosphine (TMPP) to obtain methyl cinnamate (**44**) in good yield (Scheme 18) [50]. Herein, the phosphine group attacks the acid fluoride to give intermediate **55**, fluoride attack then triggers methoxy group release.

**Scheme 19:**
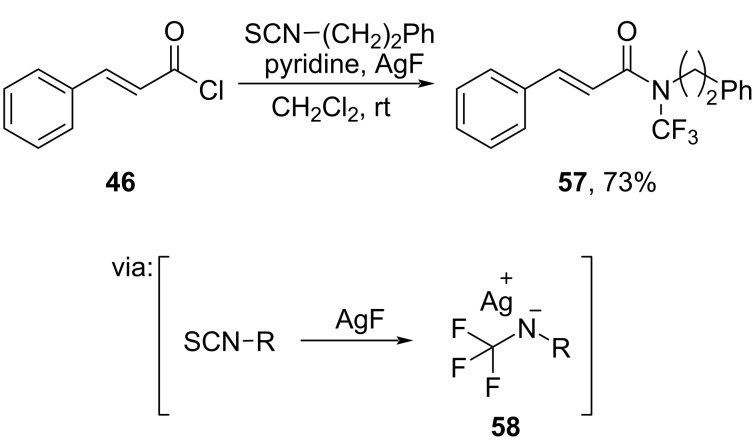
*N*-Trifluoromethyl amide preparation using isothiocyanate and AgF.

Toste and co-workers (2021) synthesized *N*-trifluoromethyl amide **57** from the corresponding acid chloride **46** by employing isothiocyanate in the presence of AgF (Scheme 19) [51]. Herein, fluoride ions attack the isothiocyanate to afford the reactive *N*-trifluoromethylated secondary amine intermediates **58**.

**Scheme 20:**
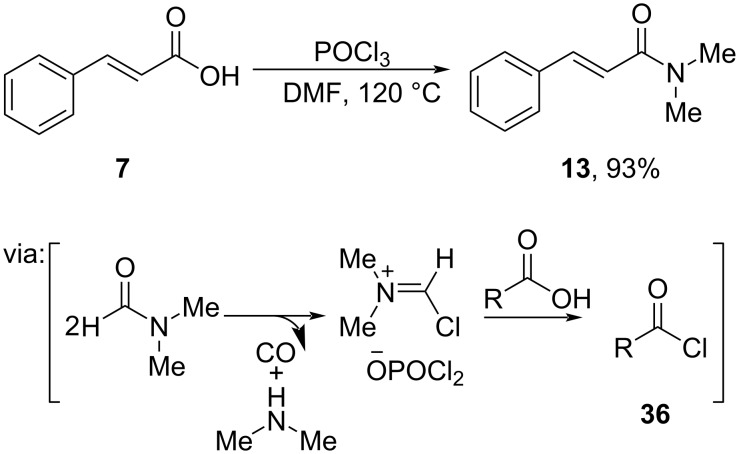
POCl_3_-mediated amide coupling of carboxylic acid and DMF.

Xiao and co-workers (2019) prepared cinnamamide **13** from cinnamic acid (**7**) and *N*,*N’*-dimethylformamide (DMF)-mediated by POCl_3_ via acid chloride **36** formation (Scheme 20) [52].

**Scheme 21:**
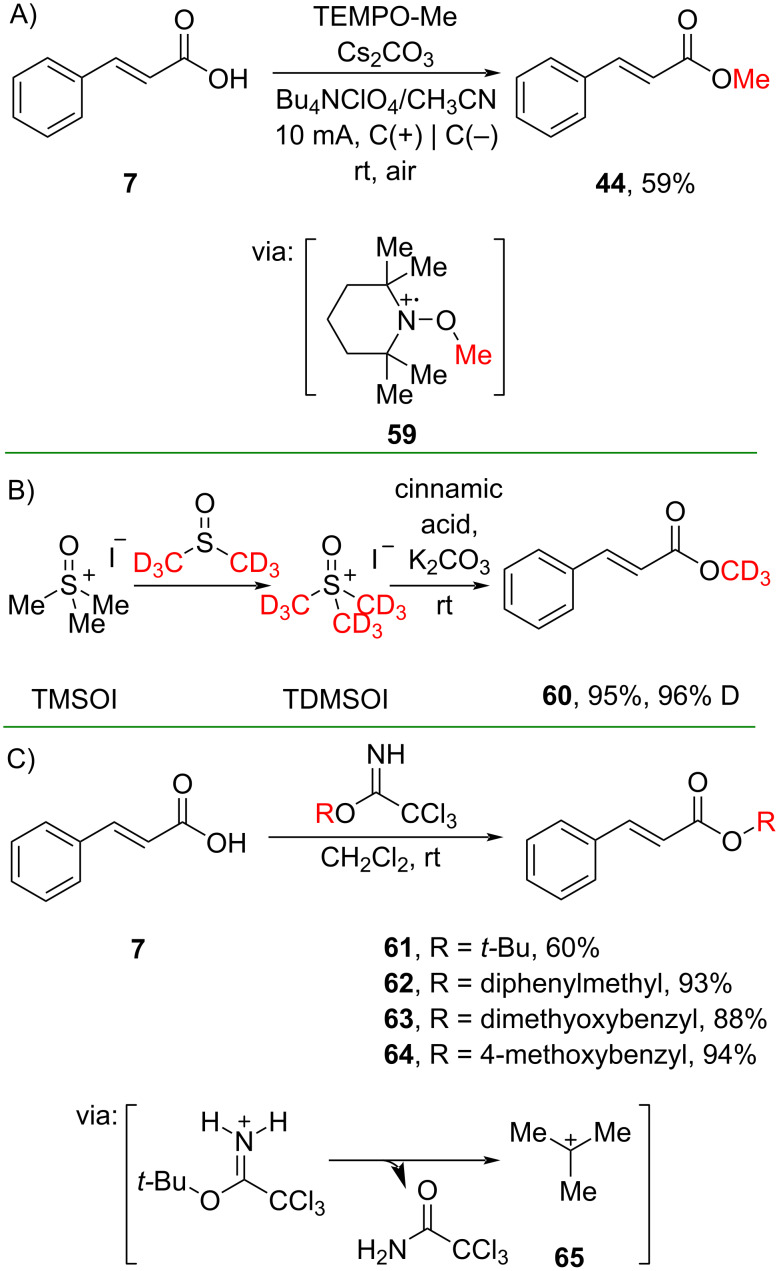
*O*-Alkylation of cinnamic acid using alkylating agents.

On top of the carboxyl activation approaches demonstrated above, *O*/*N*-acylation could also be achieved by employing an electrophilic alkylating agent by exploiting the nucleophilicity of the O atom of the carboxylate group. For instance, Coote and co-workers (2019) reported electrochemical methylation of cinnamic acid **7** using the TEMPO-Me reagent via reactive radical cation **59** to give the corresponding methyl ester **44** in moderate yield (Scheme 21A) [53]. Wang and co-workers (2019) studied a novel trideuteromethylation reagent, trideuteromethylsulfoxonium iodide (TDMSOI), to convert cinnamic acid (**7**) into its corresponding trideuteromethyl ester **60** in a one-pot two-step setup with excellent yield and D incorporation (Scheme 21B) [54]. The reagent was prepared by reacting trimethylsulfoxonium iodide (TMSOI) with DMSO-*d*_6_, resulting in CH_3_/CD_3_ exchange. Furthermore, Chisholm and co-workers (2019) synthesized bulky cinnamate esters **61**–**64** utilizing a trichloroacetimidate-based alkylating agent in moderate to excellent yields via carbocation **65** formation upon trichloroacetamide release (Scheme 21C) [55].

**Scheme 22:**
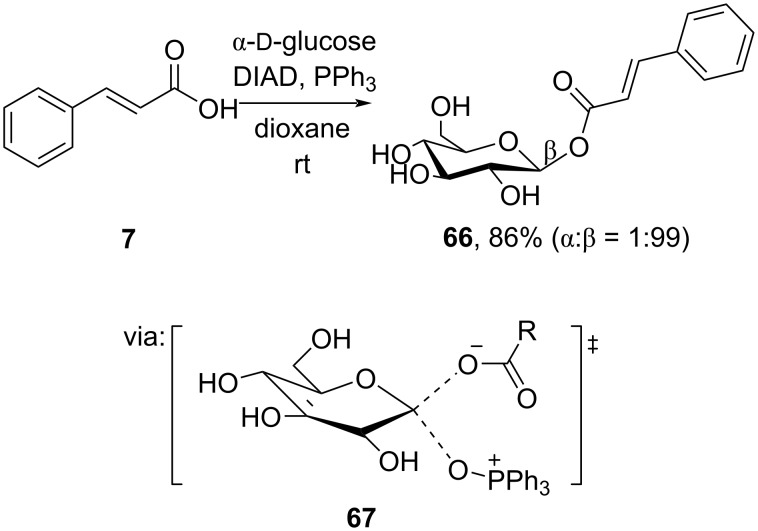
Glycoside preparation via Mitsunobu reaction.

Kawabata and co-workers (2020) prepared the β-glycoside **66** from α-ᴅ-glucose and cinnamic acid (**7**) in good yield through the Mitsunobu reaction (Scheme 22) [56]. The ^13^C kinetic isotope effect experiment (KIE = 1.028) showed that the glycosylation proceeded via S_N_2 substitution (**67**).

**Scheme 23:**
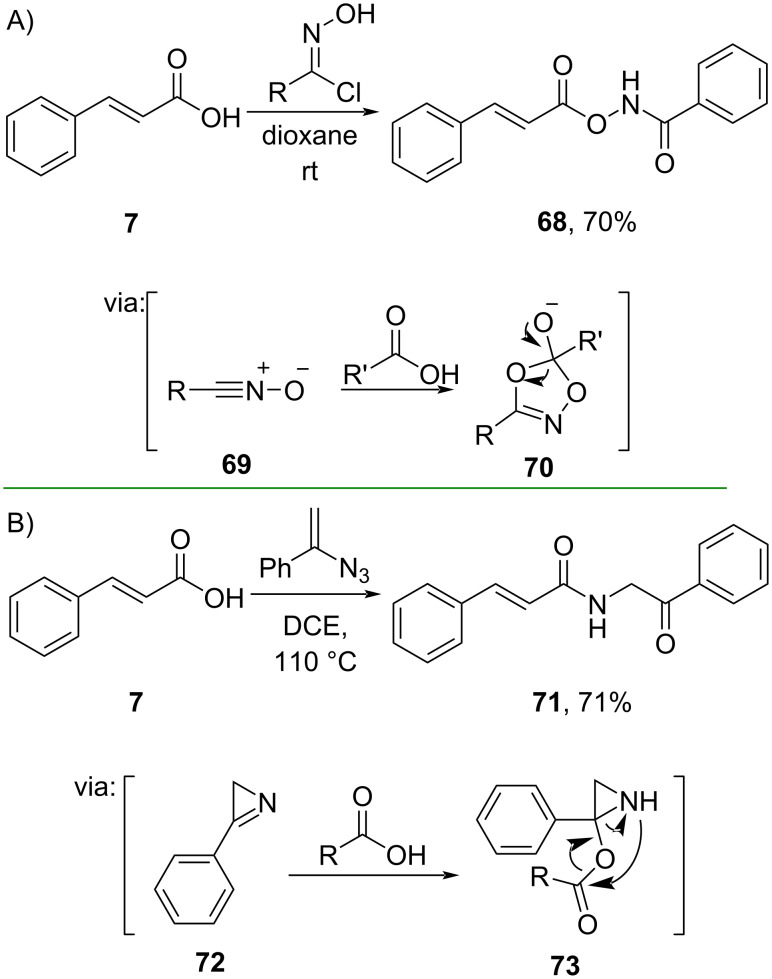
*O*/*N*-Acylation via rearrangement reactions.

Sun and co-workers (2021) utilized oxime chloride and cinnamic acid to synthesize *O*-acylhydroxamate **68** in good yield (Scheme 23A) [57]. In the presence of a base, oxime chloride was converted to electrophilic nitrile oxide **69** and reacted with carboxylic acid to form the cyclic intermediate **70**. On the other hand, Fan and co-workers (2020) prepared α-amidoketone **71** by employing vinyl azide and cinnamic acid (**7**) in good yield via cascade reaction (Scheme 23B) [58]. The thermal decomposition of the azide led to the generation of the reactive azirine intermediate **72**.

**Scheme 24:**
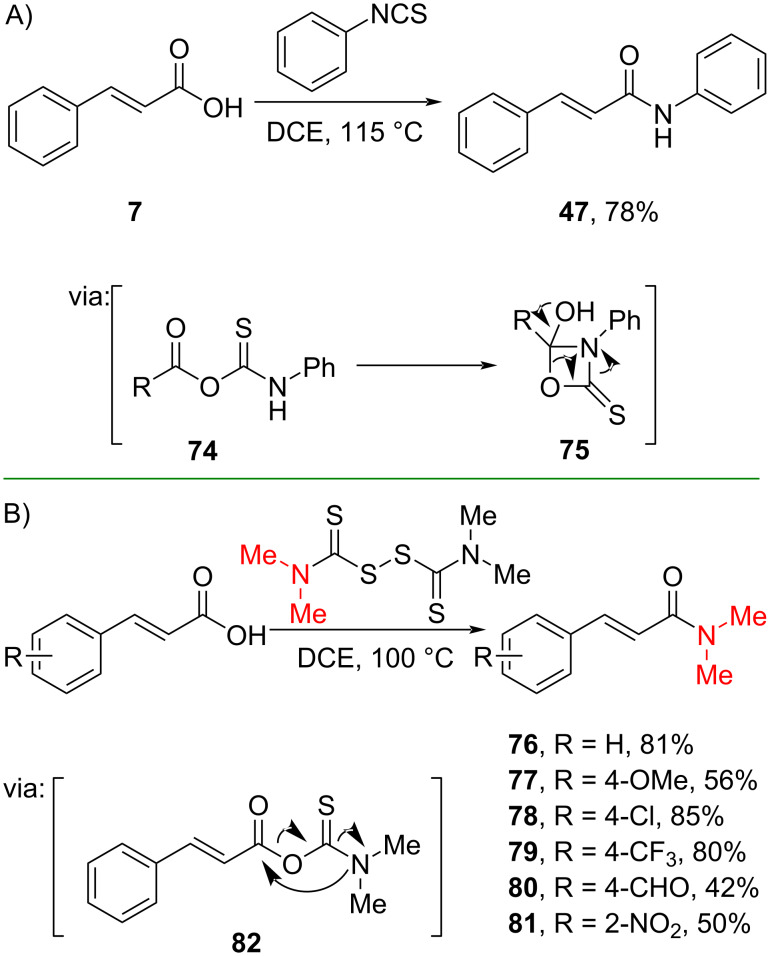
Amidation reactions using sulfur-based alkylating agents.

Moreover, Li and co-workers (2020) utilized isothiocyanate and cinnamic acid (**7**) to prepare the corresponding amide **47** in good yield via a carbamothioic anhydride **74** formation followed by carbonyl sulfide (COS) release (Scheme 24A) [59]. Similarly, Zhao and co-workers (2020) employed tetraalkylthiuram disulfides to synthesize amides via COS release (**82**). Numerous cinnamic acid derivatives with electron-withdrawing and -donating groups were converted to the corresponding amides **76**–**81** in moderate to excellent yields (Scheme 24B) [60].

**Scheme 25:**
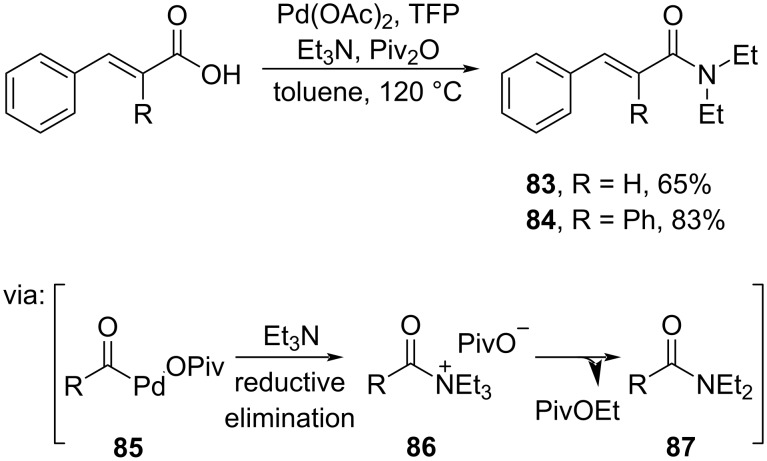
Amidation reaction catalyzed by Pd^0^ via C–N cleavage.

**2.1.2 Transition-metal catalysis:** Several transition metals have been exploited to catalyze *O*/*N*-acylations of cinnamic acid. For example, Chen and co-workers (2020) reported the Pd-catalyzed *N*-acylation of cinnamic acids using tertiary amines to generate the corresponding amides **83** and **84** in good yields via C–N cleavage (Scheme 25) [61]. The active Pd^0^ species was inserted into the carboxylate group to afford the intermediate **85** followed by reductive elimination with tertiary amine to give intermediate **86**.

**Scheme 26:**
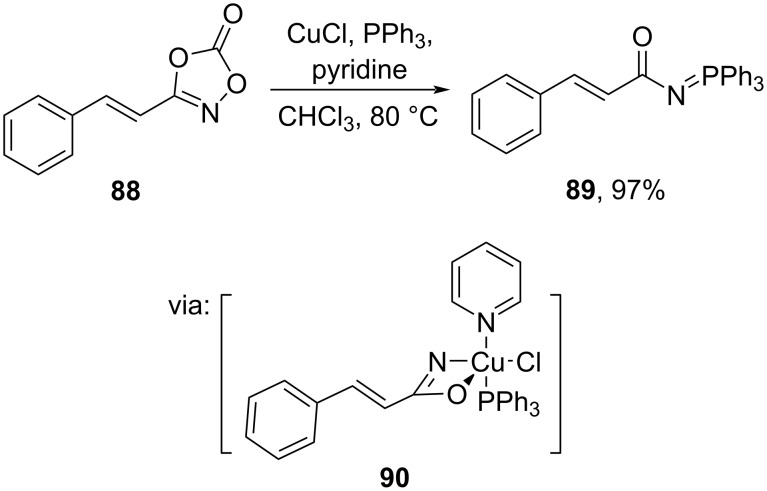
Amidation reaction catalyzed by CuCl/PPh_3_.

The utilization of earth-abundant transition metals for *O*/*N*-acylation has emerged due to their low cost. For instance, Son and co-workers (2023) utilized a more cost-efficient Cu salt to access *N*-acyliminophosphorane **89** from the corresponding dioxazolone **88** in excellent yields via reductive elimination from intermediate **90** (Scheme 26) [62].

**Scheme 27:**
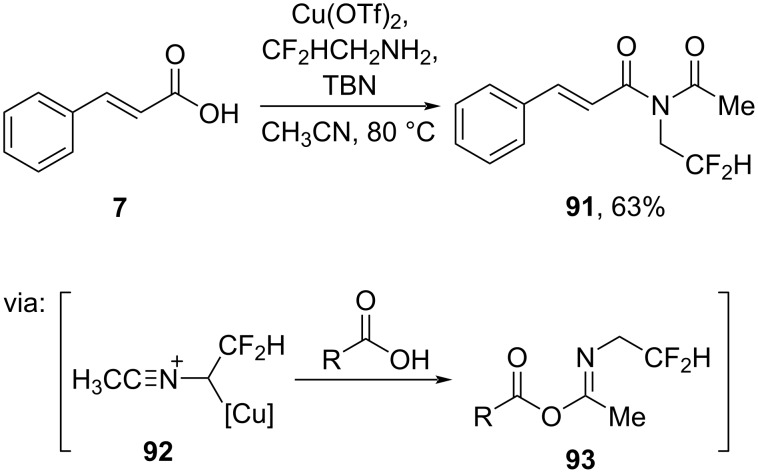
Cu(II) triflate-catalyzed *N*-difluoroethylimide synthesis.

Hu and co-workers (2019) also employed a Cu salt (Cu(OTf)_2_) to synthesize *N*-difluoroethylimide **91** from cinnamic acid (**7**) and *tert*-butyl nitrite (TBN) in good yield via nitrilium salt **92** followed by carboxylate attack (**93**) and Mumm rearrangement (Scheme 27) [63].

**Scheme 28:**
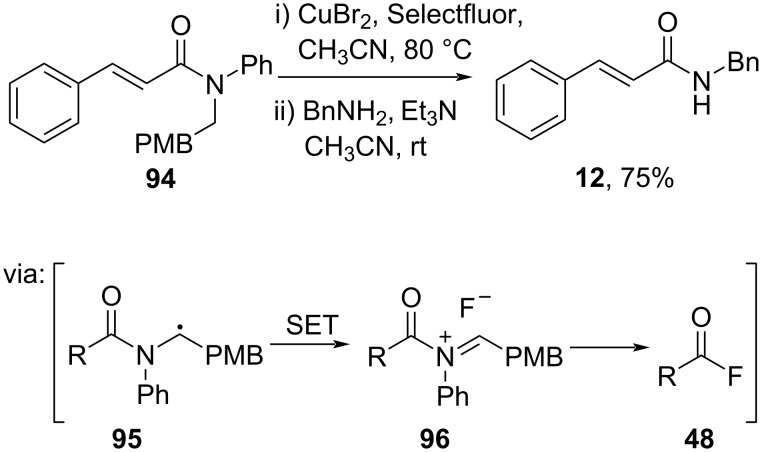
Cu/Selectfluor-catalyzed transamidation reaction.

Furthermore, Maruoka and co-workers (2020) developed a one-pot transamidation reaction catalyzed by Cu via acid fluoride **48** (Scheme 28) [64]. In this work, single-electron transfer (SET) between Selectfluor and CuBr promoted hydrogen atom abstraction from the amide **94** resulting in the benzylic radical species **95**, followed by oxidation to give acyliminium species **96**.

**Scheme 29:**
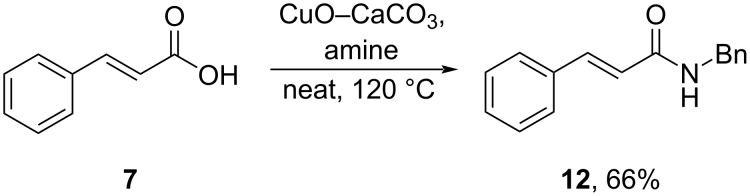
CuO–CaCO_3_-catalyzed amidation reaction.

Luque and co-workers (2020) developed a biogenic carbonate of CuO–CaCO_3_ to catalyze solvent- and additive-free amidation reactions in air, promoting ecocompatibility by minimizing waste. In this study, Cu-incorporated CaCO_3_ catalyzed the amidation of cinnamic acid (**7**) to give the corresponding amide **12** in good yield (Scheme 29) [65].

**Scheme 30:**
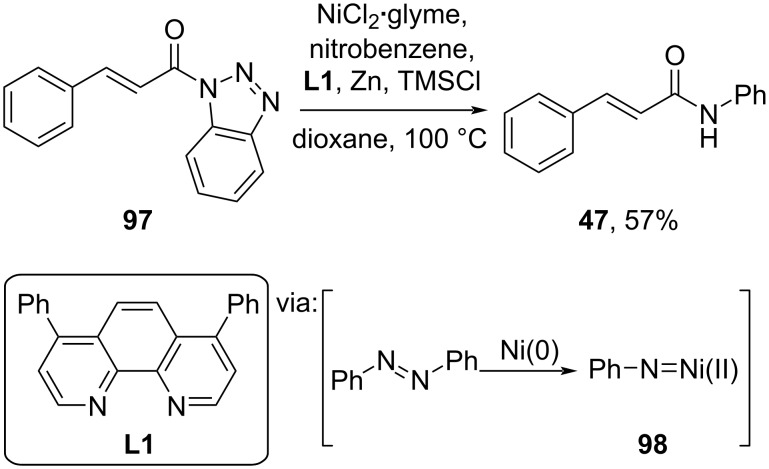
Ni-catalyzed reductive amidation.

Moreover, Li and co-workers (2022) employed a Ni salt to catalyze the reductive amidation of nitrobenzene and *N*-acylbenzotriazole **97** via the Ni(II)-nitrene species **98** to afford its corresponding amide **47** in moderate yield (Scheme 30) [66].

**Scheme 31:**
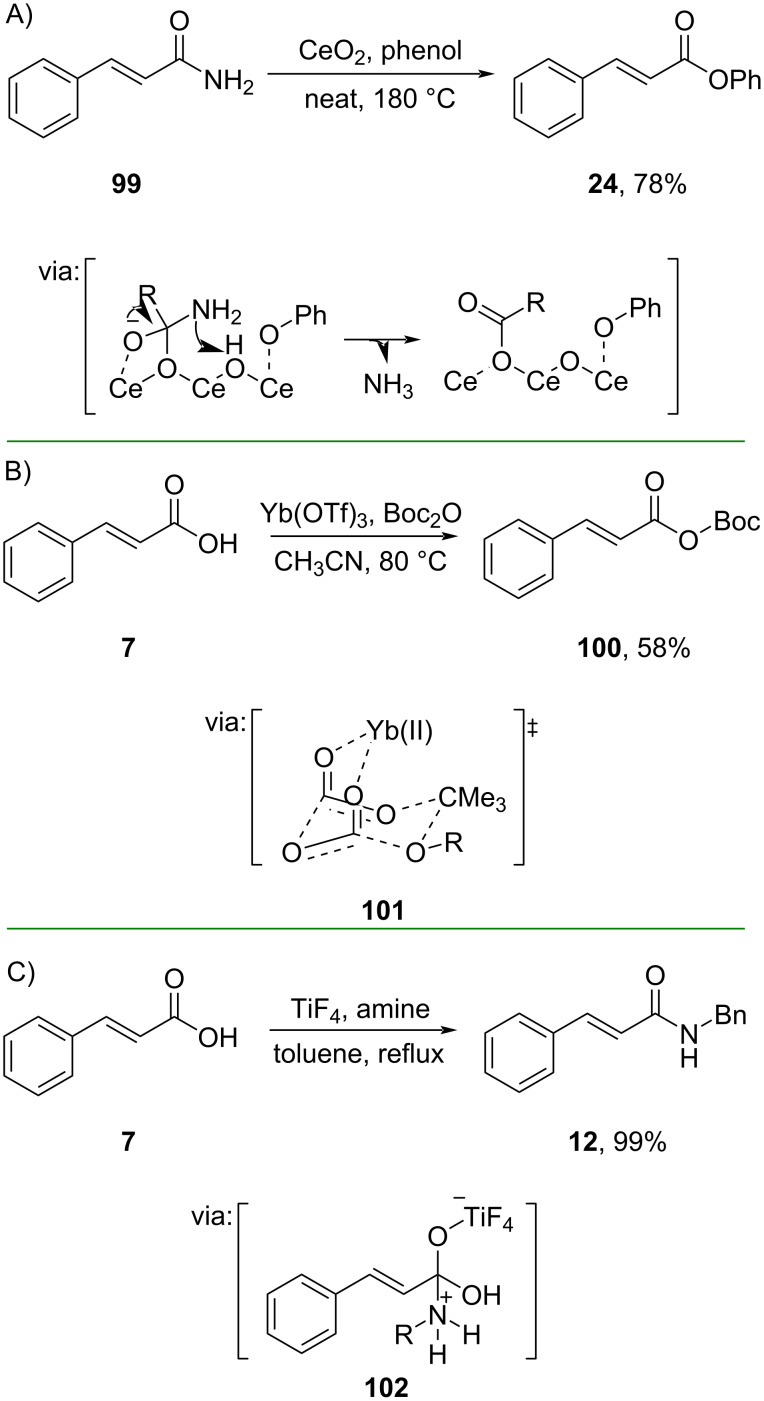
Lewis acidic transition-metal-catalyzed *O*/*N*-acylations.

Shimizu and co-workers (2019) reported a CeO_2_-catalyzed esterification of unactivated cinnamamide (**99**) and phenol in solvent-free conditions to afford the corresponding amide **24** in good yield. The reaction proceeds via a CeO_2_-coordinated carboxylate mode (Scheme 31A) [67]. In addition, the catalyst also offered high reusability for up to 4 runs thus further promoting the eco-friendliness.

Carbonyl activation via Lewis acid–O=C interaction has also been achieved using other transition metals, such as Yb and Ti. Salunke and co-workers (2020) utilized Yb(OTf)_3_ to catalyze the Boc-protection of cinnamic acid (**7**) via the formation of the chelate complex **101** between Boc_2_O and Yb(OTf)_3_ (Scheme 31B) [68]. Recently, Ramachandran and Alawaed (2024) reported a Ti-catalyzed direct amidation of cinnamic acid (**7**) to give amide **12** in excellent yield via Ti(IV)–O=C complex **102** (Scheme 31C) [69].

**Scheme 32:**
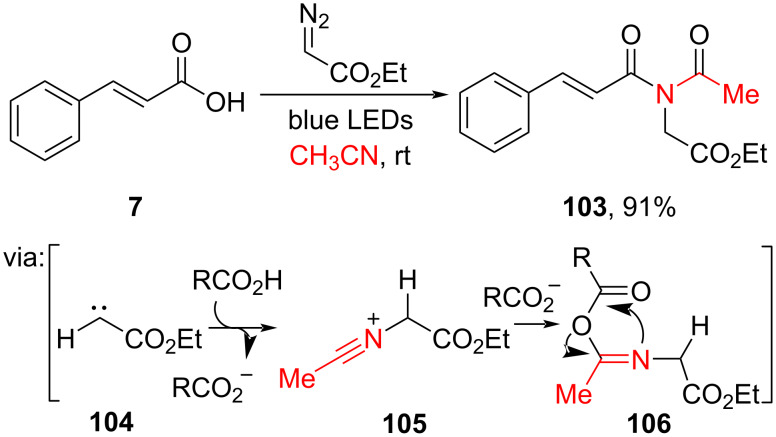
Visible-light-promoted amidation of cinnamic acid.

**2.1.3 Photocatalysis:** Photoredox catalysis has gained much attention as a sustainable alternative approach to performing *O*/*N*-acylation by utilizing light as a renewable source. For example, Li and co-workers (2022) investigated the visible-light-mediated amidation of cinnamic acid (**7**) by using ethyl 2-diazoacetate and acetonitrile to give its corresponding amide **103** in excellent yield (Scheme 32) [29]. The reactive free carbene **104** was released upon light exposure of the diazo ester leading to the nitrilium ion **105** formation via its reaction with acetonitrile.

**Scheme 33:**
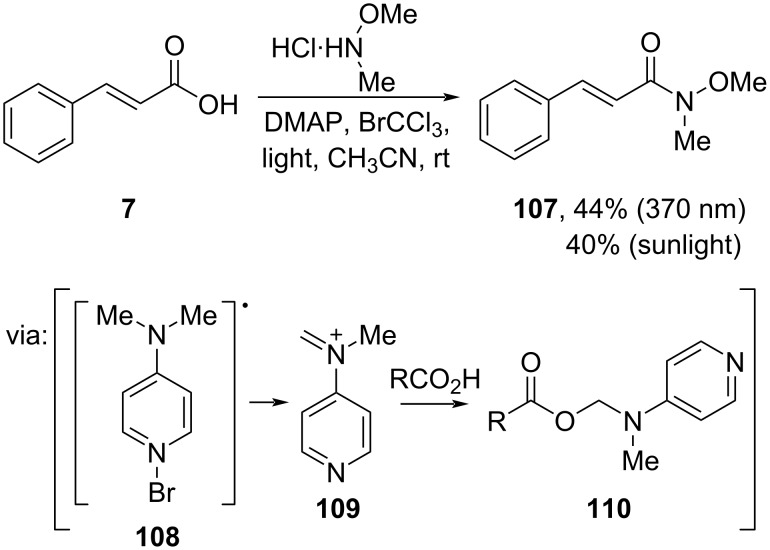
Sunlight/LED-promoted amidation of cinnamic acid.

Kokotos and co-workers (2023) prepared Weinreb amide **107** mediated by sunlight or LED 370 nm from cinnamic acid (**7**) via light-activated DMAP **108**, leading to electrophilic iminium **109** formation (Scheme 33) [28].

**Scheme 34:**
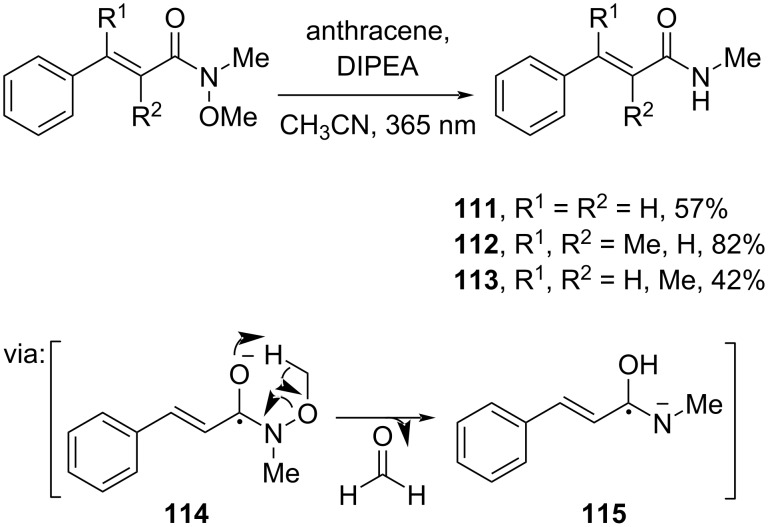
Organophotocatalyst-promoted N–O cleavage of Weinreb amides to synthesize primary amides.

On the other hand, Gilmour and co-workers (2022) functionalized Weinreb amides through organophotocatalytic N–O cleavage via **114** and **115** to give the corresponding primary amides **111**–**113** in good yields (Scheme 34) [27].

**Scheme 35:**
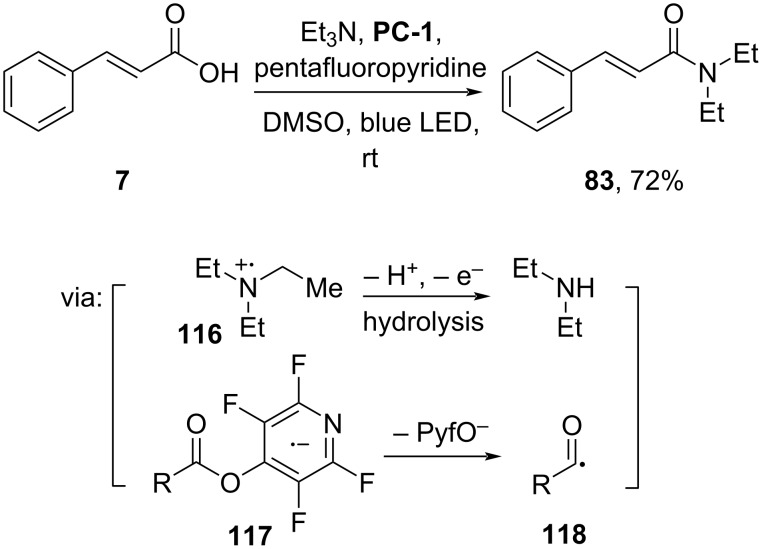
Cinnamamide synthesis through [Ir] photocatalyst-promoted C–N-bond cleavage of tertiary amines.

Xie and co-workers (2022) synthesized cinnamamide **83** mediated by [Ir(dF(CF_3_)ppy)_2_(dtbbpy)]PF_6_ (**PC-1**) as photocatalyst proceeding via C–N-bond cleavage of the oxidized tertiary amine **116** (Scheme 35) [70]. Cinnamic acid (**7**) was activated by forming the acyl radical **118** after ^−^OPyf group cleavage from **117**.

**Scheme 36:**
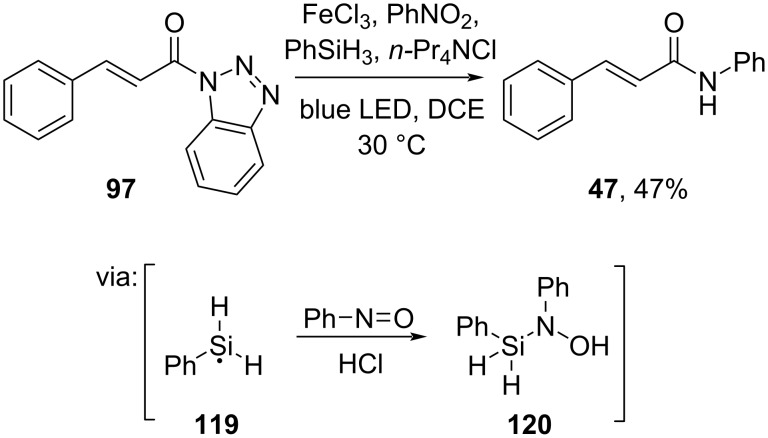
Blue LED-promoted FeCl_3_-catalyzed reductive transamidation.

Recently, Li and co-workers (2024) studied visible-light-mediated FeCl_3_-catalyzed reductive transamidation of nitro compounds and *N*-acylbenzotriazole **97** (Scheme 36) [71]. In this work, the photoactive [FeCl_4_]^−^ formed in situ triggered silyl radical **119** generation, leading to *N*-silylamine **120** as the active amine nucleophile.

**Scheme 37:**
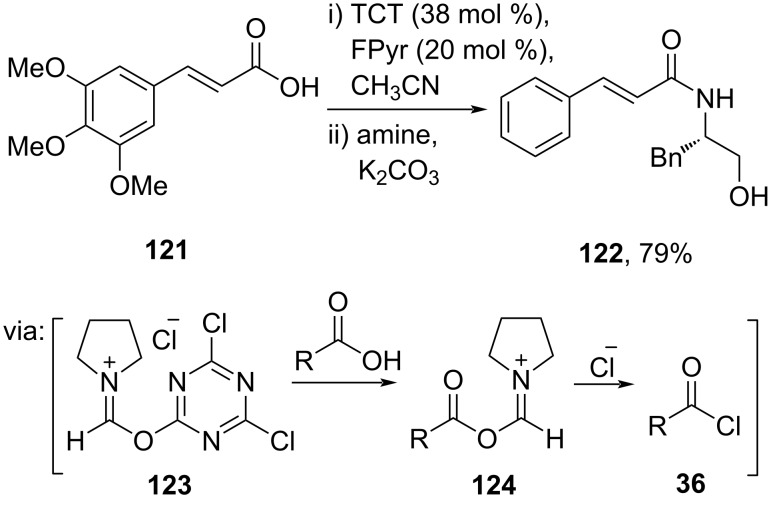
FPyr/TCT-catalyzed amidation of cinnamic acid derivative **121**.

**2.1.4 Metal-free catalysis:** Despite the wide applications of metal-based catalysts in developing *O*/*N*-acylation reactions, metal catalysts, particularly precious transition metals, are considered less sustainable due to their limited availability. Therefore, metal-free catalysis methods have emerged as an alternative to respond to the green chemistry agenda. For instance, Huy and Mbouhom (2019) employed *N*-formylpyrrolidine (FPyr) and trichlorotriazine (TCT) to catalyze the amidation of cinnamic acid derivative **121**. The reaction proceeds via formation of the reactive acid chloride **36** through cascade reaction involving reactive intermediates **123** and **124** (Scheme 37) [72].

**Scheme 38:**
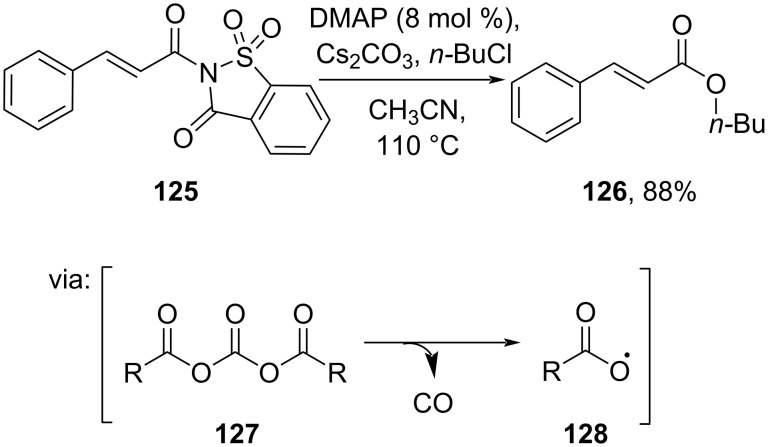
Cs_2_CO_3_/DMAP-mediated esterification.

Zeng and co-workers (2019) used activated amide **125** to access ester **126** via formation of the reactive carboxyl radical **128** mediated by DMAP and Cs_2_CO_3_ (Scheme 38) [73].

**Scheme 39:**
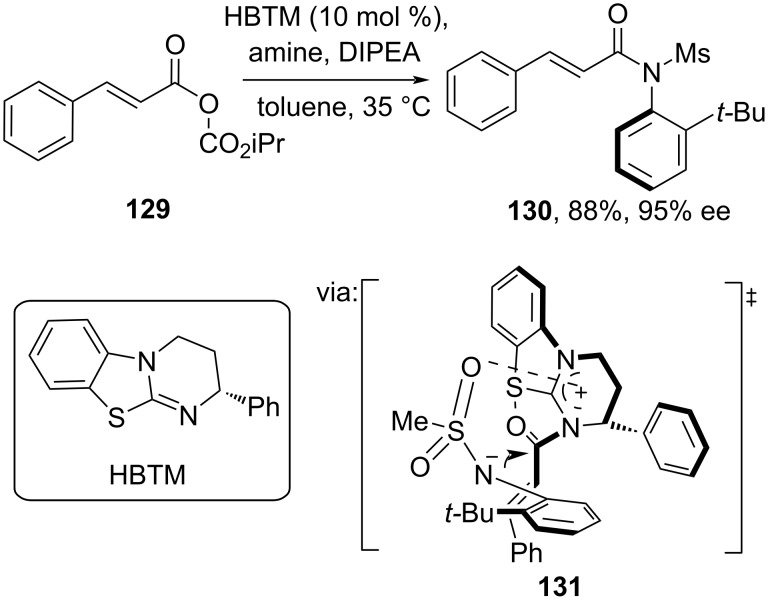
HBTM organocatalyzed atroposelective *N*-acylation.

Dong and co-workers (2020) reported an atroposelective *N*-acylation of mixed anhydride **129** catalyzed by the isothiourea organocatalyst homobenzotetramisole (HBTM) (Scheme 39) [74]. In this work, HBTM reacted with the anhydride **129** to give the acylisothiouronium intermediate followed by amine attack (**131**). The reaction capacity has been successfully increased to a gram scale. In the same year, a similar work was reported by Zhao and co-workers [75].

**Scheme 40:**
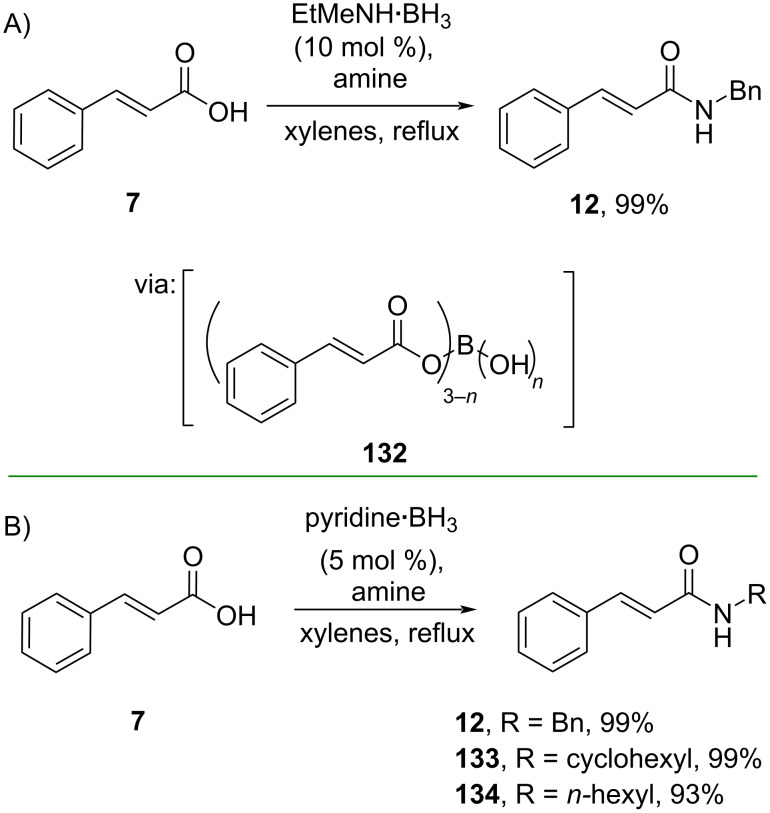
BH_3_-catalyzed *N*-acylation reactions.

On the other hand, carboxyl activation via Lewis acid–O=C interaction has also been developed. Ramachandran and Hamann (2021) directly prepared the amide **12** from cinnamic acid (**7**) catalyzed by BH_3_. The reaction proceeds via formation of the triacyloxyborane–amine complex intermediate **132** (Scheme 40A) [76]. The same group (2024) also investigated BH_3_·pyridine to catalyze amidation reactions with lower catalyst loading (Scheme 40B) [77].

**Scheme 41:**
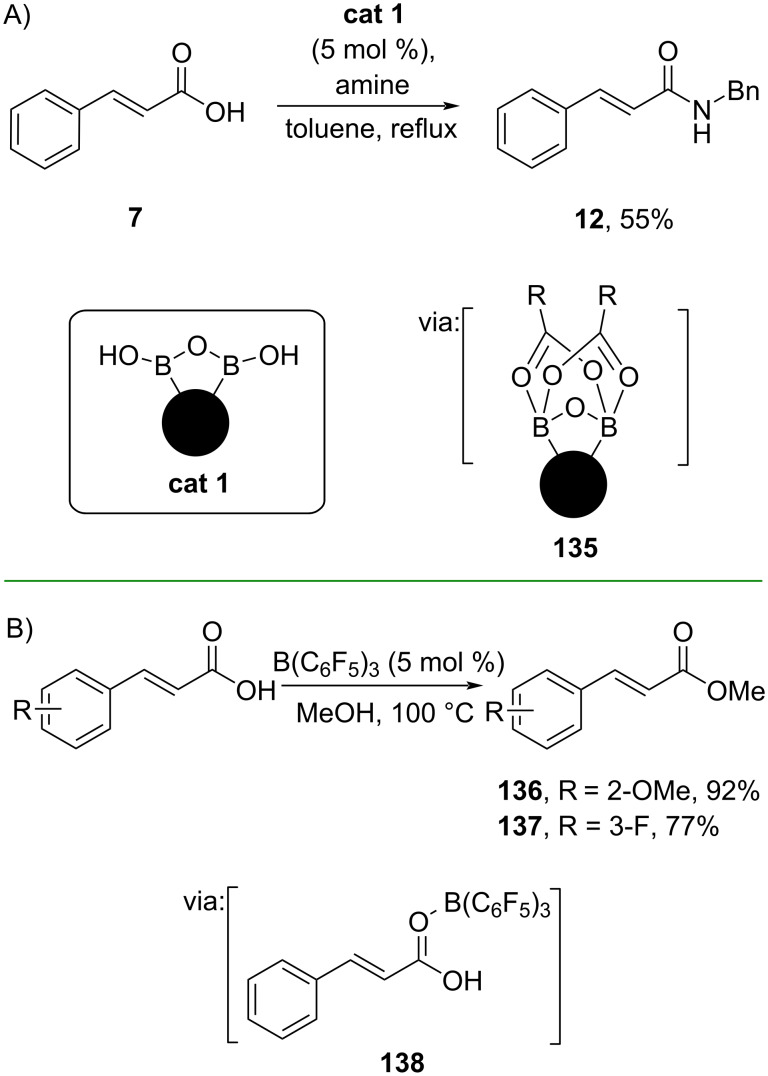
Borane-catalyzed *N*-acylation reactions.

Whiting and co-workers (2019) also used boranes to catalyze the direct amidation of carboxylic acids. In this work, they co-polymerized styrene, divinylbenzene and vinylphenylboronic acid to synthesize the solid-supported phenylboronic acid catalyst (**cat 1**) which was used to convert cinnamic acid (**7**) to its corresponding amide **12** in moderate yield. The reaction involves dicarboxylate complex **135** formed through Lewis acid B–O=C interaction (Scheme 41A) [23]. The catalyst could be reused multiple times without significant loss of activity.

Using B(C_6_F_5_)_3_ as catalyst, Wu and co-workers (2021) developed a borane-catalyzed Fischer esterification of cinnamic acids with methanol to afford the corresponding methyl cinnamates **136** and **137** via (C_6_F_5_)_3_B–O=C interaction (**138**) (Scheme 41B) [78].

**Scheme 42:**
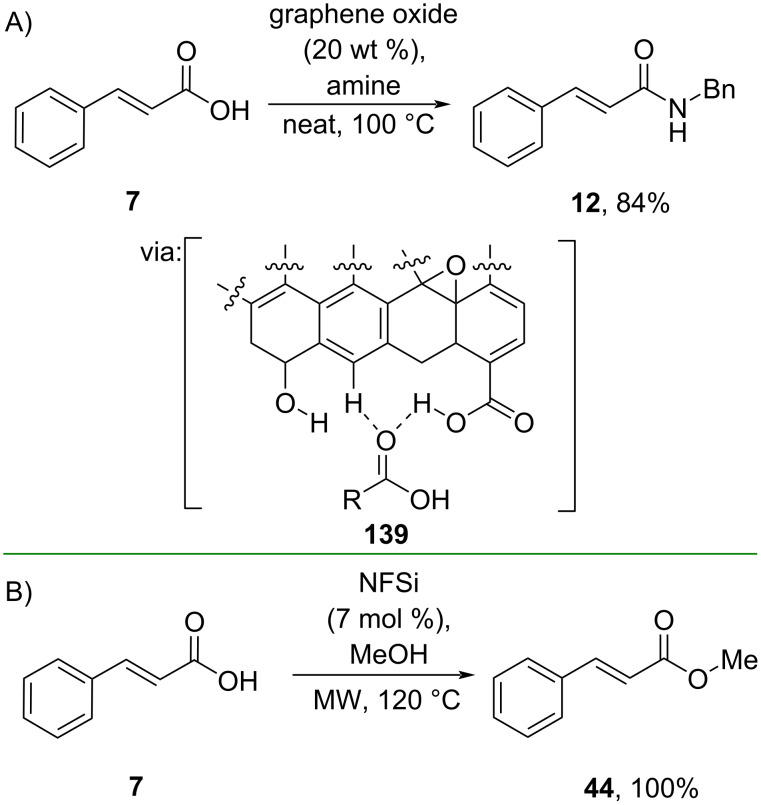
Catalytic *N*-acylation reactions via H/F bonding activation.

Shankarling and co-workers (2020) directly prepared amide **12** from cinnamic acid (**7**) under solvent-free conditions catalyzed by graphene oxide via hydrogen-bonding activation (**139**) (Scheme 42A) [79]. The catalyst could be recycled multiple times without significant activity loss. Similarly, Božić and co-workers (2022) reported the microwave-assisted direct amidation reaction of cinnamic acid (**7**) catalyzed by *N*-fluorobenzenesulfonimide (NFSi) via halogen bonding activation (Scheme 42B) [80]. In addition, the method has been successfully scaled up to a gram scale.

**Scheme 43:**
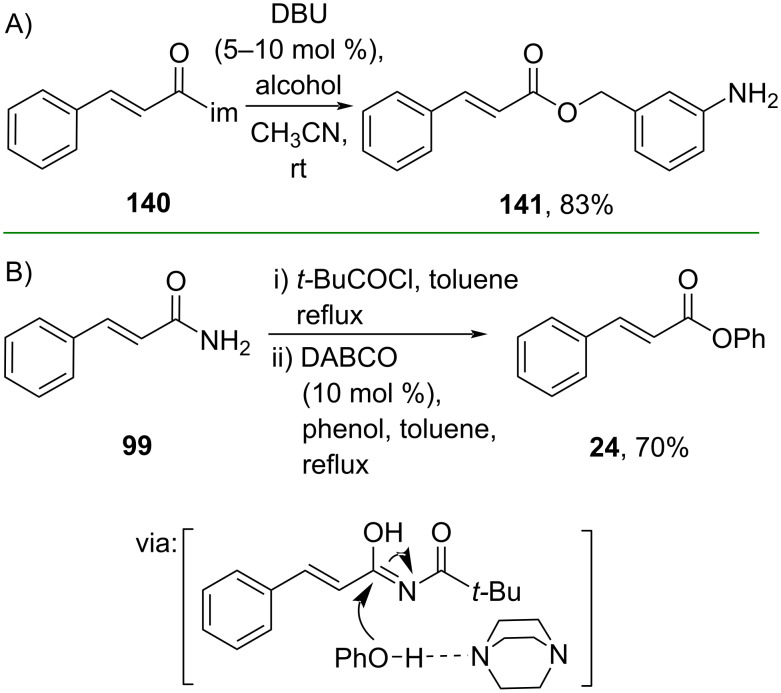
Brønsted base-catalyzed synthesis of cinnamic acid esters.

Activating a nucleophile with a catalytic base has also been applied to prepare cinnamate esters. For example, Heller and co-workers (2021) synthesized the ester **141** catalyzed by DBU which functioned as a Brønsted base for the alcohol (Scheme 43A) [81]. Impressively, the method could be scaled up to a multigram scale. Similarly, Rajendran and Rajan (2024) utilized catalytic DABCO to perform the esterification of cinnamamide **99**, where DABCO acted as a hydrogen-bond acceptor for phenol (Scheme 43B) [82].

**Scheme 44:**
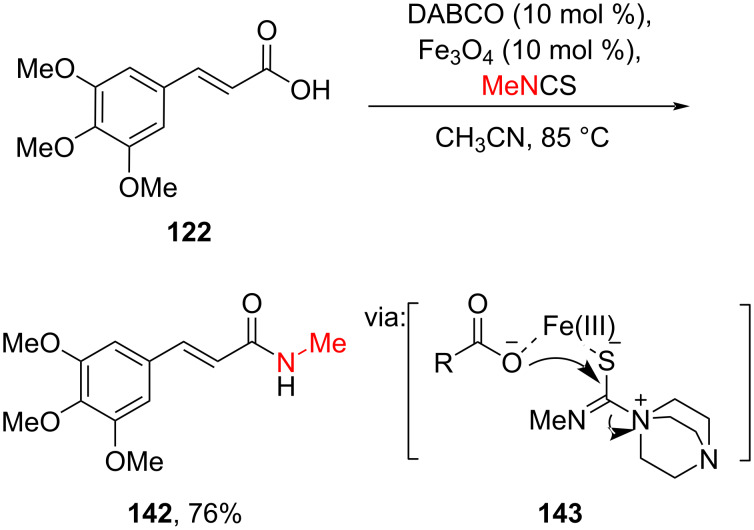
DABCO/Fe_3_O_4_-catalyzed *N*-methyl amidation of cinnamic acid **122**.

Yuan-Yong and co-workers (2022) reported a DABCO/Fe_3_O_4_-catalyzed *N*-methyl amidation of cinnamic acid **122** via cooperation reaction (**143**) to activate the isothiocyanate as the coupling partner (Scheme 44) [83].

**Scheme 45:**
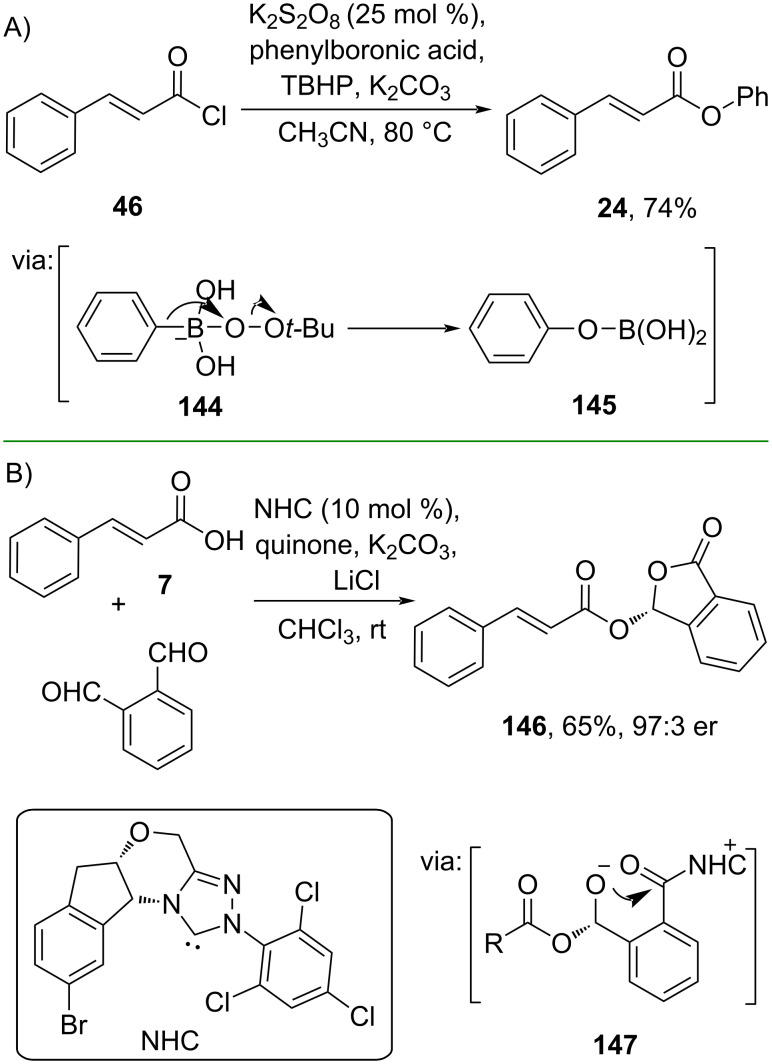
Catalytic oxidation reactions of acylating agents.

On the other hand, nucleophile activation could also be achieved via a catalytic oxidation reaction. For instance, Ablajan and co-workers (2024) reported the preparation of phenyl cinnamate (**24**) starting from the acid chloride **46** and phenylboronic acid in the presence of TBHP and catalytic amounts of K_2_S_2_O_8_. Under these conditions, phenol is formed through oxidative hydroxylation which reacts with **46** to give product **24** in good yield (Scheme 45A) [84]. The method has been scaled up to a gram scale. Similarly, Chi and co-workers (2019) reported an enantioselective esterification via oxidative *N*-heterocyclic carbene (NHC)-catalyzed phthalaldehyde activation to form azolium ester intermediate **147** to give the chiral phthalidyl ester **146** with excellent enantiomeric ratio (Scheme 45B) [85]. Additionally, the reaction capacity could be increased to a gram scale.

**Scheme 46:**
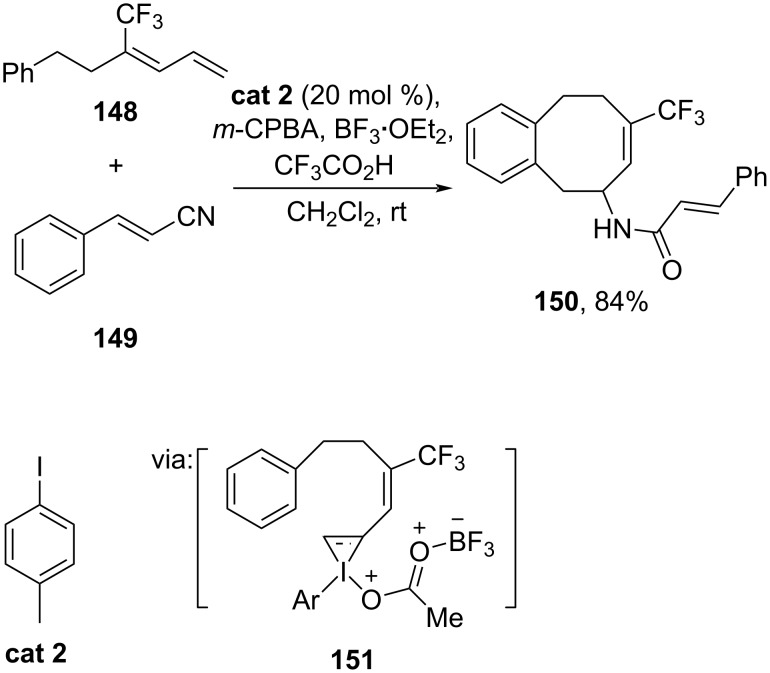
Preparation of cinnamamide-substituted benzocyclooctene using I(I)/I(III) catalysis.

Moreover, catalytic oxidation was also applied to prepare an electrophilic coupling partner. Gilmour and co-workers (2023) demonstrated the I(I)–I(III) catalytic cycle by using *p*-iodotoluene (**cat 2**) as the catalyst to activate 2-phenethyl-substituted 1,3-diene **148** with nucleophilic cinnamonitrile **149** to give benzocyclooctene **150** via iodonium intermediate **151** (Scheme 46) [86].

#### 2.2 Oxidative acylations

Cinnamic ester or amide preparation could also be achieved by oxidizing cinnamyl alcohol, aldehyde, imine, and ketone as an alternative to the traditional *O*/*N*-acylation of cinnamic acid above.

**Scheme 47:**
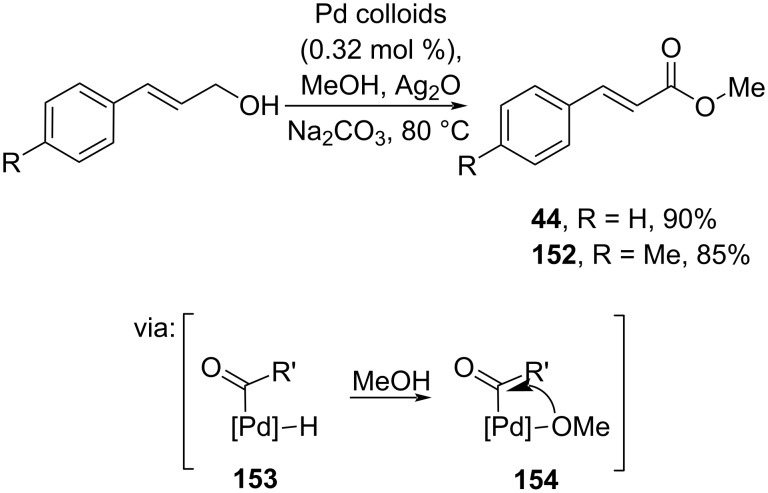
Pd-colloids-catalyzed oxidative esterification of cinnamyl alcohol.

**2.2.1 Alcohol oxidation:** Kapdi and co-workers (2019) reported Pd-colloids-catalyzed esterification via Ag_2_O-catalyzed alcohol oxidation. Herein, cinnamyl alcohols were oxidized to the corresponding cinnamaldehydes catalyzed by Ag_2_O, followed by oxidative addition to Pd via **153** and **154** to give the corresponding esters **44** and **152** driven by MeOH attack (Scheme 47) [87].

**Scheme 48:**
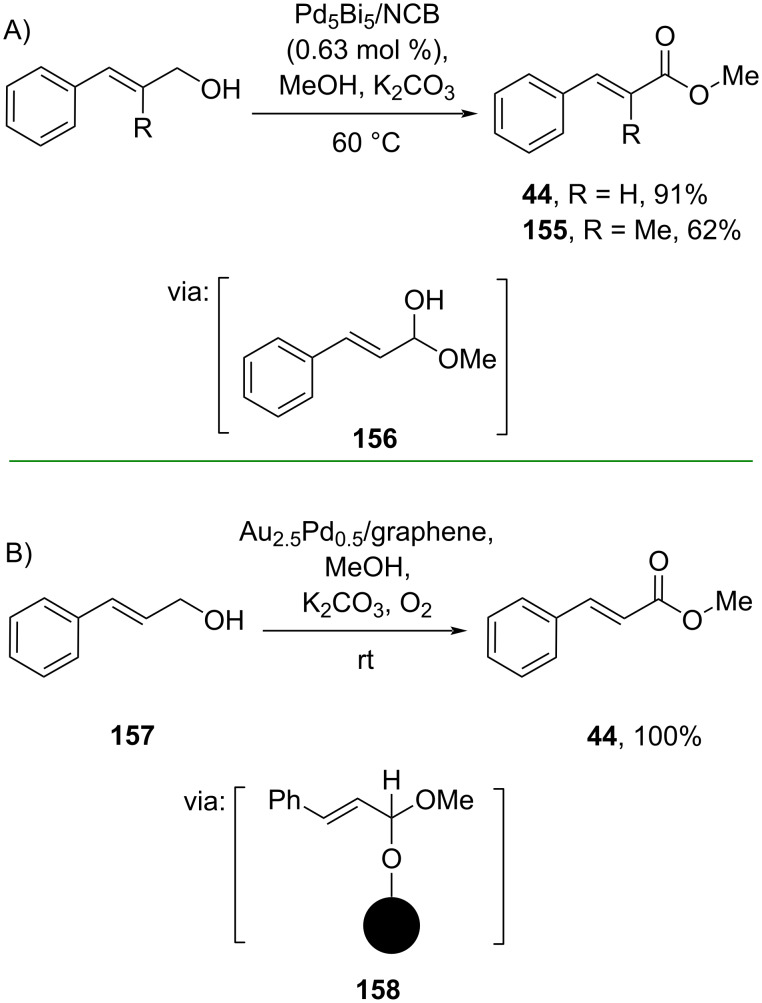
Graphene-supported Pd/Au alloy-catalyzed oxidative esterification via hemiacetal intermediate.

Hu and co-workers (2021) developed an *N*-doped carbon black-supported PdBi bimetallic catalyst (Pd_5_Bi_5_/NCB) for the oxidative esterification of cinnamyl alcohols via hemiacetal **156** oxidation (Scheme 48A) [88]. A similar reaction, reported by Zheng and co-workers (2020) utilized a graphene-supported Au/Pd catalyst to achieve the aerobic oxidative esterification of cinnamyl alcohol **157** via oxidation of the hemiacetal embedded in the catalyst surface **158** to obtain the corresponding ester **44** in quantitative yield (Scheme 48B) [89].

**Scheme 49:**
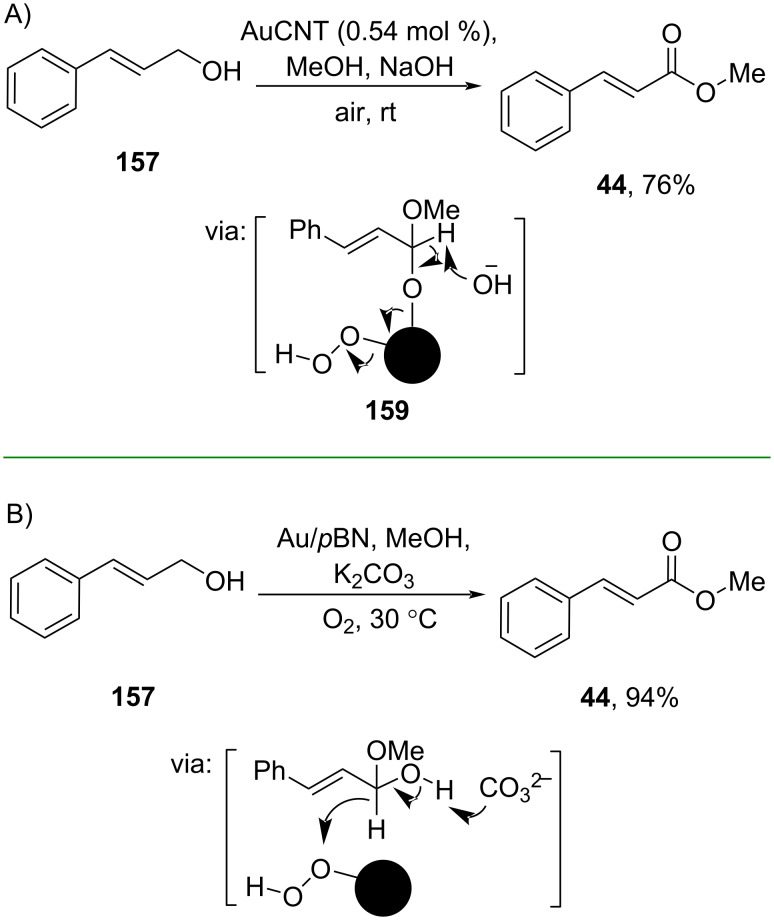
Au-supported on A) carbon nanotubes (CNT) and B) on porous boron nitride (*p*BN) as catalyst for the oxidative esterification of cinnamyl alcohol.

Furthermore, Doris and co-workers (2019) also utilized a Au catalyst for oxidative esterification reactions. Herein, Au nanoparticles supported on carbon nanotubes (AuCNT) catalyzed the conversion of cinnamyl alcohol **157** to the corresponding ester **44** via hemiacetal-anchored on the catalyst surface **159** using air as O_2_ source (Scheme 49A) [24]. On the other hand, Wang and co-workers (2019) employed a porous boron nitride (*p*BN)-supported Au catalyst (Au/*p*BN) to prepare the ester **44**, also via the formation of hemiacetal under O_2_ atmosphere (Scheme 49B) [25].

**Scheme 50:**
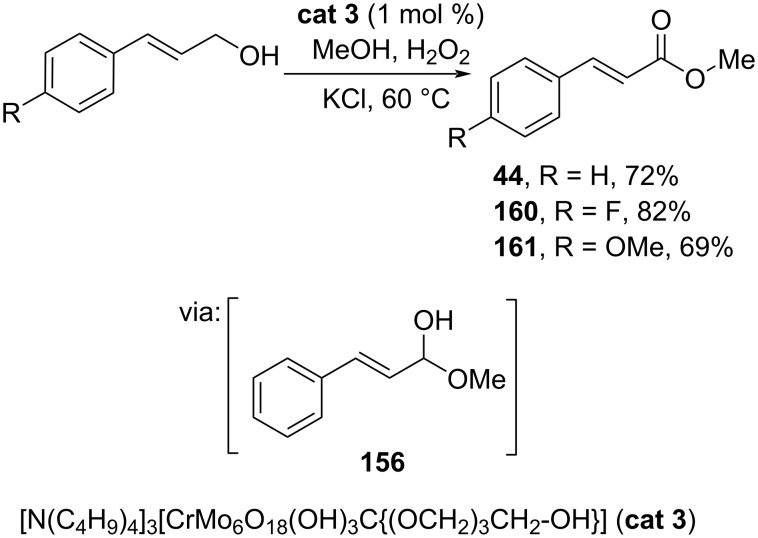
Cr-based catalyzed oxidative esterification of cinnamyl alcohols with H_2_O_2_ as the oxidant.

Wei and co-workers (2021) developed a Cr-based catalyst stabilized by a pentaerythritol-decorated Anderson-type polyoxometalate, [N(C_4_H_9_)_4_]_3_[CrMo_6_O_18_(OH)_3_C{(OCH_2_)_3_CH_2_-OH}] (**cat 3**), to catalyze the oxidative esterification of cinnamyl alcohols using H_2_O_2_. The reaction proceeds also via a hemiacetal intermediate **155** (Scheme 50) [90].

**Scheme 51:**
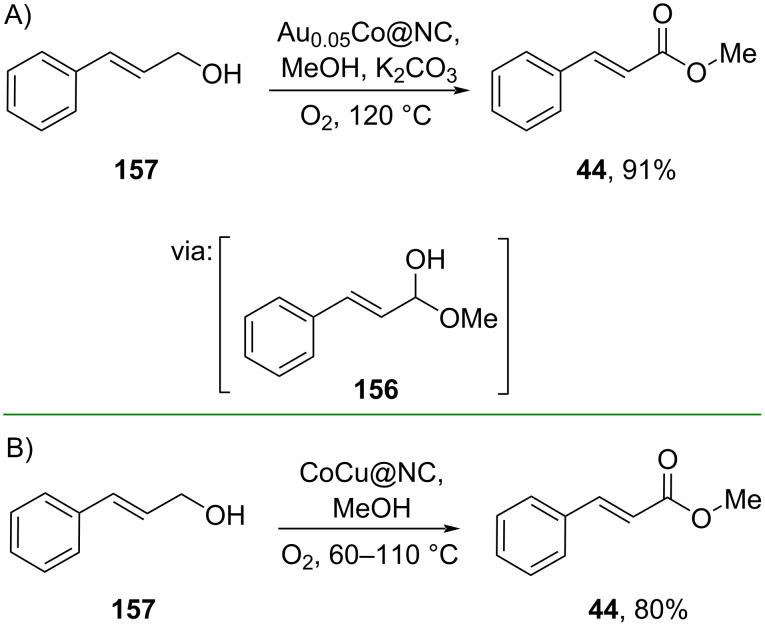
Co-based catalysts used for oxidative esterification of cinnamyl alcohol.

Utilizing earth-abundant transition metals, such as Fe, Cu, Ni, and Co, has been considered more sustainable due to their abundance. Several studies employing earth-abundant transition metals for oxidative esterifications of cinnamyl alcohols have flourished in recent years. For instance, Zheng and co-workers (2022) prepared the ester **44** via oxidative esterification of cinnamyl alcohol **157** catalyzed by an *N*-doped porous carbon-encapsulated Au-doped Co catalyst (Au_x_Co@NC) via the formation of hemiacetal intermediate **156** (Scheme 51A) [91]. Similarly, Leng and co-workers (2020) designed a Co/Cu nanoparticle-*co*-decorated nitrogen-doped carbon catalyst (CoCu@NC*_n_*) which was used to catalyze the oxidative esterification of cinnamyl alcohol (**157**) without the need of a base additive (Scheme 51B) [26].

**Scheme 52:**
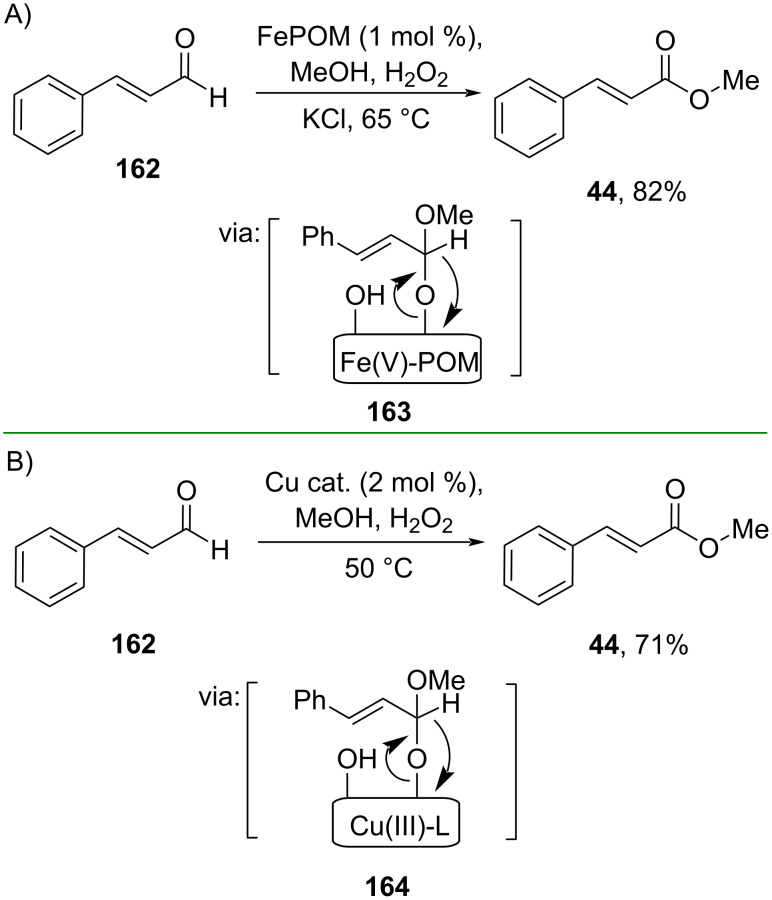
Iron (A) and copper (B)-catalyzed oxidative esterification of cinnamaldehyde.

**2.2.2 Aldehyde/ketone oxidation:** By employing non-precious transition metals, Wei and co-workers (2019) prepared a ring-like polyoxometalate (POM) inorganic ligand-supported Fe(III) catalyst (FePOM) to convert cinnamaldehyde (**162**) into methyl cinnamate (**44**) in good yield. The reaction proceeds through hemiacetal-attached on the catalyst surface **163** (Scheme 52A) [92]. Similarly, Erande and co-workers (2023) employed a Cu(II) square-planar complex {[Cu^II^L] LH_2_, 9,9’-(ethane-1,2-diylbis(azanediyl))bis(1*H*-phenalen-1-one} to convert cinnamaldehyde (**162**) into methyl cinnamate (**44**) in the presence of H_2_O_2_ as green oxidant via intermediate **164** formation (Scheme 52B) [93].

**Scheme 53:**
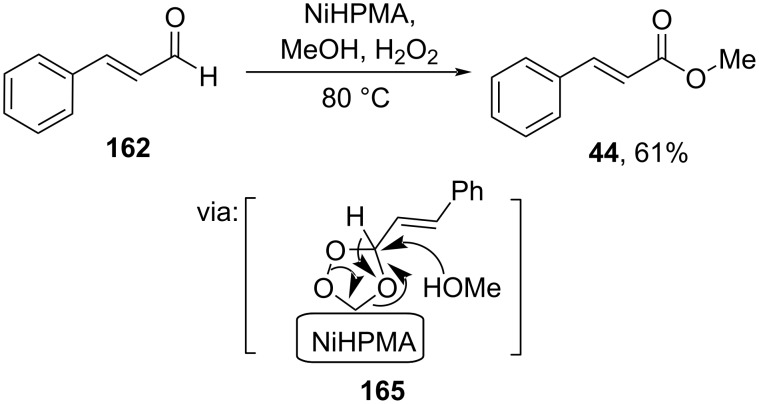
NiHPMA-catalyzed oxidative esterification of cinnamaldehyde.

Using a different earth-abundant transition metal, Patel and Patel (2020) utilized a Ni salt of phosphomolybdic acid (NiHPMA) to synthesize methyl cinnamate (**44**) from cinnamaldehyde (**162**) via a reactive peroxo species **165** (Scheme 53) [94].

**Scheme 54:**
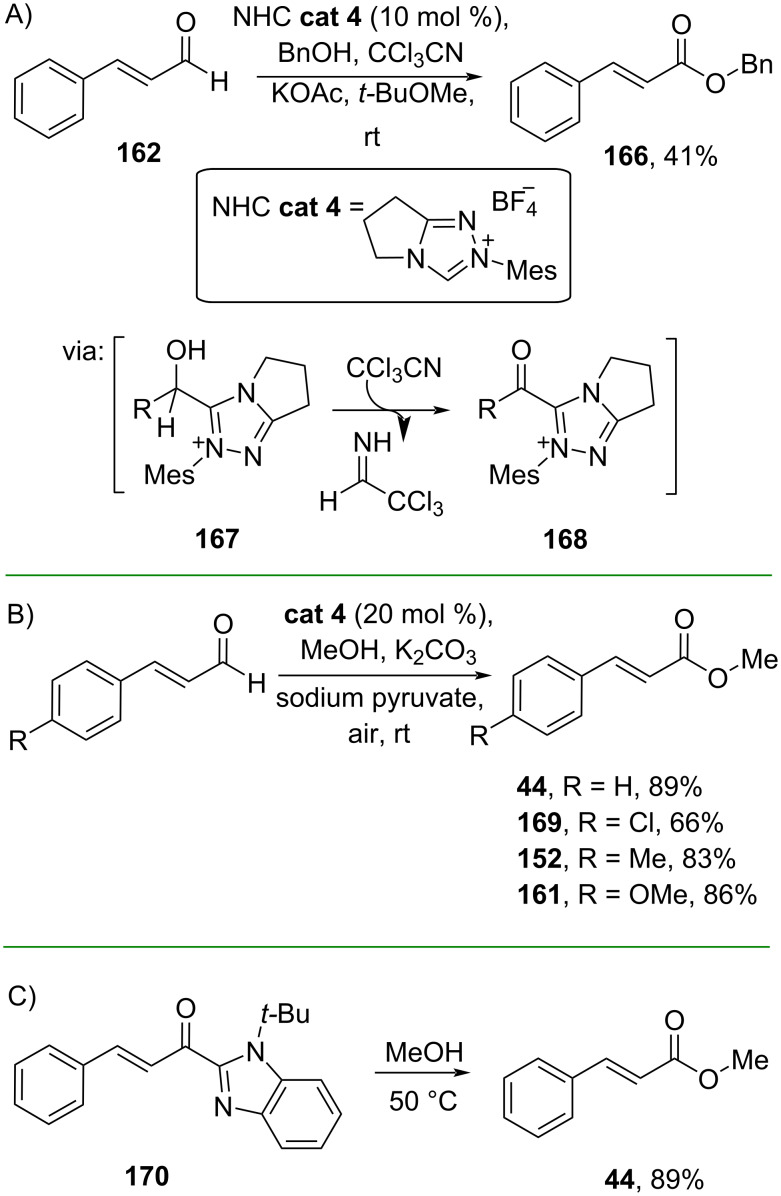
Synthesis of cinammic acid esters through NHC-catalyzed oxidative esterification via intermolecular oxidation.

Beyond transition metals, metal-free oxidative esterifications, such as carbene-based reactions, have also been explored, thus boosting sustainability value. For instance, Wang and co-workers (2019) synthesized benzyl cinnamate (**166**) from cinnamaldehyde (**162**) catalyzed by an NHC catalyst (**cat 4**) in the presence of the low-cost oxidant CCl_3_CN. The reaction involves formation of acyl azolium intermediate **168** formed through hydride transfer from **167** (Scheme 54A) [95]. Similarly, Huang and co-workers (2020) employed the same NHC catalyst but used ambient air as the external oxidant for acyl azolium intermediate formation (Scheme 54B) [96]. On the other hand, Ohshima and co-workers (2020) directly used acylimidazole **170** to prepare methyl cinnamate (**44**) without preactivation (Scheme 54C) [97].

**Scheme 55:**
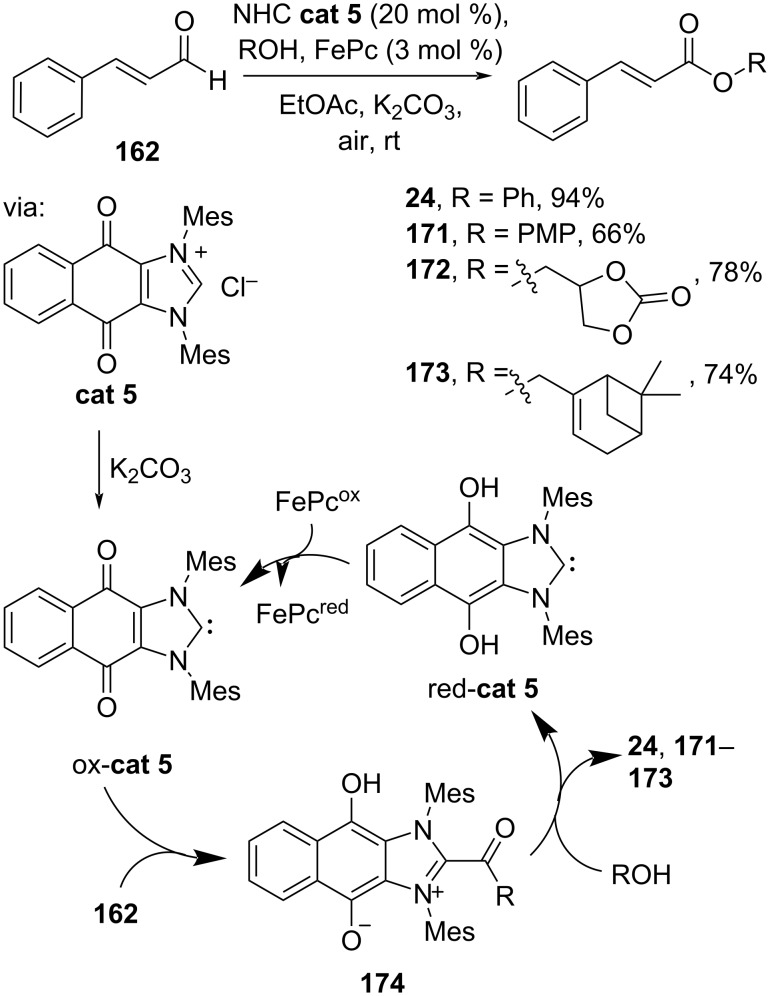
Redox-active NHC-catalyzed esterification via intramolecular oxidation.

Recently, Sundén and co-workers (2024) applied a hybrid NHC-based catalyst (**cat 5**) which, in its active form ox-**cat 5**, converts cinnamaldehyde (**162**) to the corresponding esters **24**, **171**–**173** via internal oxidation of azolium intermediate **174** under ambient air conditions (Scheme 55). The reduced catalyst red-**cat 5** is reoxidized by the co-catalyst Fe(III) phthalocyanine (FePc), closing the catalytic cycle [98].

**Scheme 56:**
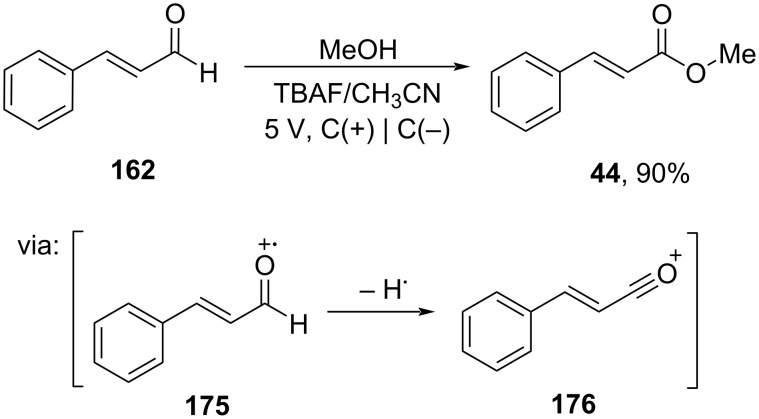
Electrochemical conversion of cinnamaldehyde to methyl cinnamate.

On the other hand, Syaikh and co-workers (2021) converted cinnamaldehyde (**162**) into methyl cinnamate (**44**) via an electrochemical method using TBAF as the supporting electrolyte (Scheme 56) [99]. Under these conditions, the aldehyde was oxidized to give an oxonium cation intermediate **176**.

**Scheme 57:**
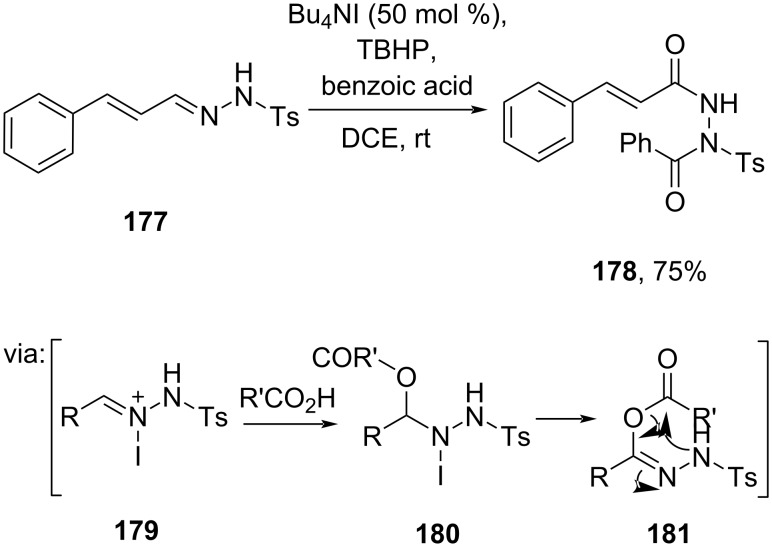
Bu_4_NI/TBHP-catalyzed synthesis of bisamides from cinnamalaldehyde *N*-tosylhydrazone.

Moreover, Babu and co-workers (2024) oxidized an imine, cinnamalaldehyde *N*-tosylhydrazone (**177**), by using TBHP and a catalytic amount of Bu_4_NI to synthesize bisamide **178**. The reaction proceeds through intermediates **179**–**181** (Scheme 57) [100]. In addition, the method has been successfully scaled up to a gram scale.

**Scheme 58:**
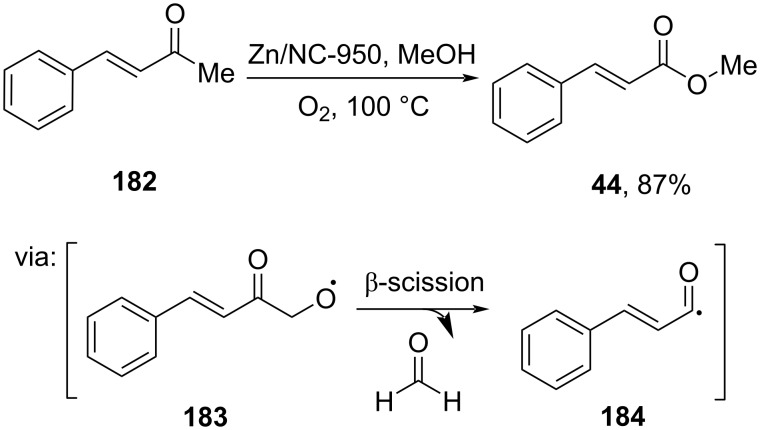
Zn/NC-950-catalyzed oxidative esterification of ketone **182**.

Han and co-workers (2021) converted ketone **182** into methyl cinnamate (**44**) catalyzed by Zn(II)-coordinated to microporous *N*-doped carbon (Zn/NC-950). the reaction proceeds through homolytic β-scission involving intermediates **183** and **184** (Scheme 58) [101].

#### 2.3 Alkenyl/alkynyl carboxylation

**Scheme 59:**
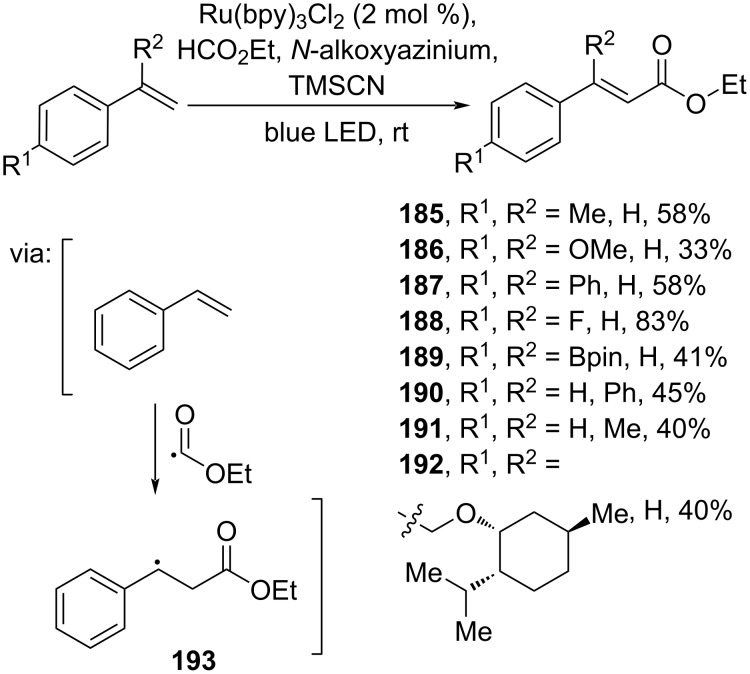
Ru-catalyzed oxidative carboxylation of terminal alkenes.

**2.3.1 Alkenyl carboxylation:** Li and co-workers (2021) investigated a photoinduced oxidative alkoxycarbonylation of styrenes with alkyl formates and the oxidant 4-cyano-1-(1-methylethoxy)pyridinium trifluoromethanesulfonate to give the corresponding cinnamate esters **185**–**192** via intermolecular addition of styrene to an alkoxyradical forming radical adduct **193** (Scheme 59) [102].

**Scheme 60:**
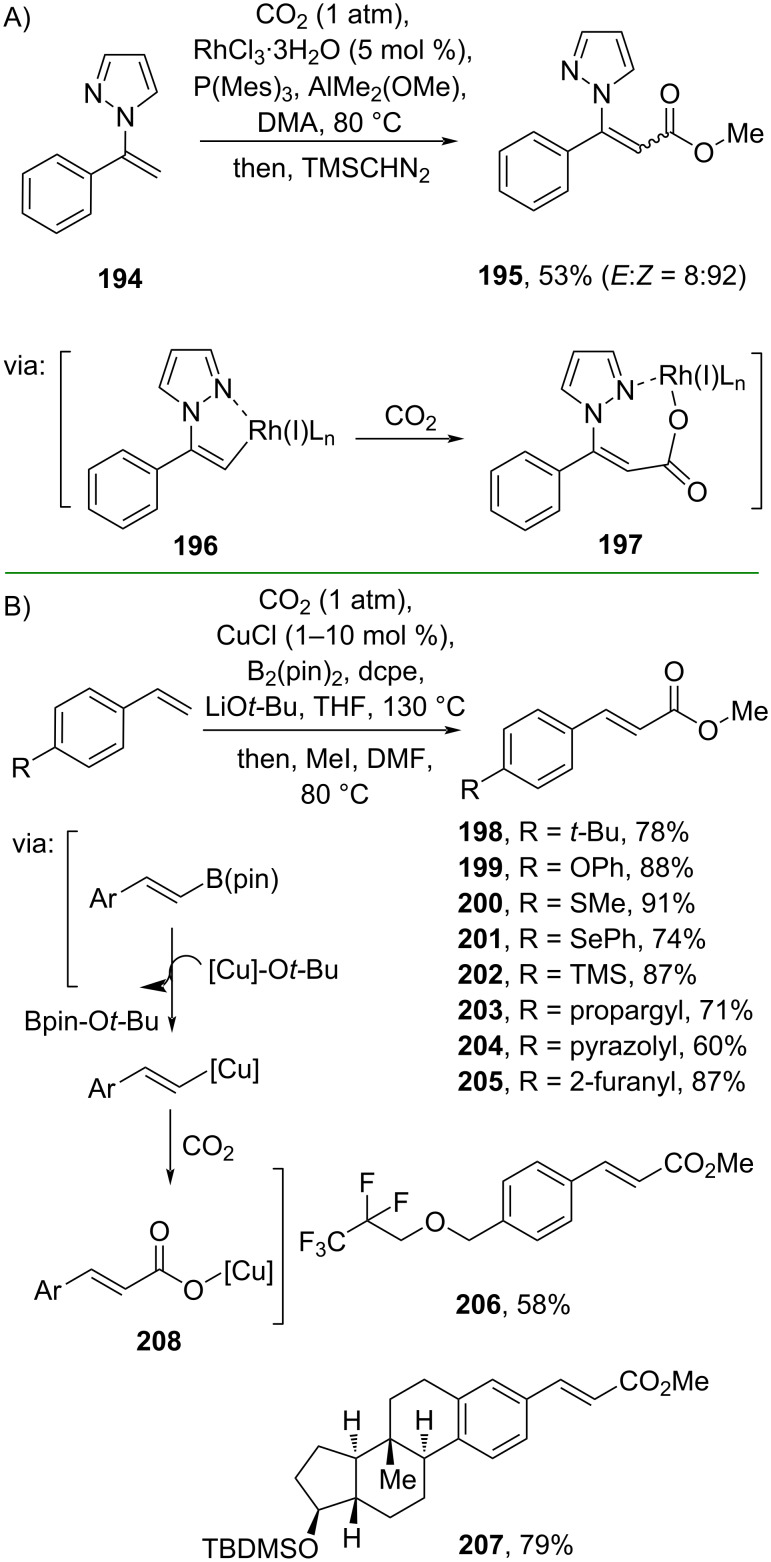
Direct carboxylation of alkenes using CO_2_.

Iwasawa and co-workers (2020) employed CO_2_ to perform the carboxylation of alkenylpyrazole **194** to the corresponding cinnamic ester **195** catalyzed by Rh(III) via a pyrazole-directed oxidative addition of the alkenyl C–H (**196**) accompanied by CO_2_ insertion to give Rh(I) carboxylate intermediate **197** (Scheme 60A) [103]. A gram scale operation has been successfully done for this method. Similarly, Hou and co-workers (2022) also used CO_2_ to carry out an auto-tandem Cu-catalyzed carboxylation of styrenes via β-hydride elimination (**208**) (Scheme 60B) [104]. Impressively, several natural product-like compounds (e.g., **207**) were successfully prepared using this method.

**Scheme 61:**
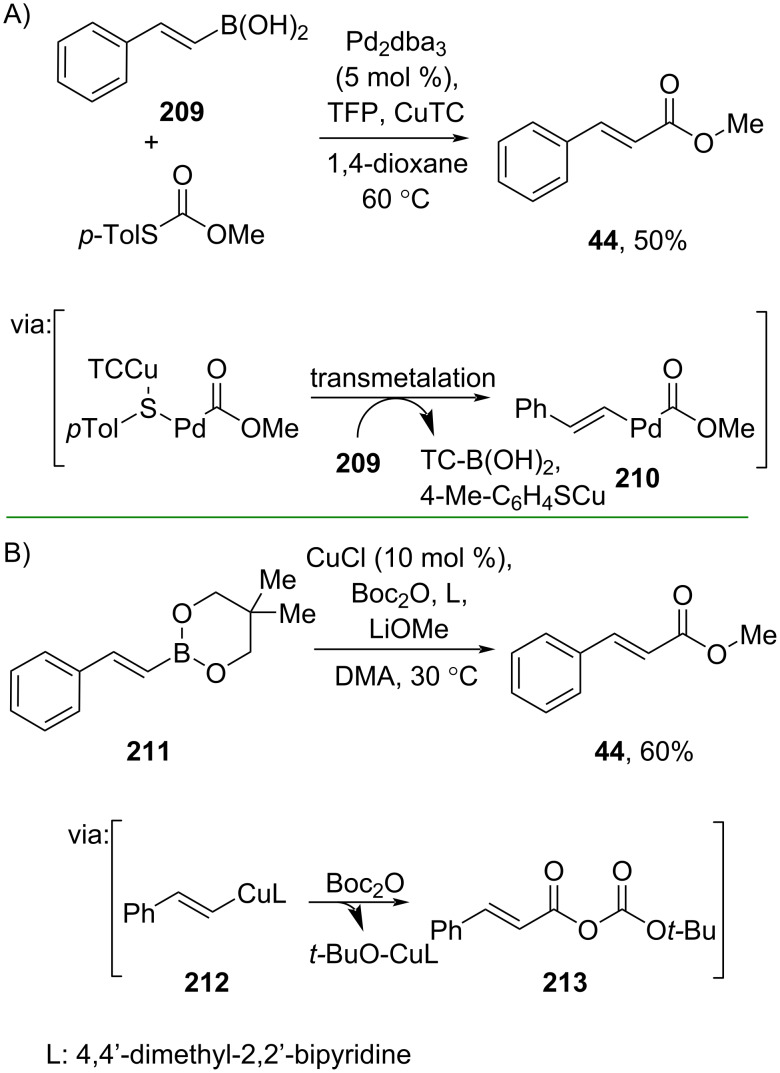
Carboxylation of alkenylboronic acid/ester.

Furthermore, Dai and co-workers (2020) employed alkenylboronic acid **209** and *O*-methyl *S*-*p*-tolyl thiocarbonate to prepare methyl cinnamate (**44**) catalyzed by Pd_2_dba_3_ in the presence of Cu(I) thiophene-2-carboxylate (CuTC) and the ligand tri(2-furyl)phosphine (TFP). The reaction proceeds via oxidative Pd insertion into the Cu-activated thioester followed by transmetalation with alkenylboronic acid to give complex **210** (Scheme 61A) [105]. The method has been scaled up to a gram scale. Similarly, Hu and co-workers (2021) utilized alkenylboronic ester **211** and Boc_2_O to synthesize methyl cinnamate (**44**). The reaction is catalyzed by Cu(I) and 4,4’-dimethyl-2,2’-bipyridine as the ligand and proceeds via organocopper intermediate **212** followed by carbonate **213** formation (Scheme 61B) [106]. In addition, a gram scale reaction has been smoothly conducted.

**Scheme 62:**
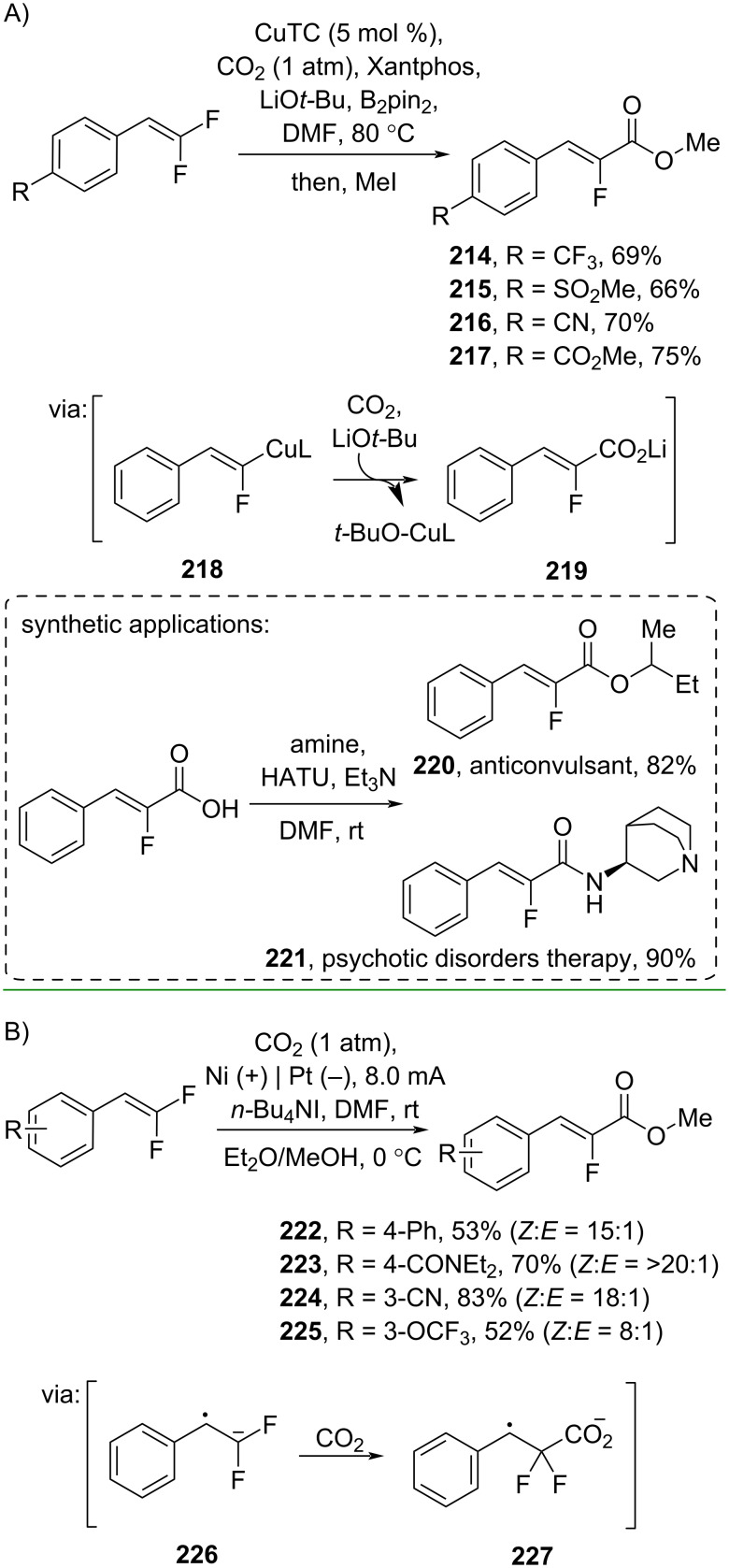
Carboxylation of *gem*-difluoroalkenes with CO_2_.

Yu and co-workers (2019) reported a Cu-catalyzed carboxylation of *gem*-difluoroalkenes with CO_2_ to give the corresponding α-fluoro methyl cinnamates **214–217** via transmetallation and carboxylation (**219** and **218**) (Scheme 62A) [107]. The thus-obtained α-fluorocinnamic acid was successfully converted into the bioactive compounds **220** and **221**. In addition, the reaction capacity could be increased to a gram-scale operation. Also utilizing *gem*-difluoroalkenes, Zhou and co-workers (2020) performed a direct electrochemical carboxylation with CO_2_ to the corresponding α-fluoro methyl cinnamates **222–225** via formation of radical anion **226** followed by carboxylation (**227**) (Scheme 62B) [108].

**Scheme 63:**
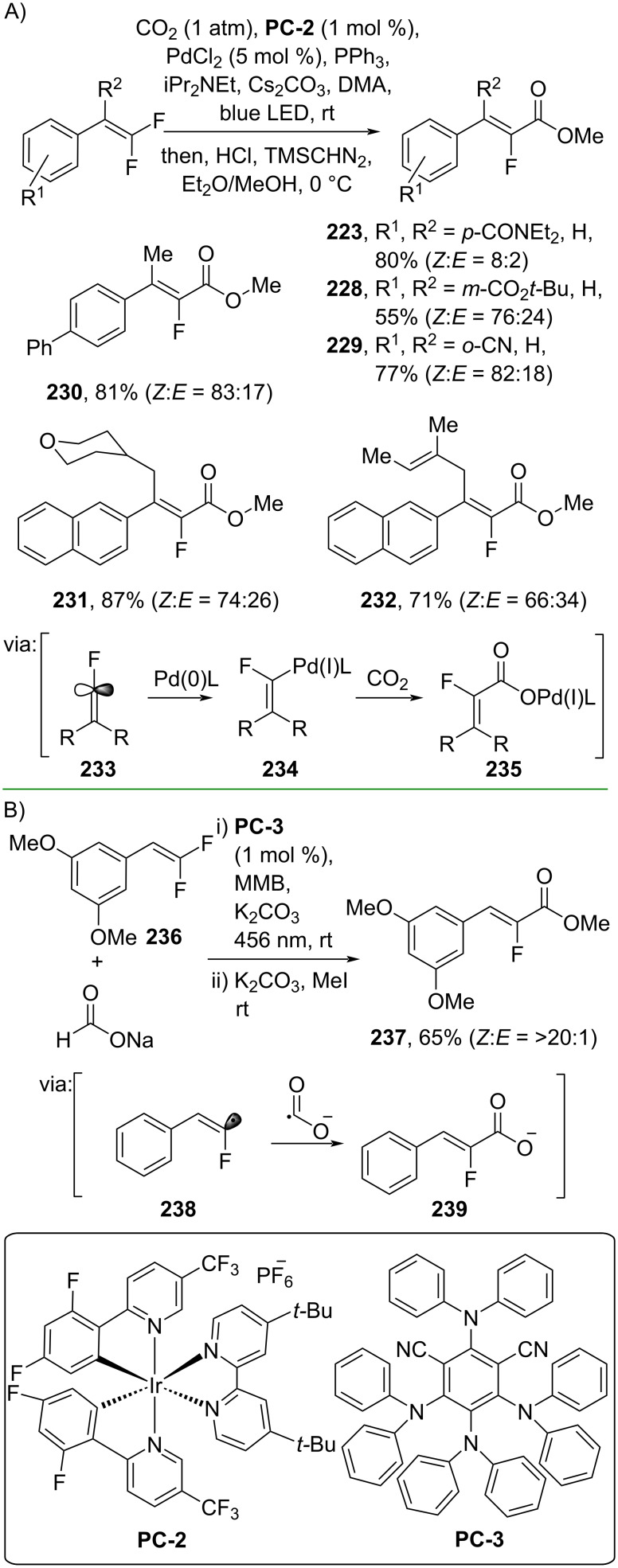
Photoredox-catalyzed carboxylation of difluoroalkenes.

Moreover, Feng and co-workers (2019) employed *gem*-difluoroalkenes and CO_2_ to perform a photoredox/Pd dual-catalyzed carboxylation reaction affording the corresponding methyl cinnamates **223**, **228–232**. The reaction proceeds via photochemical-induced formation of fluoroalkenyl radical **233**, followed by Pd insertion (**234**) and carboxylation (**235**) (Scheme 63A) [109]. Recently, Wang and co-workers (2024) reported a photoredox-promoted carboxylation of *gem*-difluoroalkene **236** by using formate salts, which also involves formation of a fluoroalkenyl radical intermediate **238** (Scheme 63B) [110].

**Scheme 64:**
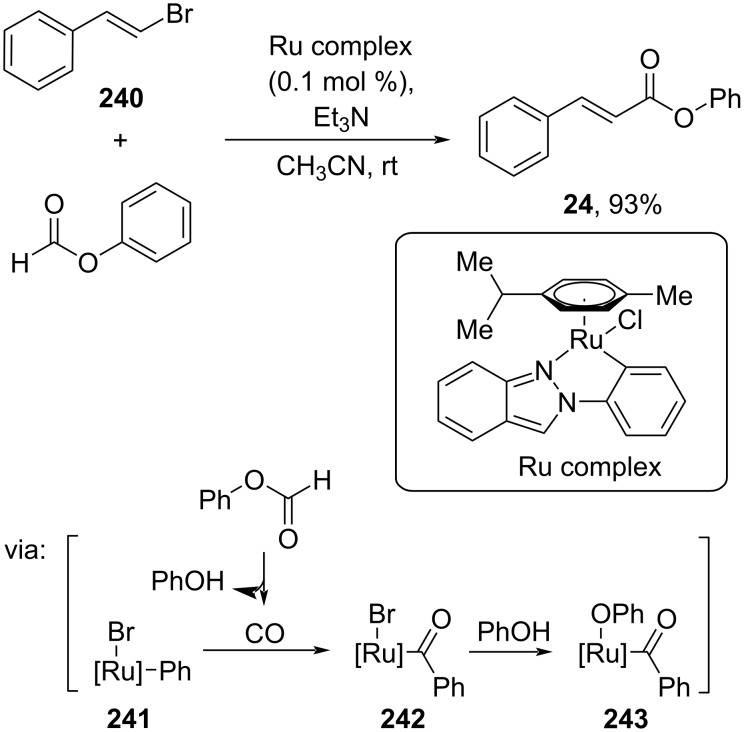
Ru-catalyzed carboxylation of alkenyl halide.

On the other hand, Yao and co-workers (2023) used alkenyl halides **240** and formate salts to prepare cinnamic ester **24** catalyzed by a Ru complex via an oxidative addition/reductive elimination cycle involving intermediates **241**–**243** (Scheme 64) [111]. The method has been successfully scaled up to a gram scale.

**Scheme 65:**
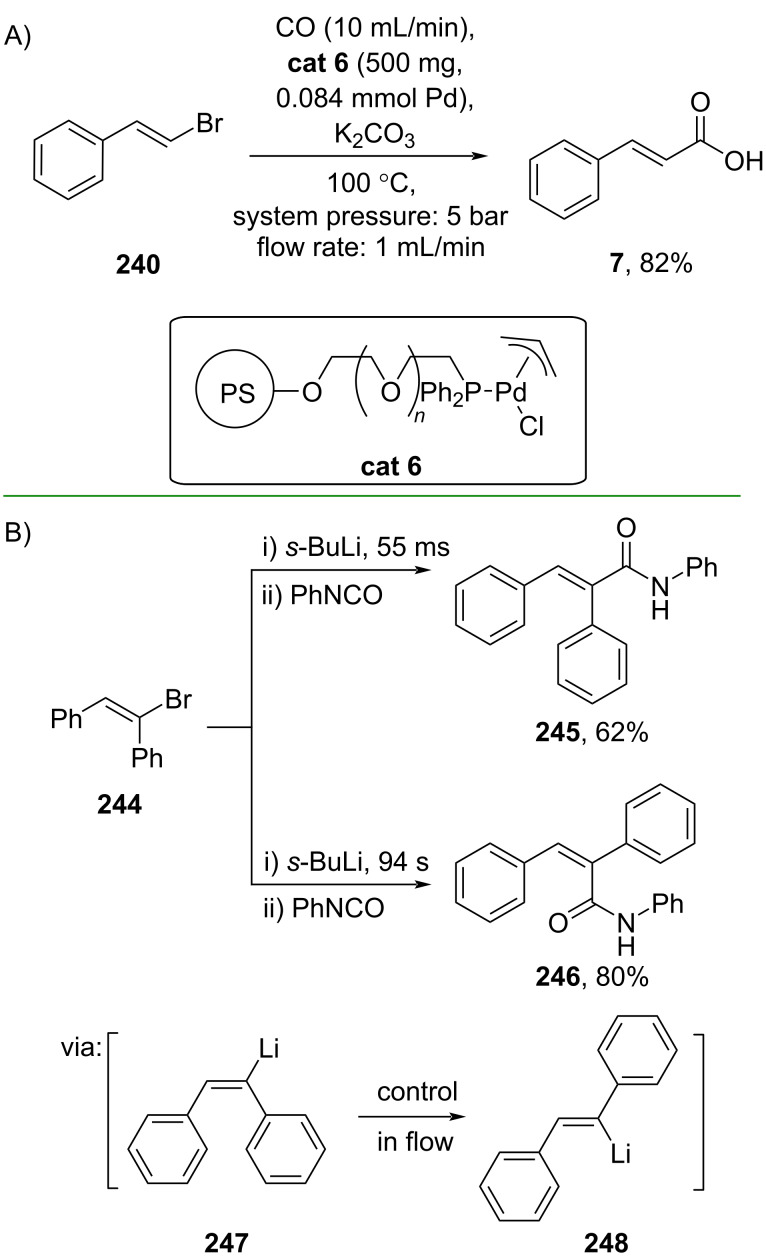
Carboxylation of alkenyl halides under flow conditions.

Uozumi and co-workers (2019) reported a carbonylation under aqueous flow conditions using alkenyl halide **240** to prepare cinnamic acid (**7**) catalyzed by an amphiphilic polystyrene-poly(ethylene glycol) resin-supported Pd-diphenylphosphine catalyst (**cat 6**) (Scheme 65A) [31]. Also using flow conditions, Kim and co-workers (2021) reported the *cis*–*trans* isomerization of α-functionalized stilbenes in a flow microreactor (Scheme 65B) [112]. The isomerization could be regioselectively controlled in an incredibly short time within milliseconds (**247**, **248**) to give either the *E*-isomeric product **245** or the corresponding *Z*-isomeric product **246**, both in good yields.

**Scheme 66:**
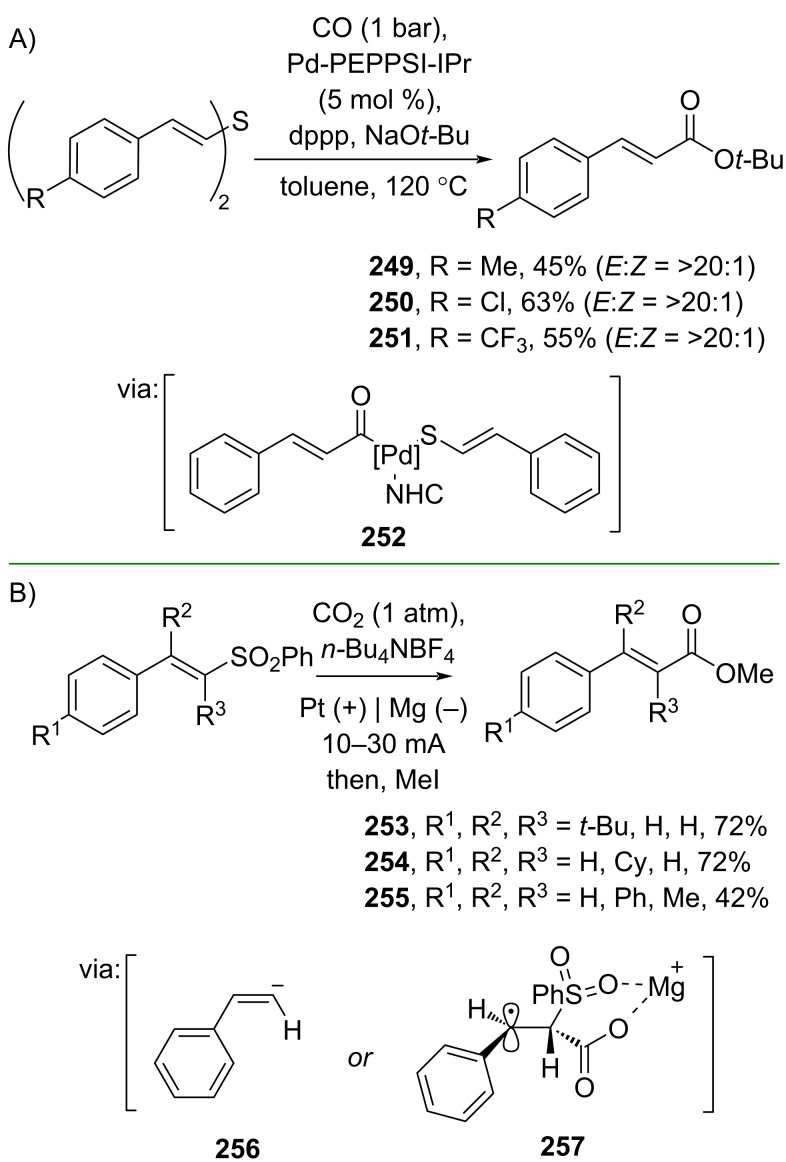
Cinnamic acid ester syntheses through carboxylation of alkenyl sulfides/sulfones.

Alkenyl sulfides have also been used for the preparation of cinnamic acid esters. For example, Wu and co-workers (2020) studied the Pd-catalyzed carbonylation of alkenyl sulfides in the presence of NHC ligands via C–S cleavage (**252**) to afford the corresponding cinnamic esters **249–251** (Scheme 66A) [113]. Similarly, Chen and co-workers (2023) utilized alkenyl sulfones and CO_2_ to synthesize cinnamic acid ester **253–255** via an electrochemical set-up by generating the reactive intermediate **256** or **257** (Scheme 66B) [114].

**Scheme 67:**
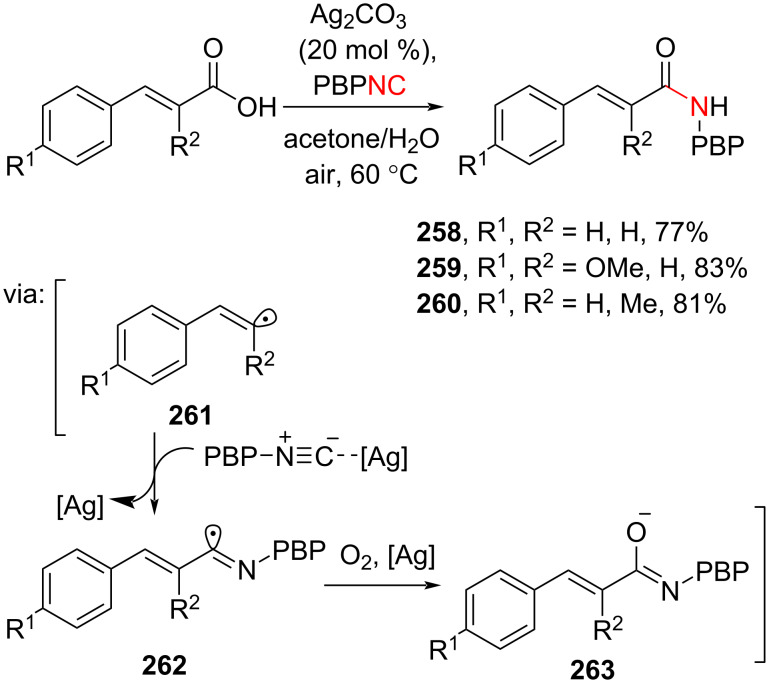
Cinnamic acid derivatives synthesis through a Ag-catalyzed decarboxylative cross-coupling proceeding via a radical mechanism.

An interesting transformation of cinnamic acid to its derivatives can be achieved through decarboxylative cross-coupling. Recently, Wang and co-workers (2024) reported the Ag-catalyzed decarboxylative cross-coupling of cinnamic acids with isocyanide to give the corresponding amides **258–260**. The reaction involves a vinylic radical intermediate **261** (Scheme 67) [115].

**Scheme 68:**
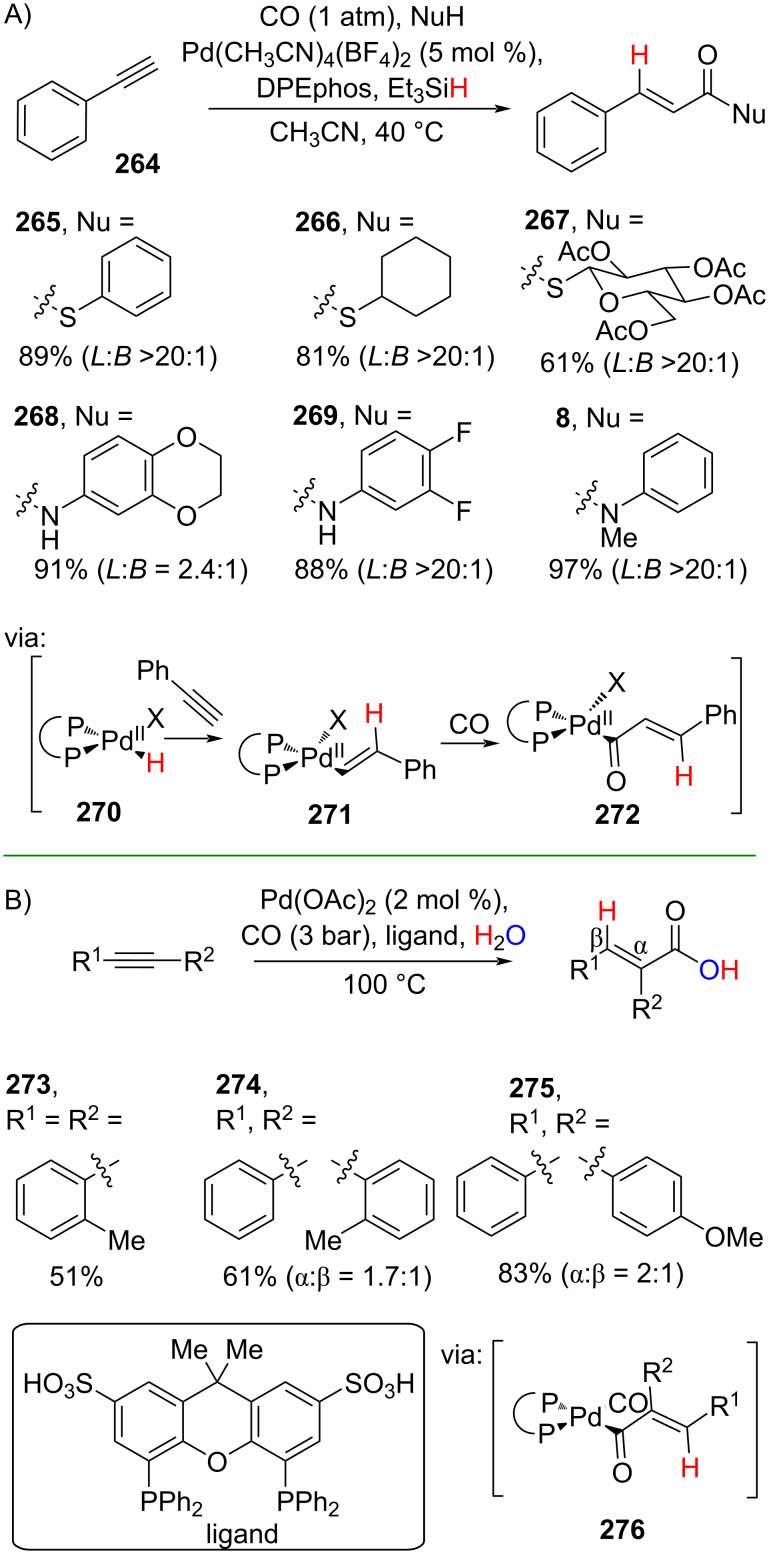
Pd-catalyzed alkyne hydrocarbonylation.

**2.3.2 Alkynyl carboxylation:** Zhu and co-workers (2023) reported a Pd-catalyzed hydrocarbonylation of phenylacetylene (**264**) using CO and hydrosilane as the hydrogen source to prepare cinnamamides **8**, **265–269** in good yields with high linear-to-branched (L/B) selectivity of >20:1. The reaction involves the formation of palladium hydride (Pd–H) **270** as the key species followed by alkyne and CO insertion reactions via **271** and **272** (Scheme 68A) [116]. On the one hand, Jia and co-workers (2021) utilized environmentally benign water as the hydrogen source to perform the Pd-catalyzed alkyne hydrocarbonylation with CO via **276** to obtain the corresponding cinnamic acids **273–275** in good yields (Scheme 68B) [117]. In addition, a gram scale operation has been carried out.

**Scheme 69:**
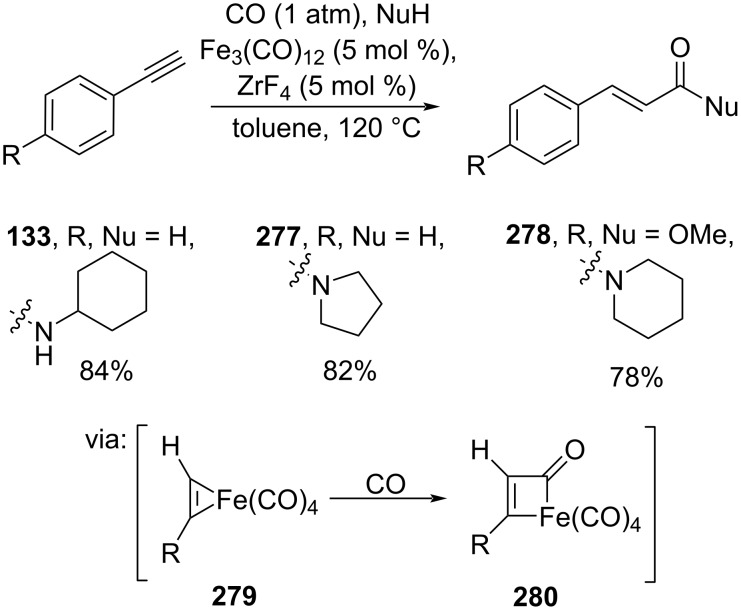
Fe-catalyzed alkyne hydrocarbonylation.

Li and co-workers (2019) employed a non-precious transition metal, ligand-free Fe_3_(CO)_12_, to catalyze the alkyne hydrocarbonylation using CO and ZrF_4_ as the co-catalyst. The reaction afforded the corresponding amides **133**, **277**, and **278** in good yields via acyl carbonyl iron intermediate **280**, which was observed by NMR (Scheme 69) [118].

**Scheme 70:**
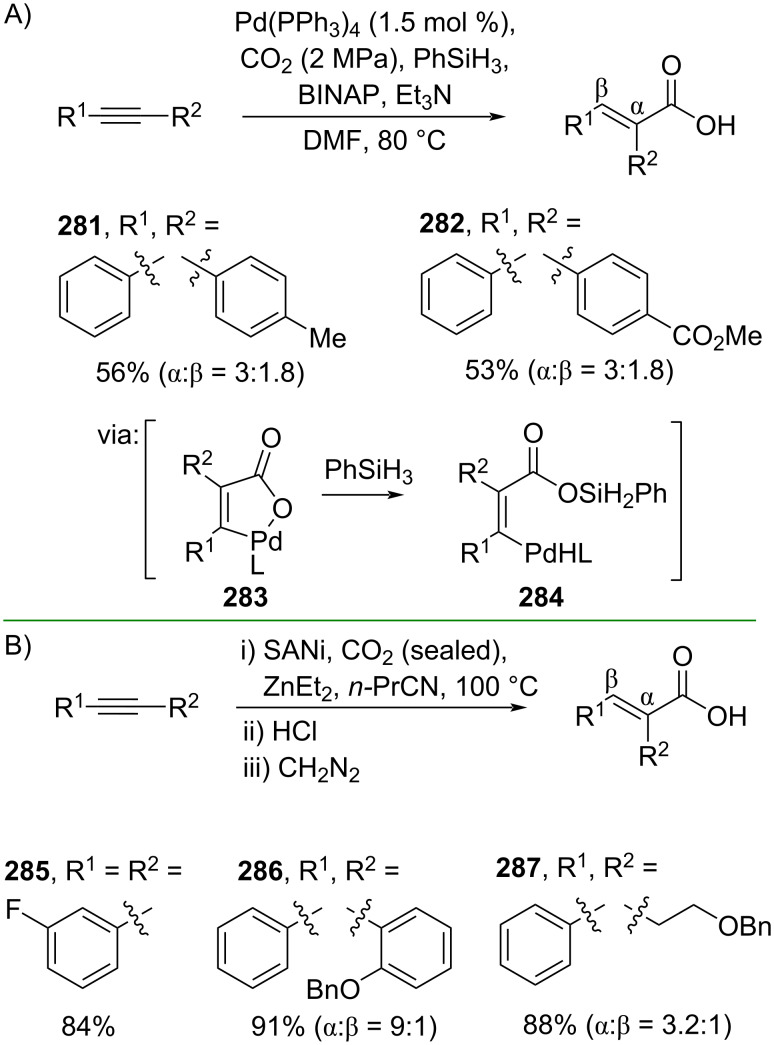
Alkyne hydrocarboxylation using CO_2_.

By applying less toxic and safer CO_2_ instead of CO, Jiang and co-workers (2020) reported a Pd-catalyzed hydrocarboxylation of alkynes with high regioselectivity to obtain the corresponding cinnamic acids **281** and **282** in good yields via cyclopalladation intermediate **283** (Scheme 70A) [119]. The method has been scaled up to a gram scale operation. Furthermore, Sato and co-workers (2019) employed an air-stable Ni nanoparticle supported on sulfur-modified gold (SANi) to convert alkynes to the corresponding cinnamic acids **285**–**287** (Scheme 70B) [120]. In addition, the SANi catalyst could be recycled without significant loss of activity.

**Scheme 71:**
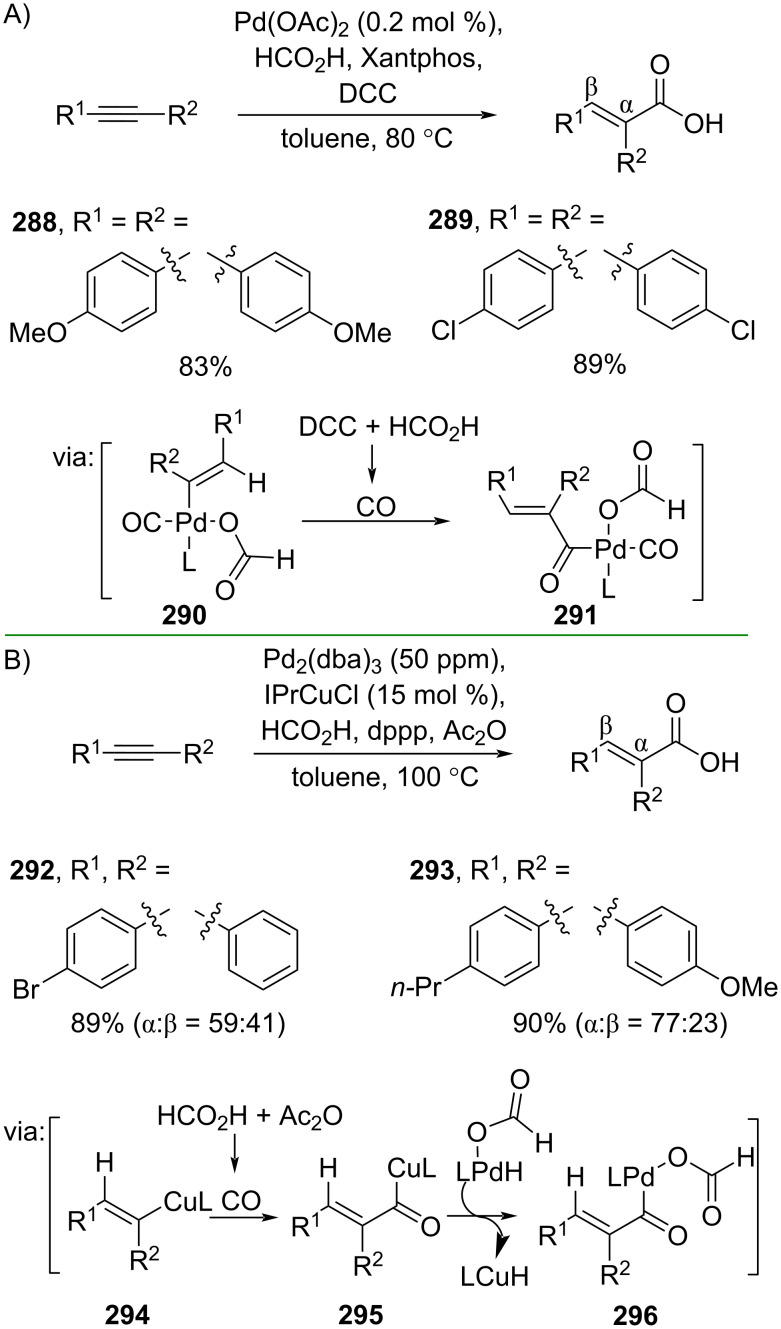
Alkyne hydrocarboxylation using HCO_2_H as CO surrogate.

By generating CO in situ from HCO_2_H, He and co-workers (2022) developed a Pd-catalyzed hydrocarboxylation of alkynes to the corresponding cinnamic acids **288** and **289** in good yields (Scheme 71A) [121]. Mechanistically, HCO_2_H protonates the Pd-coordinated alkyne to give the alkenyl–Pd intermediate **290**, accompanied by CO insertion to afford the acylpalladium intermediate **291**. The same group (2021) also developed a Cu/Pd dual catalysis method to carry out the alkyne hydrocarboxylation using HCO_2_H as the CO surrogate to give the corresponding cinnamic acids **292** and **293** via the generation of vinylcopper species **294** followed by CO insertion (**295**) and transmetalation with palladium hydride to afford the acylpalladium species **296** (Scheme 71B) [122].

**Scheme 72:**
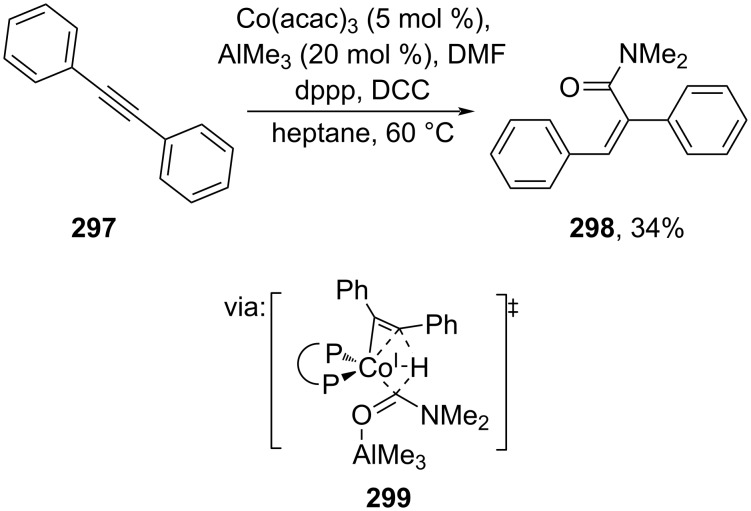
Co/AlMe_3_-catalyzed alkyne hydrocarboxylation using DMF.

Utilizing a non-precious transition metal as an alternative for Pd, Yoshikai and co-workers (2020) reported the cooperative cobalt/Lewis acidic AlMe_3_-catalyzed hydrocarboxylation of alkynes **297** with *N*,*N’*-dimethylformamide (DMF) to afford the corresponding cinnamamide **298** via ligand-to-ligand hydrogen transfer **299** (Scheme 72) [123].

**Scheme 73:**
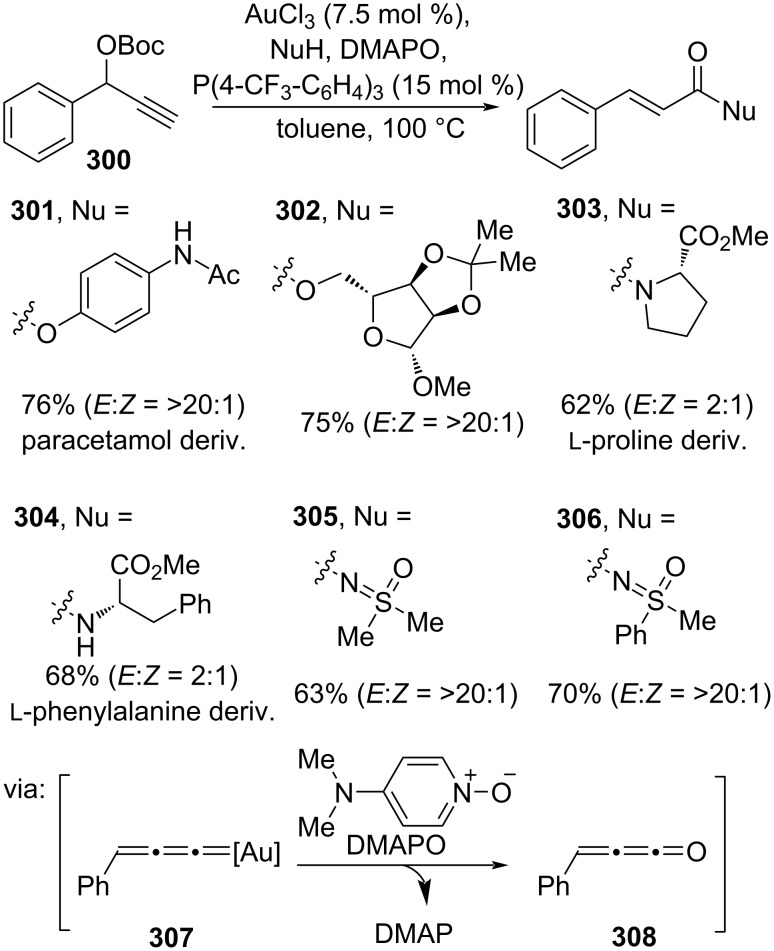
Au-catalyzed oxidation of Au–allenylidenes.

Furthermore, Song and co-workers (2023) employed propargylic ester **300** to prepare the corresponding cinnamic esters/amides with excellent anti-Markovnikov selectivity catalyzed by Au(III) in the presence of dimethylaminopyridine *N*-oxide (DMAPO) as oxidant. The reaction proceeds via Au–allenylidene species **307** (Scheme 73) [124]. Various cinnamic esters and amides with natural product-based alkoxy groups **301**–**304** and sulfoximines **305** and **306**, a relatively new prima donna of functional groups explored in drug design, were smoothly prepared.

#### 2.4 Miscellaneous reactions

**Scheme 74:**
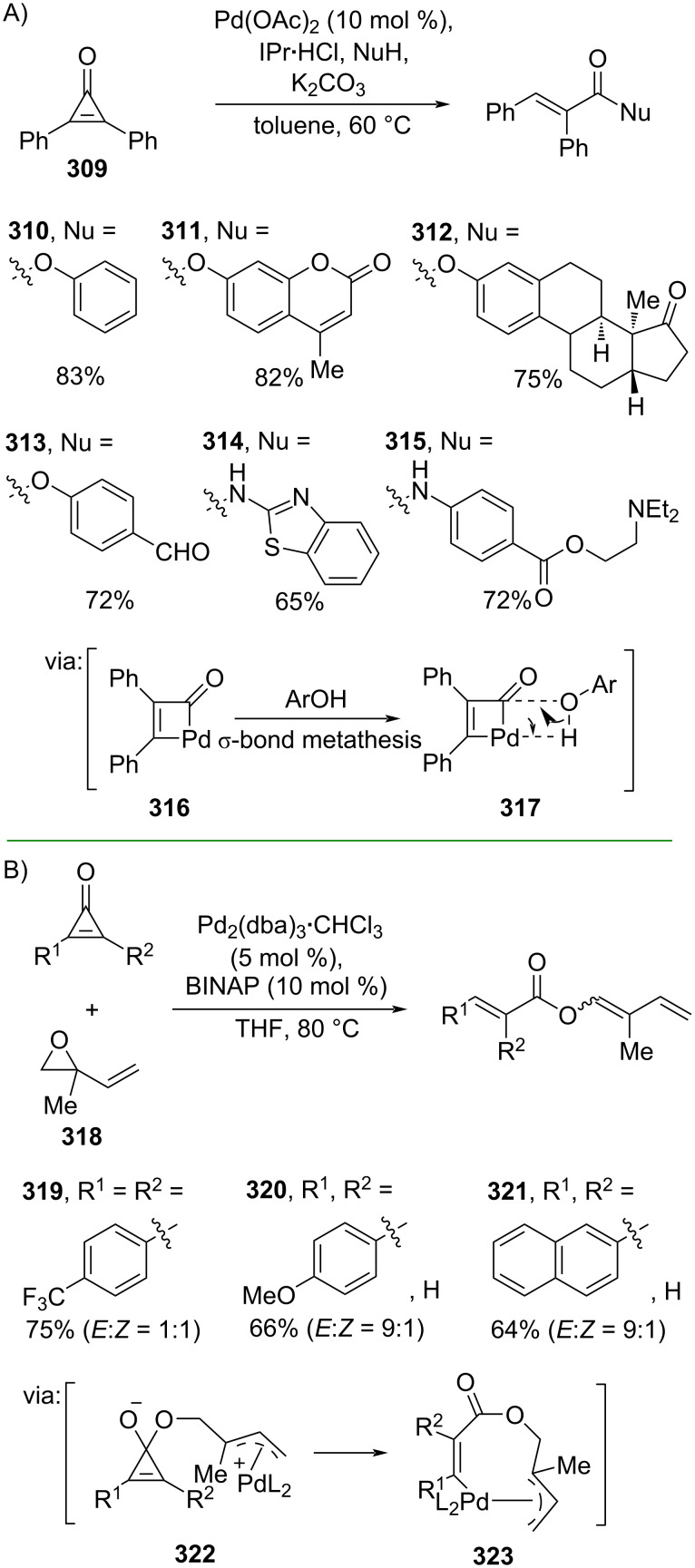
Pd-catalyzed C–C-bond activation of cyclopropenones to synthesize unsaturated esters and amides.

Cyclopropenone, the smallest Hückel aromatic system, has been subjected to the ring opening reactions, driven by the release of ring strain. Numerous transformations of cyclopropenone based on C–C bond activations have been explored. For instance, Ravikumar and co-workers (2021) employed diphenylcyclopropenone (**309**) to synthesize the corresponding esters **310**–**313** and amides **314** and **315**. The reaction was catalyzed by Pd/*N*-heterocyclic carbene via oxidative Pd insertion into the C–C bond of cyclopropenone to give a cyclic intermediate **316** (Scheme 74A) [125]. On the other hand, Wu and co-workers (2022) developed a Pd-catalyzed selective ring-opening of cyclopropenones and vinyl epoxide **318** to give the corresponding esters **319**–**321** in good yields via a π–allyl palladium intermediate **323** (Scheme 74B) [126].

**Scheme 75:**
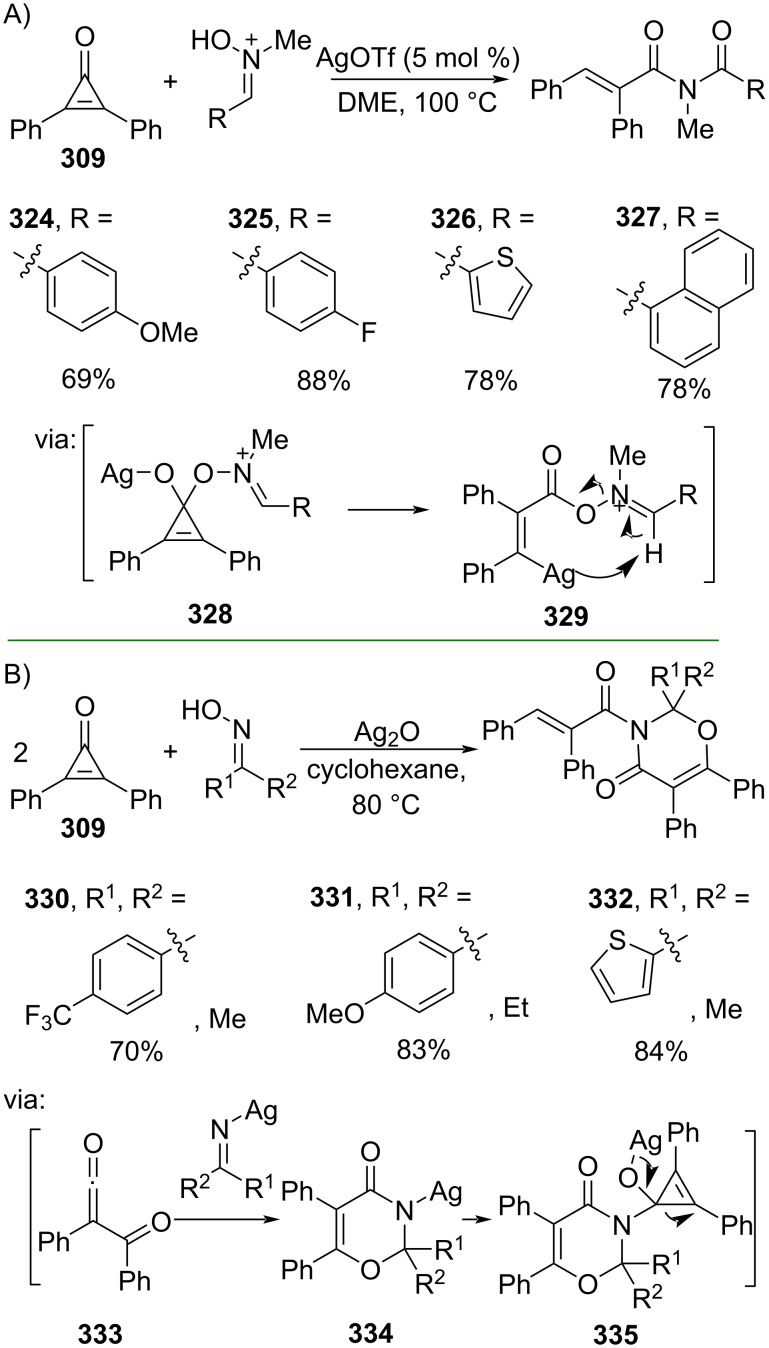
Ag-catalyzed C–C-bond activation of diphenylcyclopropenone.

In addition, Sun and co-workers (2020) utilized diphenylcyclopropenone (**309**) and nitrones to access the corresponding imides **324–327** via a Ag-mediated β-carbon elimination promoted ring-opening reaction involving species **328** and **329** (Scheme 75A) [127]. The method could be scaled up to a gram scale operation. Similarly, Liu and co-workers (2020) reported a Ag_2_O-catalyzed ring-opening of cylopropenone **309** with oximes to give the corresponding series of novel 1,3-oxazinones **330–332** in good yields. The reaction involves a [4 + 2] cycloaddition (**334**) of the fragmented cyclopropenone **333** followed by the second cyclopropenone ring-opening (**335**) (Scheme 75B) [128].

**Scheme 76:**
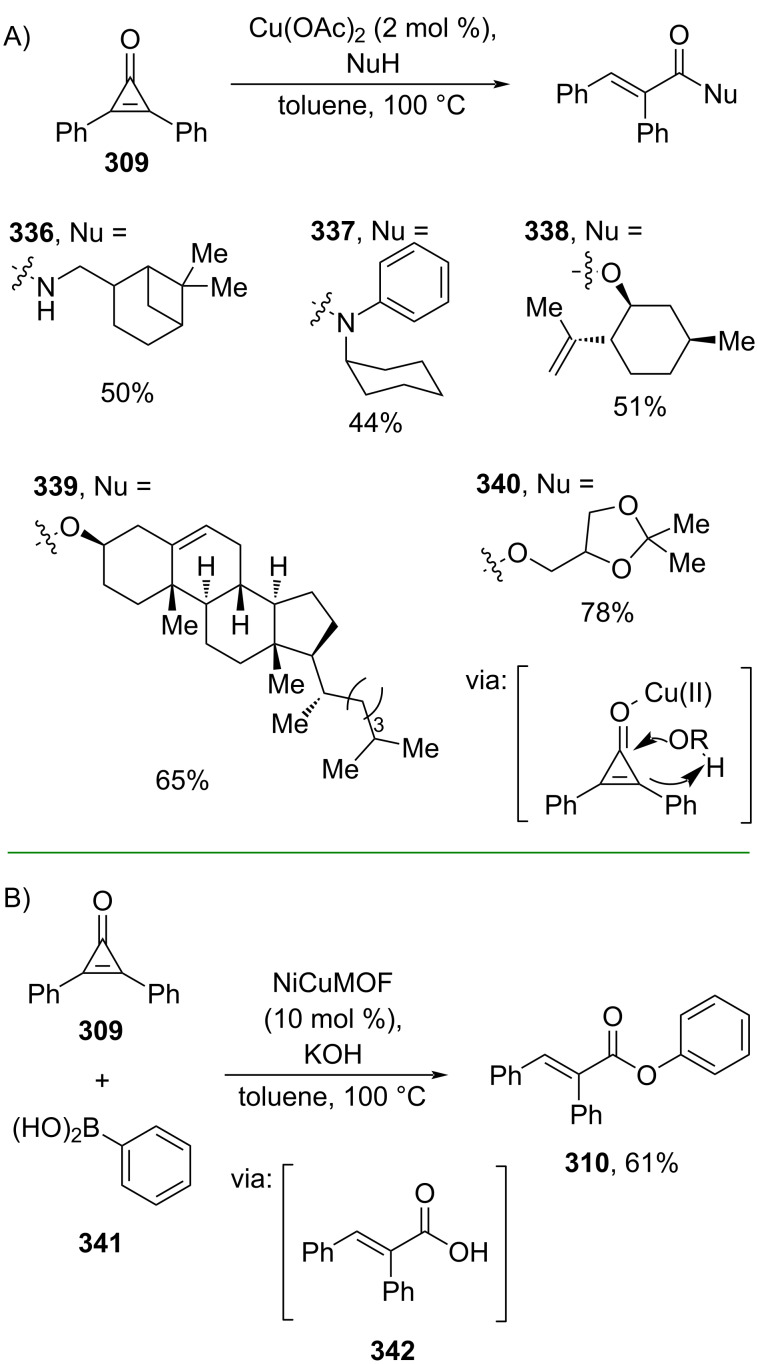
Cu-catalyzed C–C bond activation of diphenylcyclopropenone.

Leyva-Pérez and co-workers (2022) reported the esterification and amidation of diphenylcyclopropenone (**309**) with alcohols and amines, respectively, catalyzed by Cu(II) (Scheme 76A) [129]. In addition, they prepared a multimetal-organic framework (M-MOF, M= Cu, Ni) which successfully catalyzed the one-pot cyclopropenone hydration/Chan–Lam coupling reaction of **309** and boronic acid **341** to give the corresponding ester **310** in good yield via the formation of acid **342** (Scheme 76B).

**Scheme 77:**
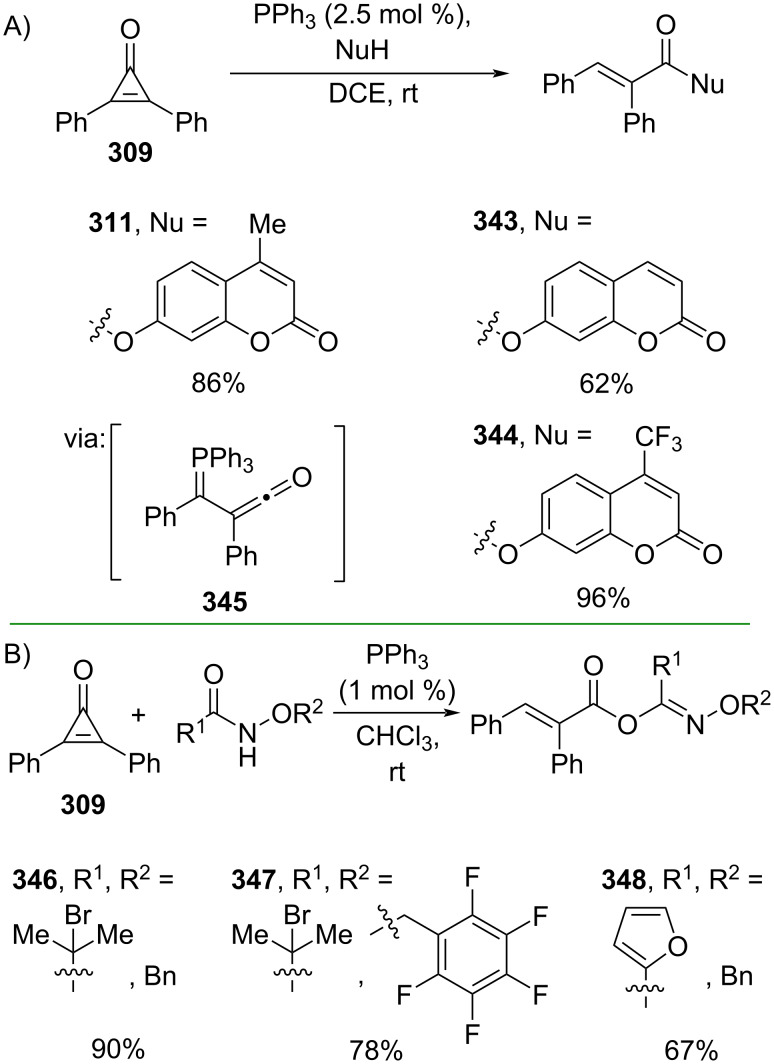
PPh_3_-catalyzed C–C-bond activation of diphenylcyclopropenone.

Cyclopropenone was also subjected to non-metal-catalyzed ring-opening reactions, e.g., by using PPh_3_. For instance, Reddy and co-workers (2024) utilized PPh_3_ to catalyze the esterification of diphenylcyclopropenone (**309**) with a series of natural products of the coumarin family to give the corresponding esters **311**, **343**, and **344** in good yields via the formation of an active α-ketenyl phosphorus ylide **345** (Scheme 77A) [130]. Similarly, Lin and co-workers (2024) employed PPh_3_ to selectively catalyze the esterification reaction of cyclopropenone **309** and amides to give the corresponding esters **346–348** containing an oxime ether in good yields (Scheme 77B) [131]. In addition, a gram scale operation has been successfully demonstrated.

**Scheme 78:**
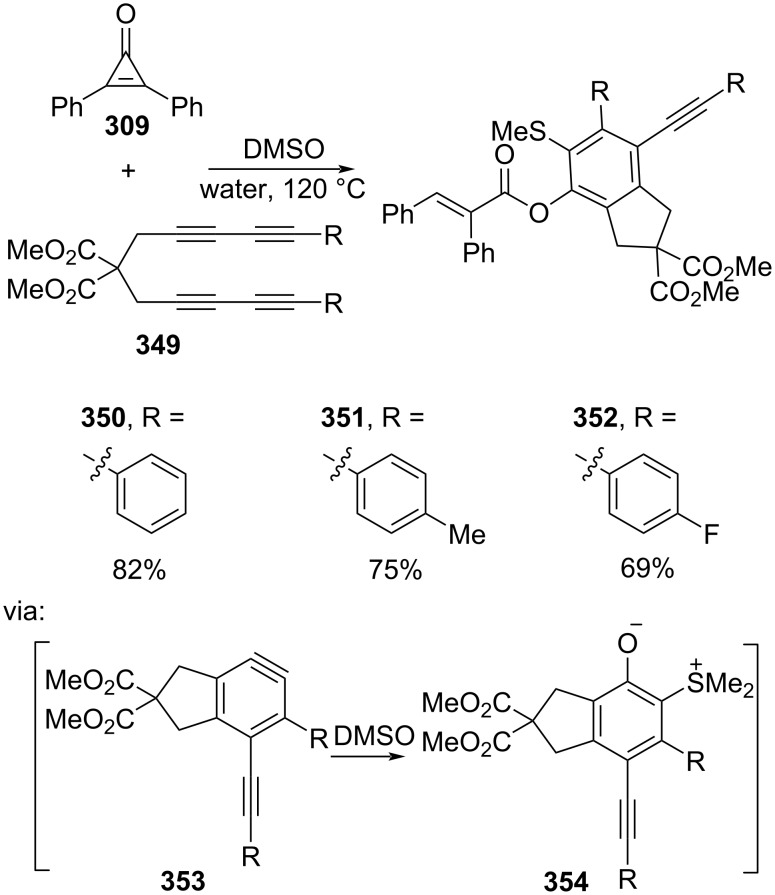
Catalyst-free C–C-bond activation of diphenylcyclopropenone.

In addition, Hu and co-workers (2020) studied a catalyst-free multicomponent coupling reaction of benzynes derived from alkyne **349**, cyclopropenone **309**, and sulfoxides to give the corresponding *o*-(methylthio)phenyl acrylates **350–352** in good yields via benzyne intermediate **353** followed by a [2 + 2] cycloaddition with the sulfoxide affording an *o*-quinone intermediate **354** (Scheme 78) [132].

**Scheme 79:**
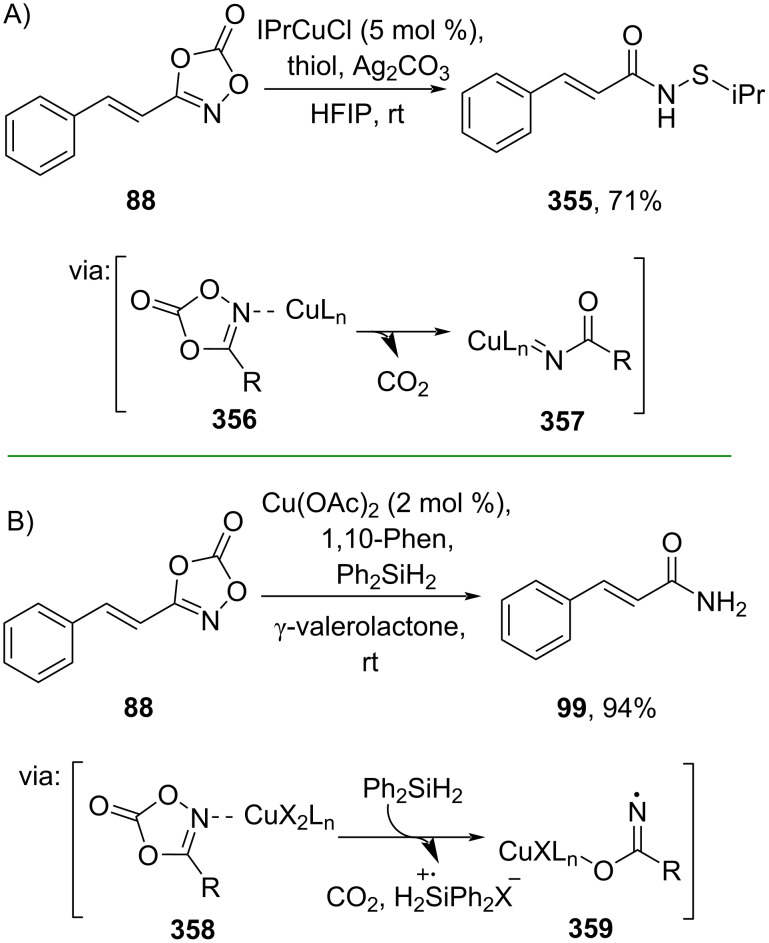
Cu-catalyzed dioxolane cleavage.

Furthermore, dioxolane compounds have also been subjected to synthesize cinnamic acid derivatives through CO_2_ release. For example, Chen and co-workers (2022) prepared *N*-acyl sulfenamide **355** in good yield from dioxolane **88** and thiols through Cu catalysis via Cu–acyl-nitrenoid **357** (Scheme 79A) [133]. Recently, Son and co-workers (2024) also employed a Cu catalyst to cleave dioxolane **88** in the presence of silanes as the reductant affording cinnamamide (**99**) in excellent yield. The reaction proceeds via the decarboxylated amide–copper complex **359** (Scheme 79B) [134] and could be scaled-up to a gram scale reaction.

**Scheme 80:**
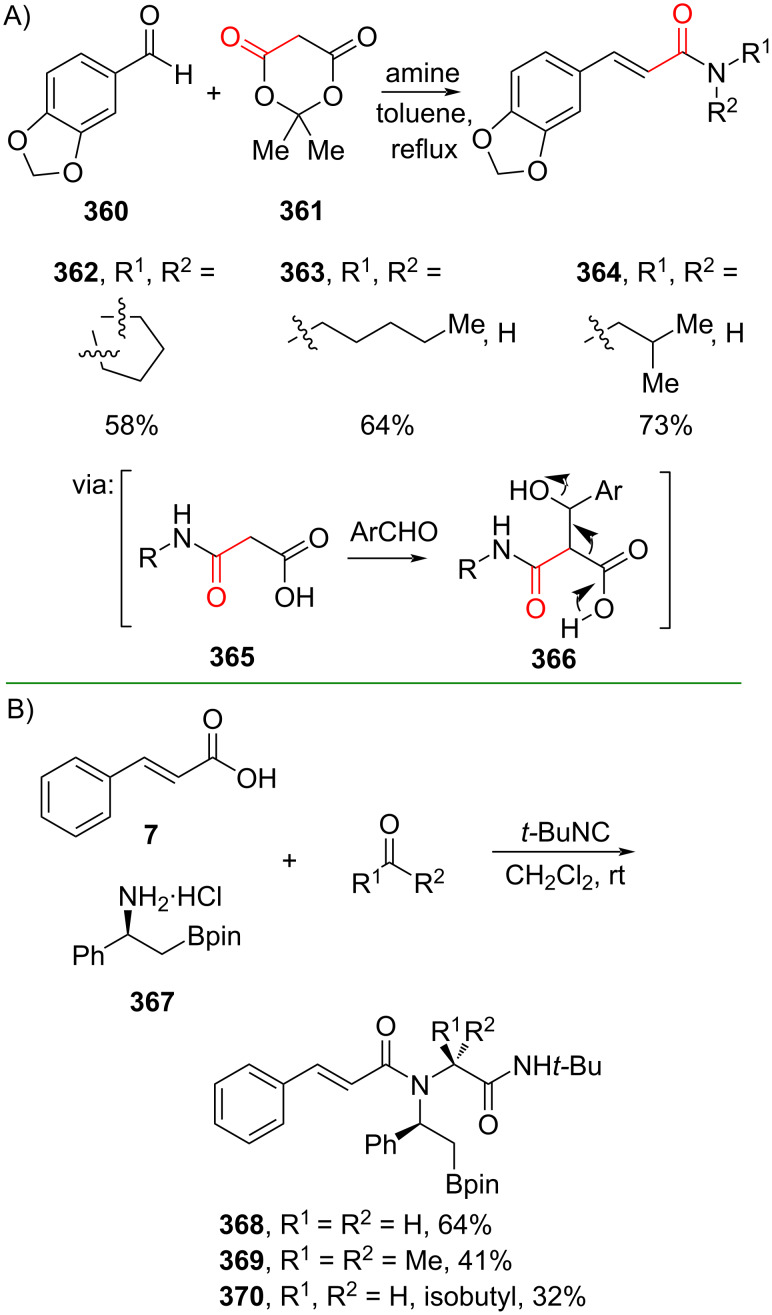
Multicomponent coupling reactions.

Cinnamic acid derivatives could also be accessed via multicomponent reactions. For example, Jana and Ghosh (2019) utilized Meldrum’s acid (**361**), aldehyde **360**, and an amine to prepare the corresponding cinnamamides **362–364**, natural products of the piperamide family. The multicomponent reaction involves consecutive acetone and CO_2_ release via **365** and **366** (Scheme 80A) [135]. A multigram scale operation has been achieved smoothly. Similarly, Silvani and co-workers (2022) also reported a multicomponent Ugi-type reaction of cinnamic acid (**7**), aldehyde/ketone, (*S*)-β-phenyl β-aminoboronate **367**, and *tert*-butyl isocyanide to give the corresponding pharmacophoric β-substituted β-amido boronates **368–370** in moderate yields (Scheme 80B) [136].

### 3 Double-bond functionalization

#### 3.1 Double-bond constructions

**Scheme 81:**
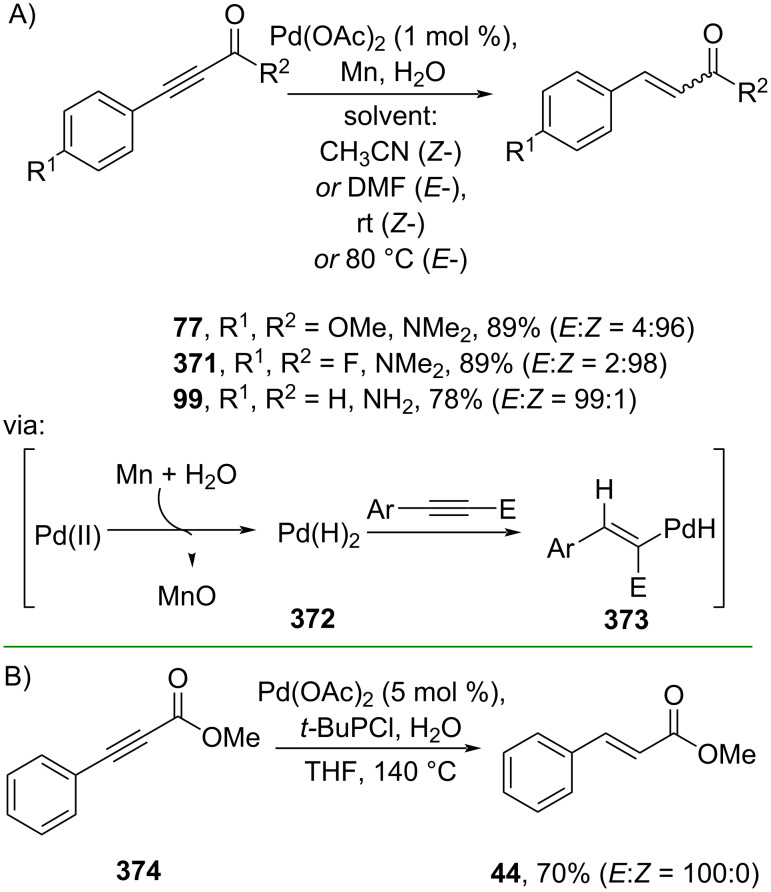
Pd-catalyzed partial hydrogenation of electrophilic alkynes.

**3.1.1 Conjugated alkyne hydrogenation:** Mei and co-workers (2019) developed a Pd-catalyzed partial hydrogenation of conjugated alkynes in the presence of water as the hydrogen source and Mn as the reductant to give the corresponding cinnamamides **13**, **77**, **99**, and **371** in good yields via the active palladium–hydride species **372** (Scheme 81A) [137]. The *Z-*to-*E*-selectivity could be effectively tuned by changing the solvent and temperature from CH_3_CN to DMF and rt to 80 °C, respectively. Similarly, Wang and co-workers (2023) employed Pd to catalyze the partial hydrogenation of the conjugated alkyne **374**, also using water as the hydrogen source to give *trans*-methyl cinnamate (**44**). The reaction also proceeds via a palladium–hydride species (Scheme 81B) [138]. The method has been smoothly conducted in a gram scale operation.

**Scheme 82:**
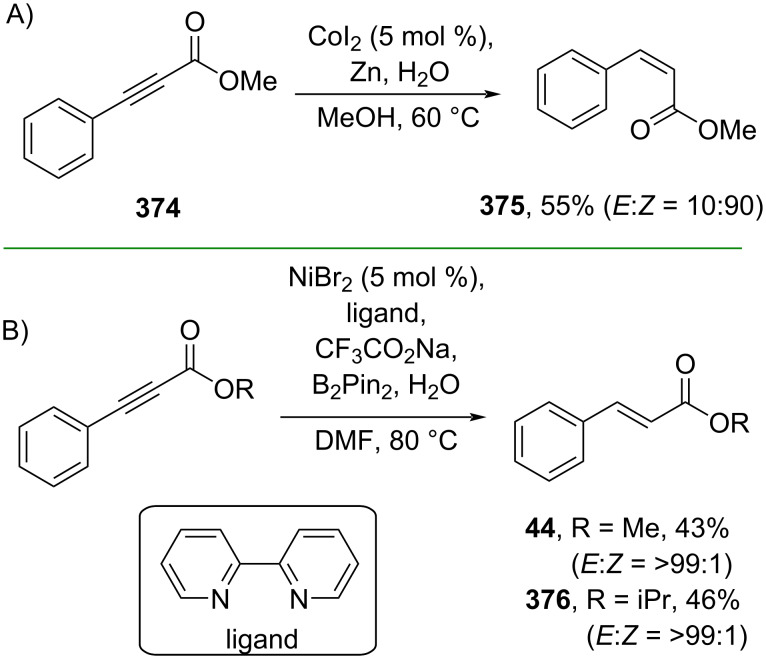
Nickel and cobalt as earth-abundant transition metals used as catalysts for the partial hydrogenation of conjugated alkynes.

More sustainable metal catalysts, such as earth-abundant transition metals (Ni and Co), have also been used to catalyze partial hydrogenation reactions of conjugated alkynes using environmentally benign water as the hydrogen source. For instance, Fan and co-workers (2019) reported the Co-catalyzed partial hydrogenation of the conjugated alkyne **374** to give *cis*-methyl cinnamate (**375**) in moderate yield via cobalt–hydride species (Scheme 82A) [139]. Similarly, Liu and co-workers (2023) utilized Ni to catalyze partial hydrogenation of conjugated alkynes to generate the corresponding *trans*-cinnamate esters **44** and **376** via nickel–hydride species (Scheme 82B) [140].

**Scheme 83:**
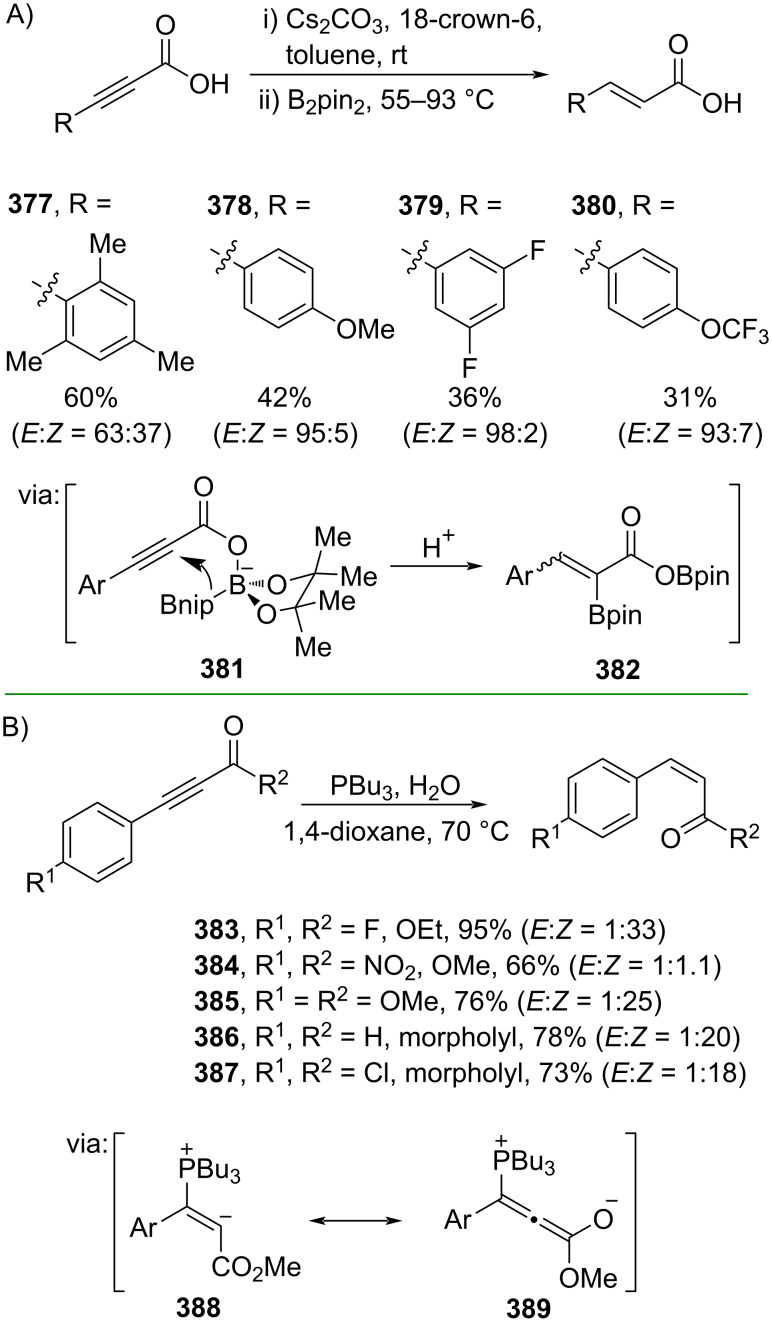
Metal-free-catalyzed partial hydrogenation of conjugated alkynes.

Furthermore, metal-free catalyzed partial hydrogenations of alkynes have also been explored. For instance, Santos and co-workers (2019) employed B_2_Pin_2_ to mediate the partial hydrogenation of alkynoic acids to generate the corresponding *trans*-cinnamic acids **377–380** via an α-borylation–protodeborylaton mechanism involving intermediates **381** and **382** (Scheme 83A) [141]. On the one hand, Vilotijevic and co-workers (2021) reported a phosphine-mediated partial hydrogenation of conjugated alkynes using water as the hydrogen source to obtain the corresponding *cis*-cinnamic acid esters **383–385** and amides **386** and **387** via zwitterionic allene species **389** (Scheme 83B) [142].

**Scheme 84:**
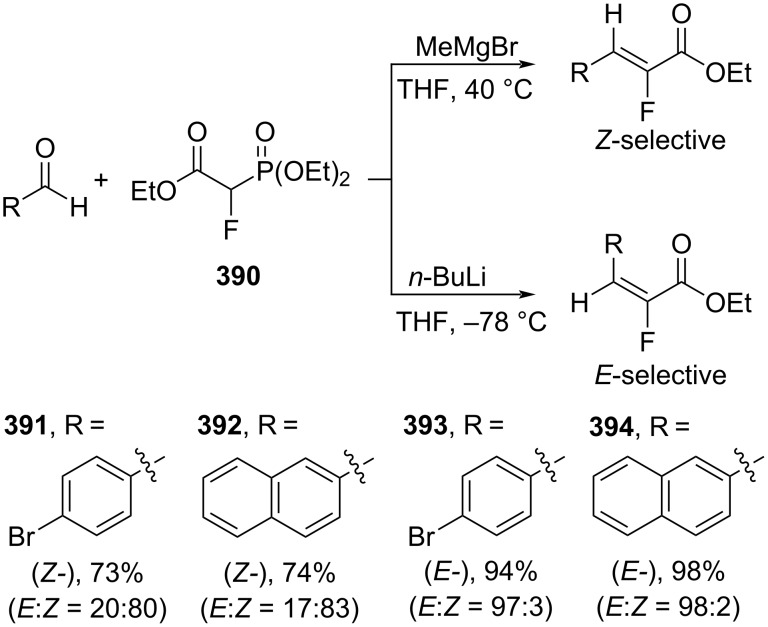
Horner–Wadsworth–Emmons reaction between triethyl 2-fluoro-2-phosphonoacetate and aldehydes with either MeMgBr or *n*-BuLi as the base.

**3.1.2 Ylide reactions:** The construction of double bond ylide reactions (e.g. Wittig and Horner–Wadsworth–Emmons reactions) is one of the most reliable and stereoselective methods. Several advancements in this area have recently been demonstrated to access conjugated alkenes, particularly cinnamic acid derivatives, with high stereoselectivity. For instance, Reeves and co-workers (2023) performed a Horner–Wadsworth–Emmons reaction of triethyl 2-fluoro-2-phosphonoacetate (**390**) and aldehydes to give the corresponding ethyl α-fluorocinnamates **391**–**394**. When using MeMgBr the products **391**, **392** were obtained with high (*Z*)-selectivity (Scheme 84). However, when applying *n*-BuLi as the base instead of MeMgBr the reaction afforded the corresponding (*E*)-configured products **393**, **394** [143].

**Scheme 85:**
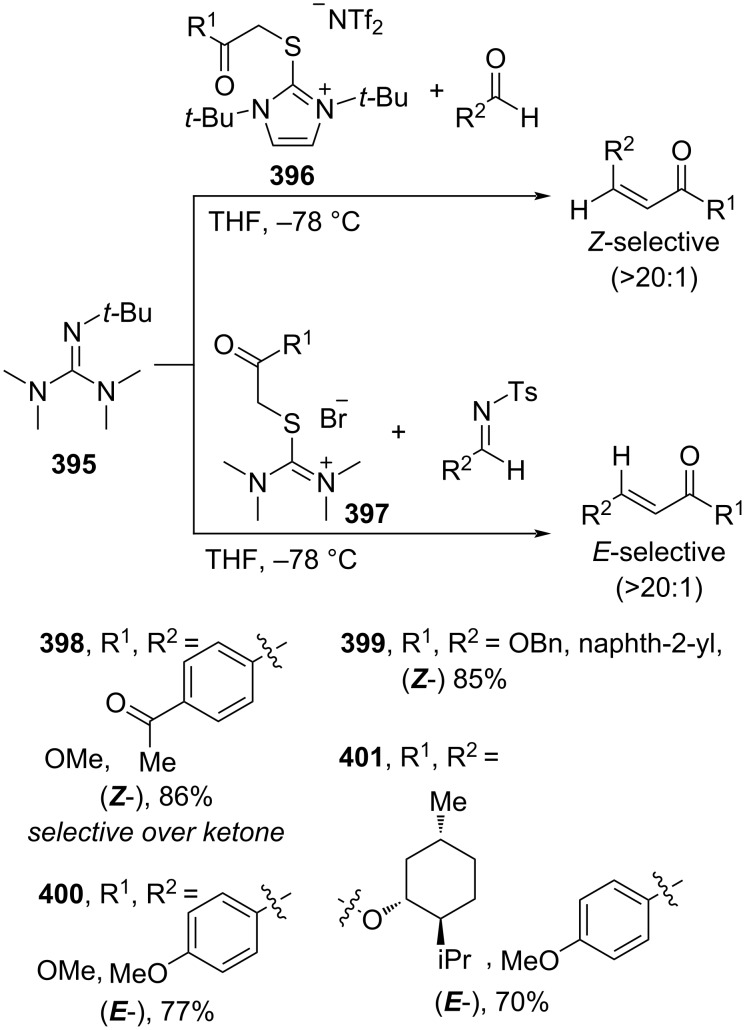
Preparation of *E*/*Z*-cinnamates using thiouronium ylides.

Moreover, Maulide and co-workers (2022) utilized a novel thiouronium ylide **396** and 2-(*tert*-butyl)-1,1,3,3-tetramethylguanidine (**395**) for the olefination of aldehydes to generate the corresponding cinnamates **398** and **399** with excellent (*Z*)-selectivity (Scheme 85). Interestingly, exchanging aldehydes for tosylimines and using ylide **397** altered the stereoselectivity to *E*-isomeric products **400** and **401** [144].

**Scheme 86:**
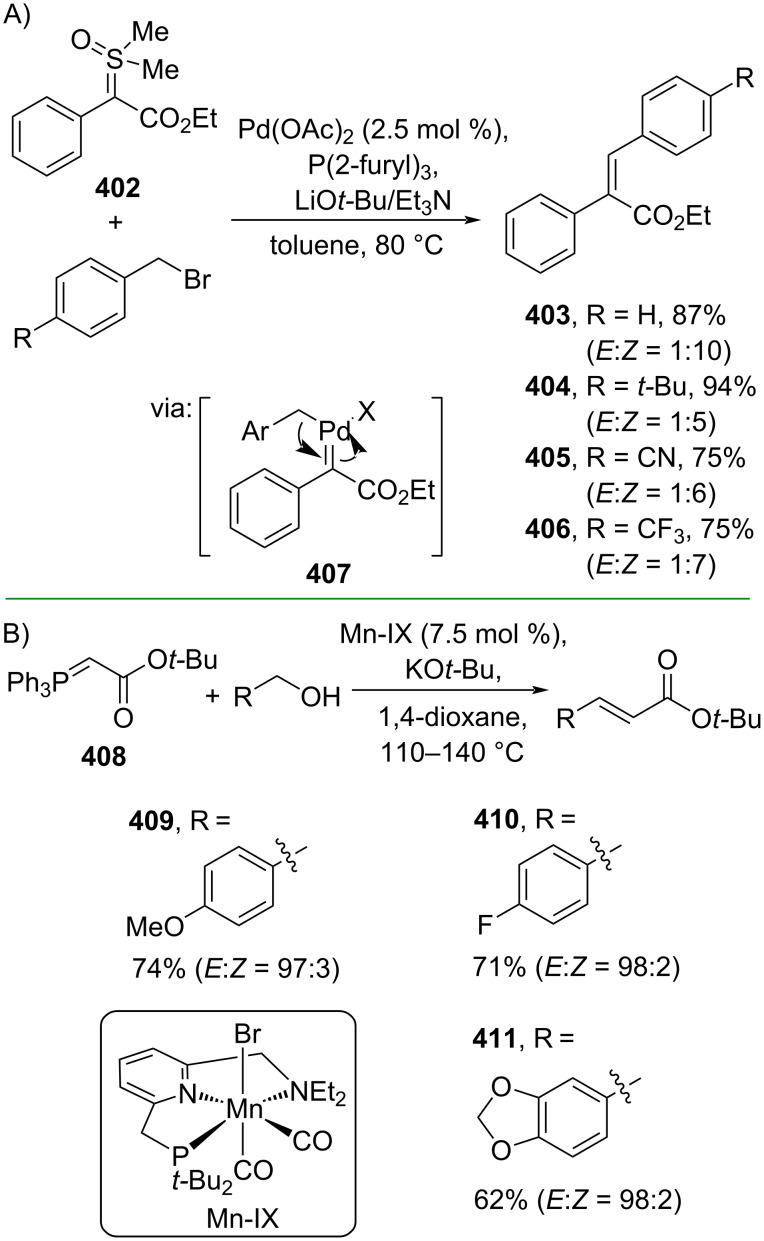
Transition-metal-catalyzed ylide reactions.

Wu and co-workers (2020) employed Pd to mediate the cross-coupling reaction of sulfoxonium ylide **402** and benzyl bromides to give the corresponding (*Z*)-ethyl cinnamates **403–406** in good yields via carbene migratory insertion (**407**) (Scheme 86A) [145]. On the other hand, Werner and Liu (2020) reported a Mn-catalyzed coupling of alcohols and phosphorus ylide **408** to afford the corresponding *E*-isomers of cinnamate esters **409–411** via an acceptorless dehydrogenative coupling mechanism (Scheme 86B) [146]. In addition, the method could be scaled up to a gram scale.

**Scheme 87:**
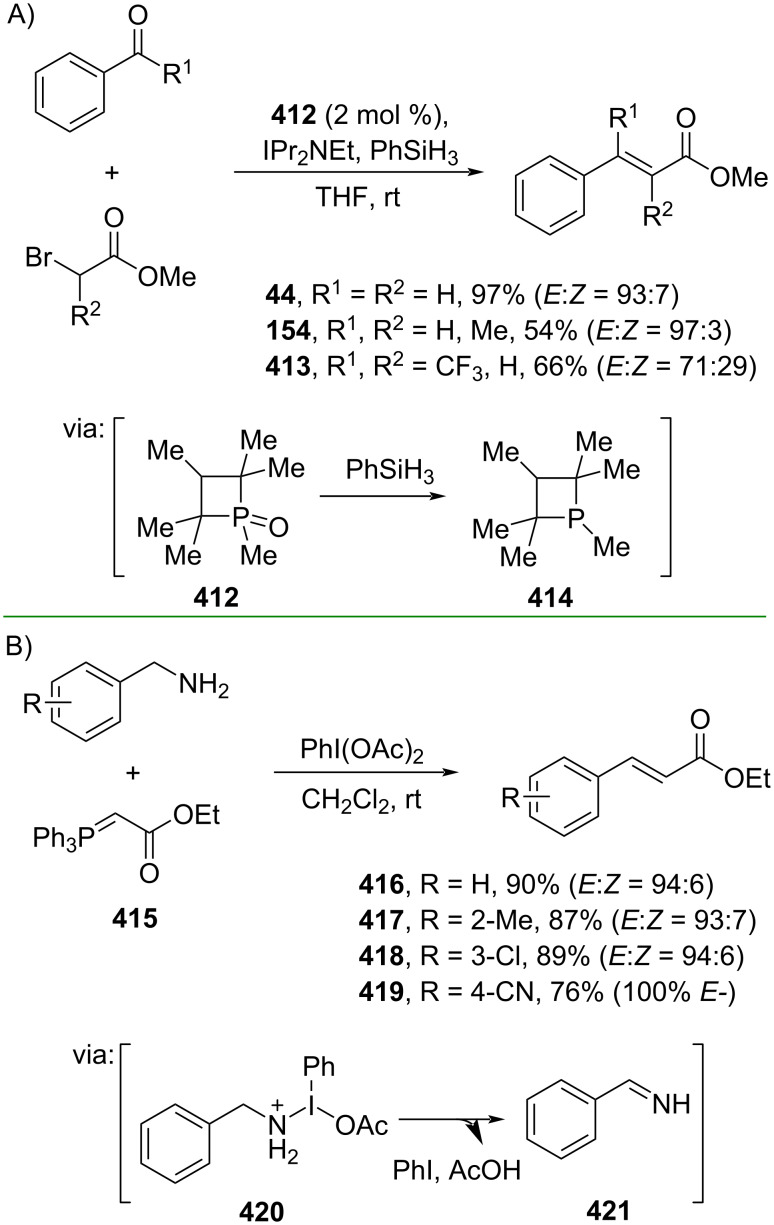
Redox-driven ylide reactions.

Redox-driven ylide reactions have gained increasing attention to tackle several disadvantages of traditional ylide-type reactions, such as use of reagents in excess and harsh reaction conditions. For example, Werner and co-workers (2019) reported a catalytic Wittig reaction using phosphetane oxide **412** as redox cycling catalyst using silanes as the reductant to convert aldehydes and α-bromoesters into the corresponding cinnamate esters **44**, **154**, and **413**. The reaction proceeds via formation of nucleophilic phosphane **414** (Scheme 87A) [147]. Moreover, Suryavanshi and co-workers (2020) utilized PhI(OAc)_2_ to mediate the oxidative olefination of amines and Wittig reagents **415** to give the corresponding cinnamate esters **416–419** via formation of imines **421** (Scheme 87B) [148]. In addition, a gram scale operation has been conducted smoothly.

**Scheme 88:**
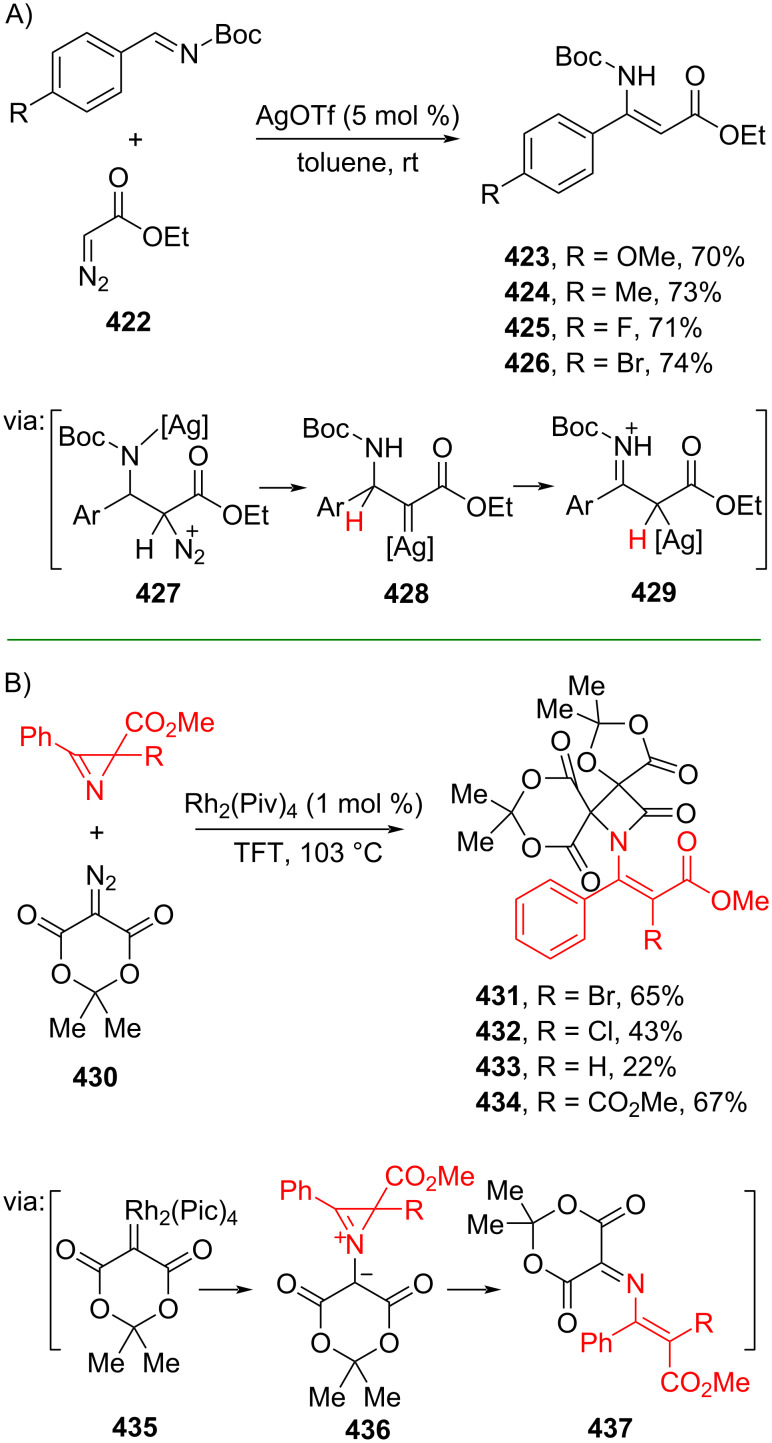
Noble transition-metal-catalyzed olefination via carbenoid species.

**3.1.3 Carbene/carbenoid reactions:** α-Diazocarbonyl compounds are useful reagents especially to synthesize aziridine compounds through reactive carbene species. A few examples of utilizing α-diazocarbonyl compounds to prepare olefins have also been reported, either mediated by metal catalysts or through a more sustainable way via a non-metal strategy. For instance, Liu and Kardile (2019) reported Ag(I)-catalyzed olefinations of α-diazoester **422** and *N*-Boc-protected imines to afford the corresponding β-aryl-β-aminoacrylates **423–426** via formation of a Mannich–addition intermediate **428** which undergoes 1,2-hydride migration to **429** (Scheme 88A) [149]. On the other hand, Novikov and co-workers (2019) utilized Rh(II) to catalyze the [2 + 1 + 1] assembly of spiro β-lactams **431–434** from diazocarbonyl compound **430** and azirines via Rh carbenoid **435** followed by aziridine ring-opening (**436** and **437**) (Scheme 88B) [150].

**Scheme 89:**
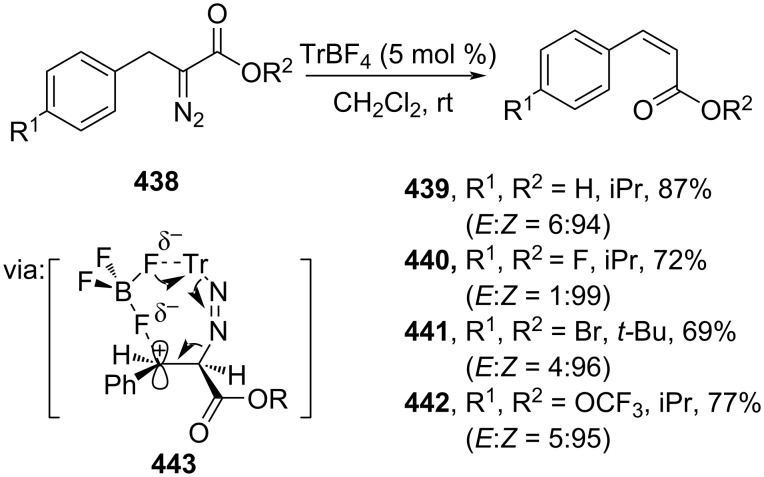
TrBF_4_-catalyzed olefination via carbene species.

Lv and co-workers (2019) employed a non-metal Lewis acid tritylium salt (TrBF_4_) to catalyze the stereoselective olefination of α-diazocarbonyl compounds **438** to access *Z*-cinnamate esters **439**–**442** via 1,2-hydride migration (**443**) (Scheme 89) [151]. The ion pair of carbocation, BF_4_^−^ anions, and the trityldiazene group are *cis*-coplanar resulting in the *Z*-isomer product.

**Scheme 90:**
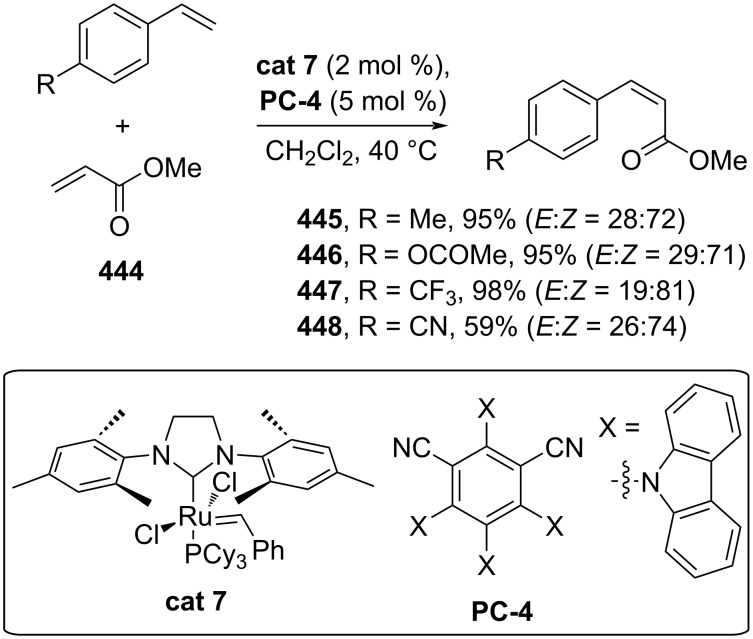
Grubbs catalyst (**cat 7)**/photocatalyst-mediated metathesis reactions.

**3.1.4 Metathesis reactions:** Metathesis reactions are one of the most crucial approaches to preparing olefins. Grubbs and Grubbs–Hoveyda catalysts are among the most frequently used catalysts for the stereoselective construction of C=C bonds. Moreover, metathesis reactions have also been applied to stereoselectively synthesize cinnamic acid derivatives. For example, Lakhdar and co-workers (2022) combined *E*-selective Grubbs second-generation catalysts (**cat 7**) with photocatalyst (**PC-4**) to convert styrenes and methyl acrylate (**444**) into the corresponding (*Z*)-cinnamic acid esters **445–448** in excellent yields via *E*-to-*Z* photoisomerization mediated by the photocatalyst (Scheme 90) [152].

**Scheme 91:**
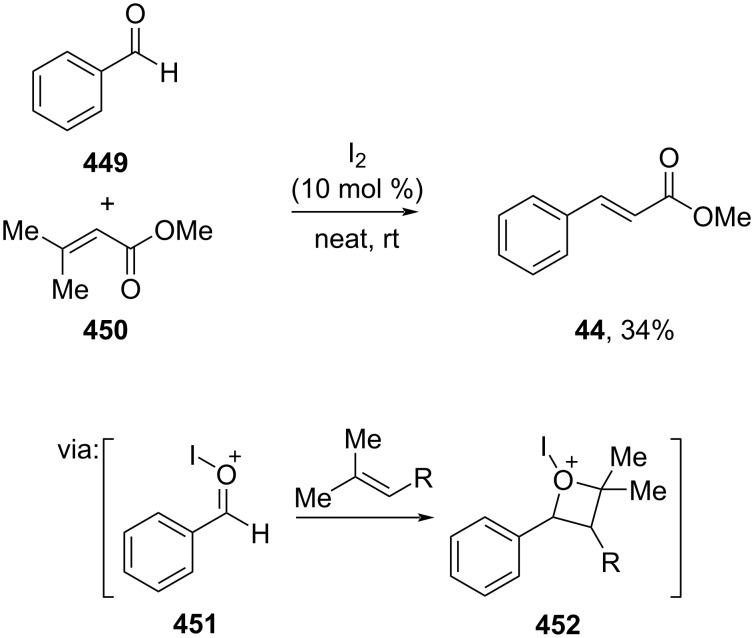
Elemental I_2_-catalyzed carbonyl-olefin metathesis.

Nguyen and co-workers (2019) employed iodine to catalyze the intermolecular olefin-carbonyl metathesis reaction of benzaldehyde (**449**) and acrylate **450** to give the corresponding methyl cinnamate (**44**) via Lewis acidic I^+^-activated carbonyl intermediate **451** followed by formation of oxetane intermediate **452** (Scheme 91) [153]. Despite the low yield, this approach offers an alternative for catalyst simplicity, potentially leading to more advancements in applications using iodine as catalyst.

**Scheme 92:**
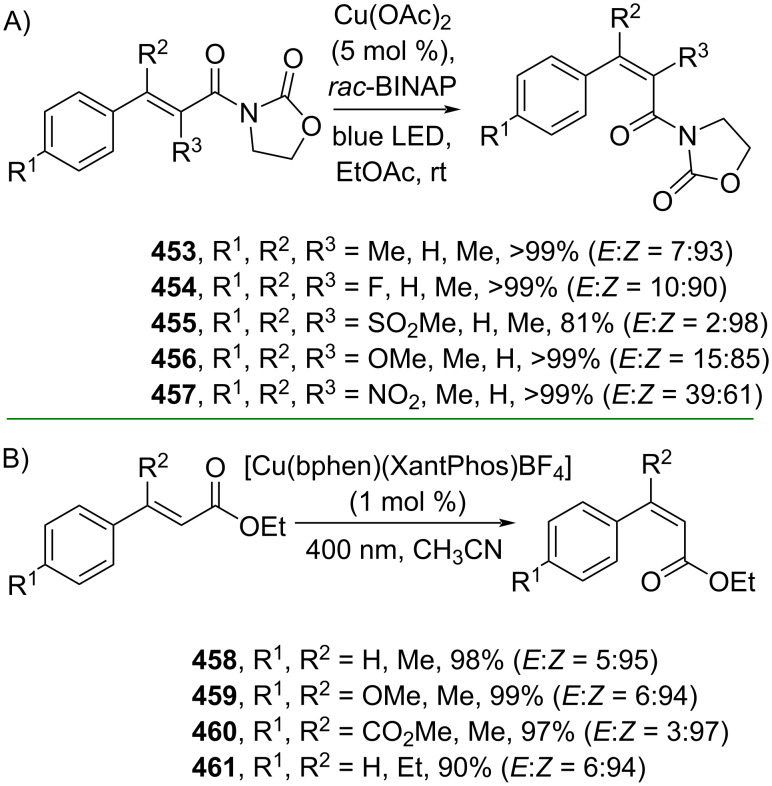
Cu-photocatalyzed *E*-to-*Z* isomerization of cinnamic acid derivatives.

**3.1.5 *E*/*Z* isomerization:** Numerous bioactive cinnamic acid derivatives are not limited to *E*-isomers. Instead, multiple studies have reported high activities of (*Z*)-cinnamic acid derivatives, such as anti-tyrosinase activity and plant growth stimulation [14,154]. Several methods to access (*Z*)-cinnamic acid derivatives have been occasionally demonstrated in the previous chapters, however, direct methods to convert *E*- to *Z*-isomers have gained more interest due to their sustainable values, such as catalytic transformation and atom economy. Although the *E*-to-*Z* conversion is thermodynamically counterproductive, some strategies have been reported to achieve the desired (*Z*)-cinnamic acid derivatives. For instance, Poisson and co-workers (2020) reported a Cu-catalyzed *E*-to-*Z* isomerization of α/β-substituted cinnamamides under blue LED irradiation via a singlet-state mechanism (Scheme 92A) [155]. On the other hand, Collins and co-workers (2021) reported a heteroleptic Cu-based photosensitizer for the *E*-to-*Z* isomerization of cinnamate esters **458–461** with excellent yields via energy transfer (Scheme 92B) [156].

**Scheme 93:**
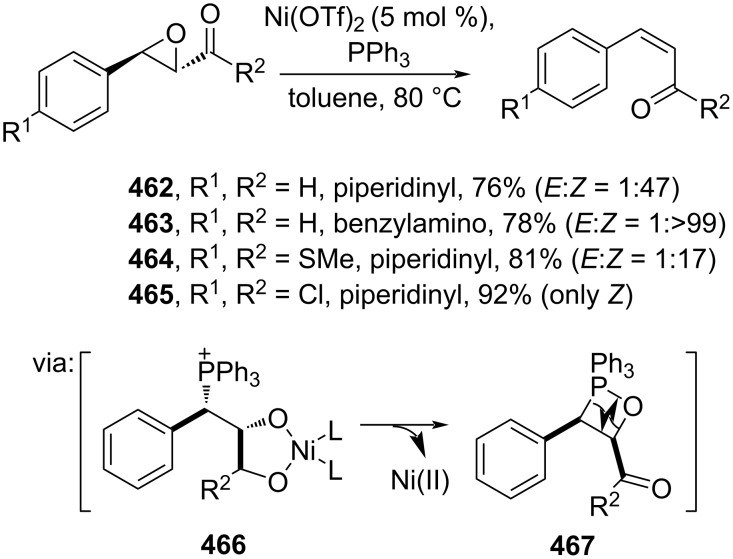
Ni-catalyzed *E*-to-*Z* isomerization.

Recently, Akkarasamiyo and co-workers (2024) reported a Ni-catalyzed *E*-to-*Z* isomerization of (*E*)-epoxycinnamamides to (*Z*)-cinnamamides **462–465**. The reaction proceeds via a Ni-induced epoxide-ring opening (**466**, **467**) following phosphine attack (Scheme 93) [157]. In addition, a gram-scale reaction has been successfully demonstrated.

**Scheme 94:**
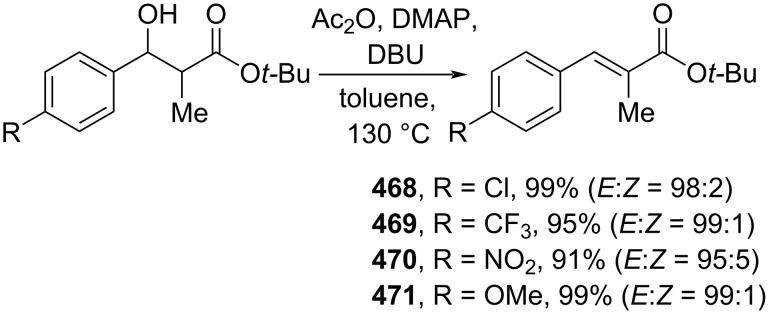
Dehydration of β-hydroxy esters via an E1cB mechanism to access (*E*)-cinnamic acid esters.

**3.1.6 Elimination reactions:** Elimination reactions are usually straightforward due to the considerably low substrate or reagent pretreatment and frequently, this simple procedure leads to highly stereoselective transformations. For example, Kawasaki and co-workers (2019) reported the highly *E*-selective dehydration of α-substituted-β-hydroxyesters in the presence of a base to give the corresponding (*E*)-cinnamate esters **468–471** in excellent yields. The reaction follows an E1cB mechanism (Scheme 94) [158].

**Scheme 95:**
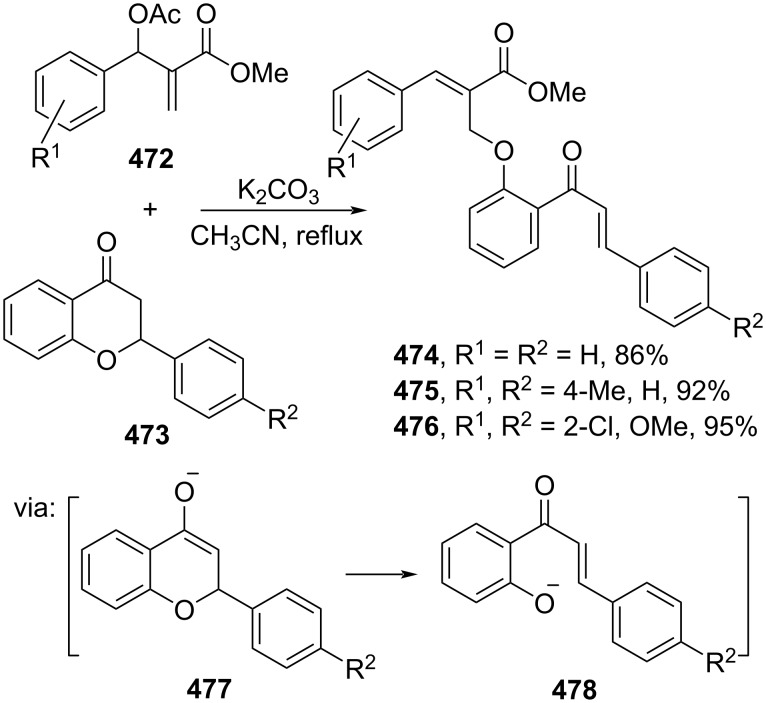
Domino ring-opening reaction induced by a base.

Bakthadoss and co-workers (2021) employed Baylis–Hillman acetates **472** and 2-arylchromanones **473** to afford the corresponding β-substituted cinnamate esters **474–476** in the presence of a base via base-induced C–O bond cleavage (**477**, **478**) followed by addition reaction towards the acetate (Scheme 95) [159].

**Scheme 96:**
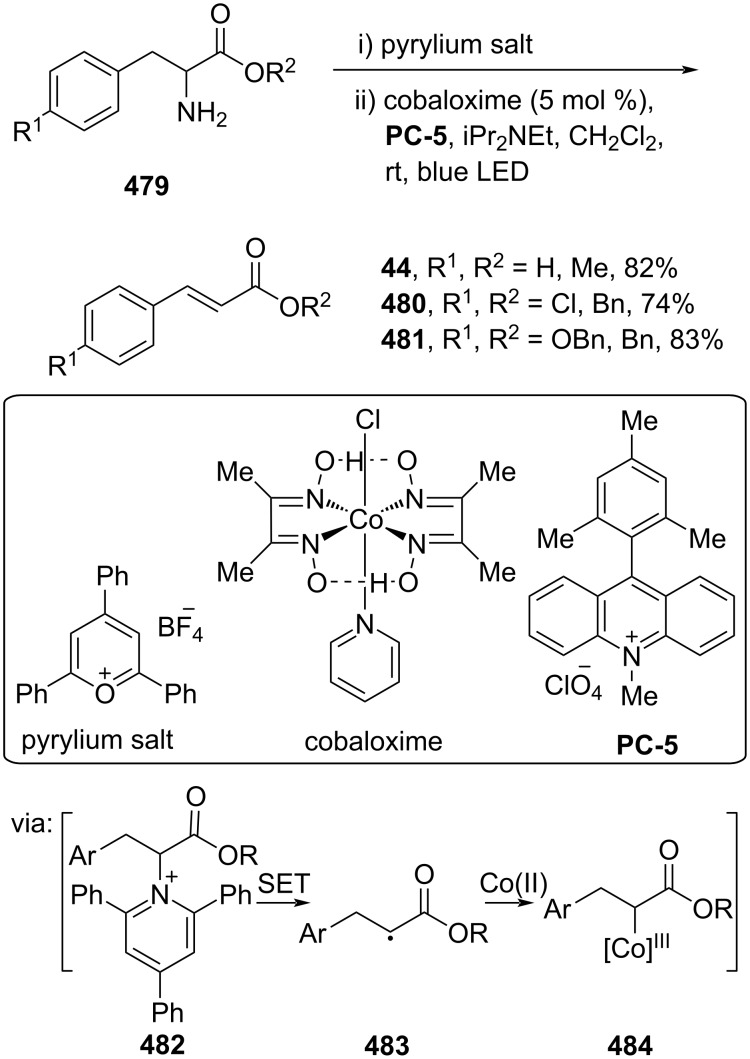
Dehydroamination of α-aminoester derivatives.

Furthermore, elimination reactions could also be achieved through photocatalysis. For example, El-Sepelgy and co-workers (2023) reported a Co-catalyzed dehydroamination of α-aminoester derivatives **479** to give the corresponding cinnamate esters **44**, **480**, and **481** with excellent *E*-selectivity (>20:1) under blue LED irradiation via SET (**482** and **483**) followed by Co(II) insertion (**484**) (Scheme 96) [160].

**Scheme 97:**
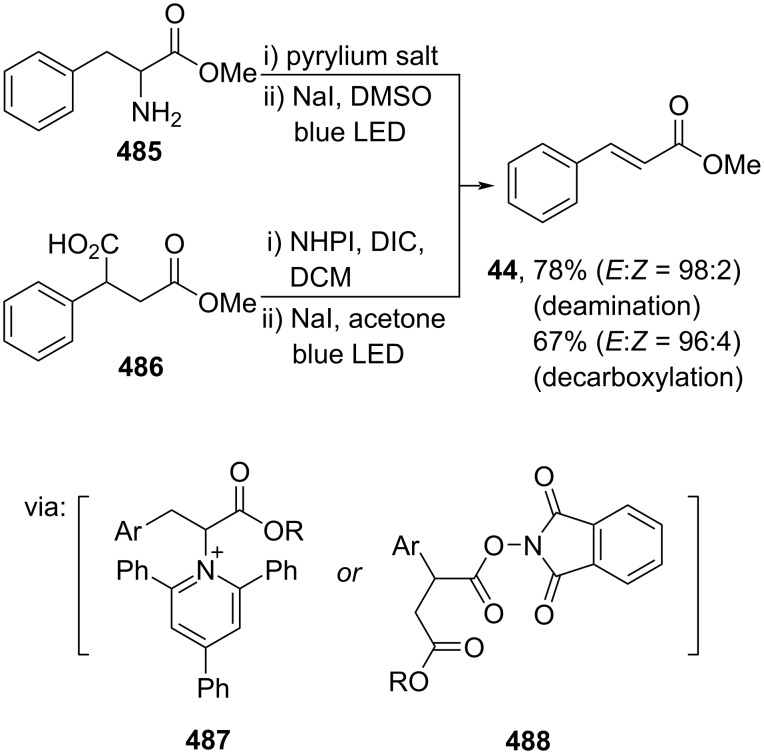
Accessing methyl cinnamate (**44**) via metal-free deamination or decarboxylation.

Similarly, Chen and co-workers (2021) reported a transition-metal-free deamination of α-aminoester **485** mediated by NaI in the presence of a pyrylium salt under blue LED irradiation to give methyl cinnamate (**44**). The reaction demonstrates excellent *E*-selectivity and proceeds via Katritzky salt intermediate **487**, followed by radical cleavage (Scheme 97). The same method could also be applied to the decarboxylation of carboxylic acid **486** by using *N*-hydroxyphthalimide (NHPI) via NHPI ester intermediate **488** [161].

**Scheme 98:**
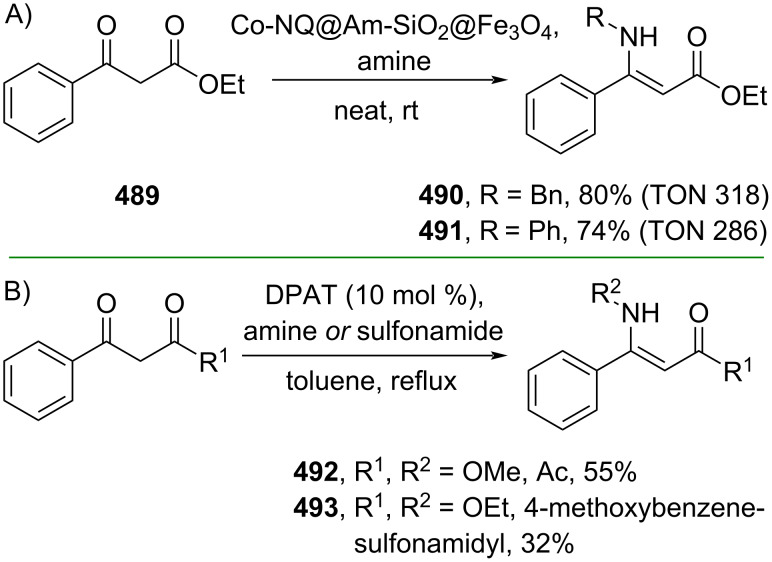
The core–shell magnetic nanosupport-catalyzed condensation reaction.

**3.1.7 Miscellaneous reactions:** Direct condensation of amines with 1,3-dicarbonyl compounds has been widely implemented to access β-enaminones, particularly β-amino-cinnamic acid derivatives. Some developments have been reported in this well-established area. For example, Sharma and co-workers (2023) developed an in situ-generated naphthoquinone–Co complex covalently immobilized on a silica-coated magnetite nanosupport to catalyze condensation reactions of amines and β-carbonylester **489** to give the corresponding β-enamino esters **490** and **491** in solvent-free conditions (Scheme 98A) [162]. The core-shell magnetic silica catalyst worked as a nanoreactor with high recyclability (TON up to 357) thus promoting sustainability goals. On the other hand, using a metal-free approach, Li and co-workers (2019) reported a diphenylammonium triflate (DPAT)-catalyzed condensation of β-carbonylesters with amines or 4-methoxybenzenesulfonamide to give the corresponding β-enaminones **492** and **493**, respectively, via a Brønsted acid–O=C activation mode (Scheme 98B) [163].

**Scheme 99:**
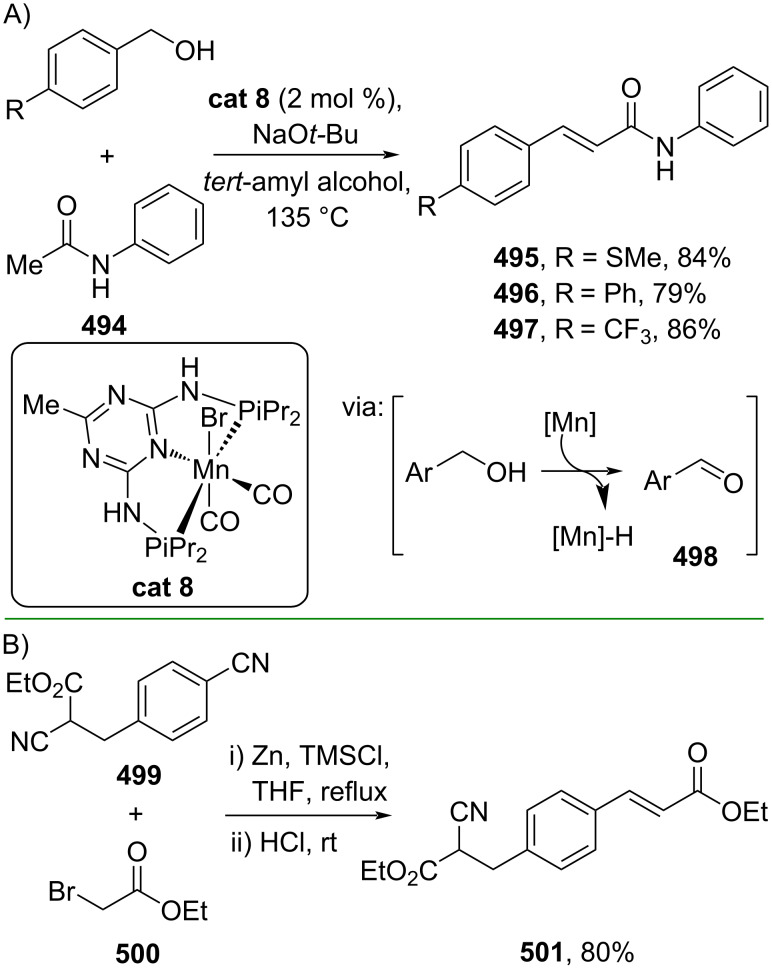
Accessing cinnamic acid derivatives from acetic acid esters/amides through α-olefination.

Moreover, the α-olefination of acetate esters or amides to synthesize the corresponding α,β-unsaturated esters and amides has been well documented. For instance, Gunanathan and Pandia (2021) reported a Mn-catalyzed α-olefination of acetamide **494** and alcohols to give the corresponding cinnamamides **495–497** through alcohol oxidation promoted by Mn resulting in the electrophilic aldehyde **498** and H_2_ (Scheme 99A) [164]. On the other hand, Rao and Padder (2020) performed a Blaise reaction of dinitrile ester **499** and ethyl α-bromoacetate (**500**) to afford the corresponding cinnamate ester **501** (Scheme 99B) [165].

**Scheme 100:**
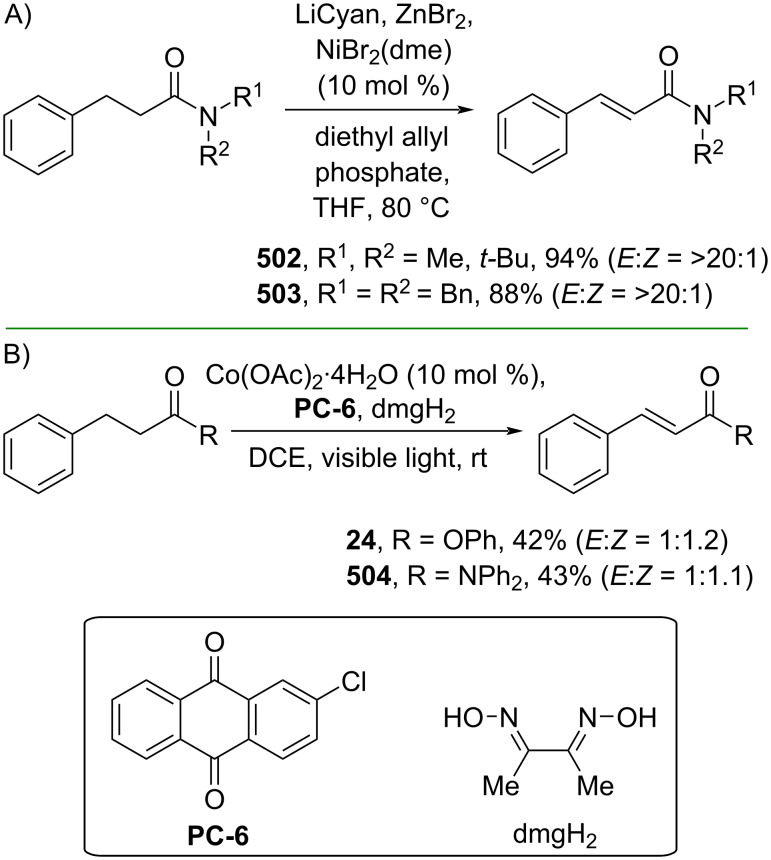
Accessing cinnamic acid derivatives via acceptorless α,β-dehydrogenation.

Cinnamic acid derivatives could also be obtained through a catalytic α,β-dehydrogenation strategy catalyzed predominantly by noble metals [166–167]. Recent developments in this area have utilized more sustainable catalyst sources, such as earth-abundant transition metals. For instance, Newhouse and co-workers (2019) reported a Ni-catalyzed acceptorless α,β-dehydrogenation of amides to give the corresponding cinnamamides **502** and **503** via a β-hydride elimination mechanism (Scheme 100A) [168]. Similarly, Huang and co-workers (2021) employed an organophotoredox/Co dual catalyst to mediate the acceptorless α,β-dehydrogenation of esters or amides to give the corresponding cinnamate ester **24** or cinnamamide **504**, respectively, under visible light irradiation via a β-hydride elimination process (Scheme 100B) [169].

**Scheme 101:**
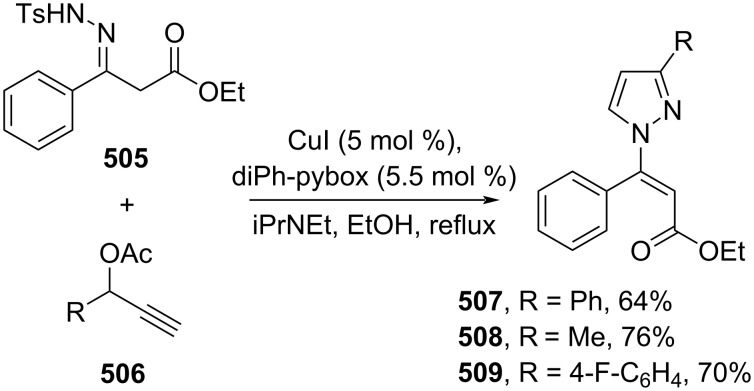
Cu-catalyzed formal [3 + 2] cycloaddition.

Cycloaddition reactions have also been applied to prepare cinnamic acid derivatives. For instance, Hu and co-workers (2020) reported a Cu-catalyzed formal propargylic [3 + 2] cycloaddition of 3-(2-tosylhydrazono)propanoate **505** with propargylic acetates **506** to afford the corresponding β-pyrazolyl acrylates **507–509** with excellent *E*-selectivity (Scheme 101) [170].

#### 3.2 Cα functionalization

**Scheme 102:**
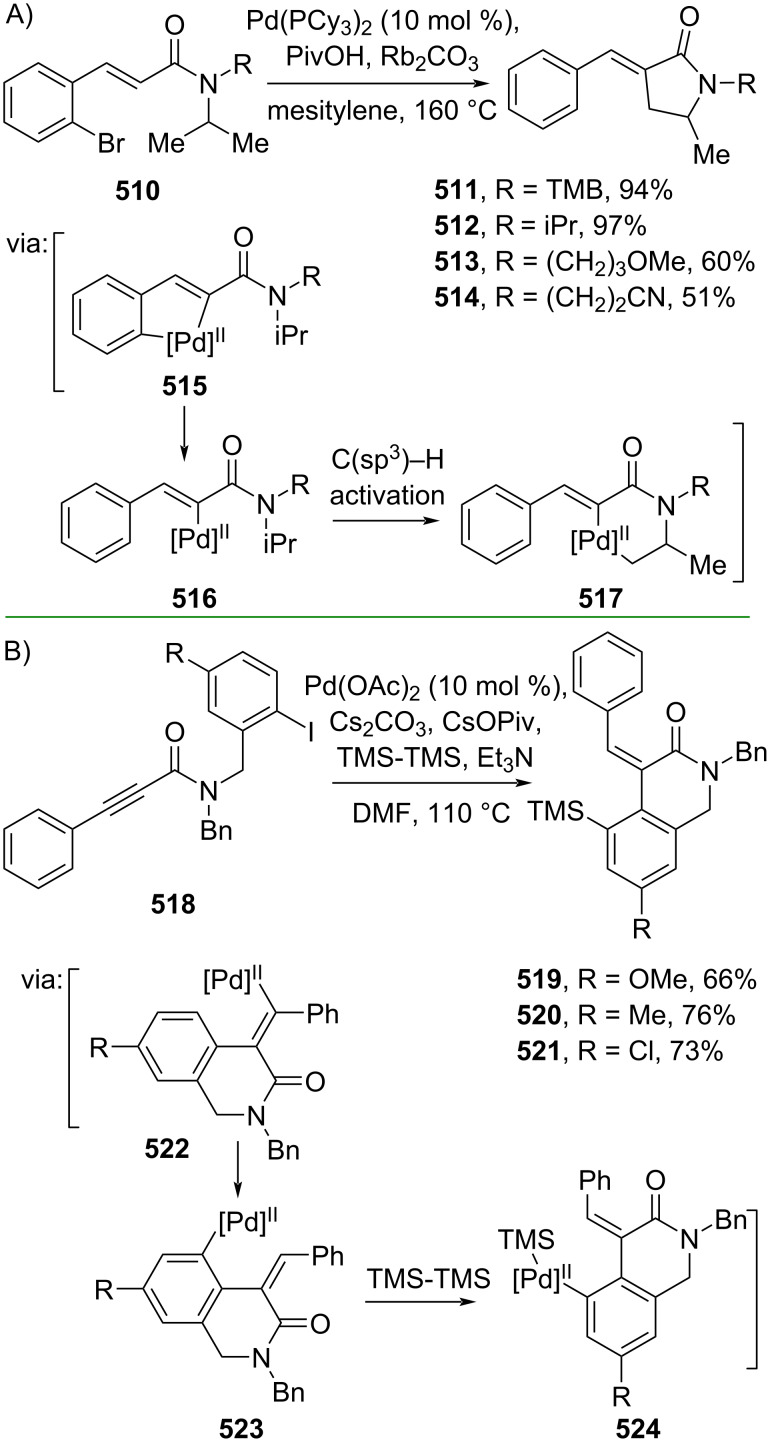
Pd-catalyzed C–C bond formation via 1,4-Pd-shift.

The direct Cα functionalization of cinnamic acid derivatives may offer a convenient strategy for preparing various α-substituted cinnamic acid derivatives, particularly during the hit-to-lead stage of drug discovery. Several methods employing metal and metal-free catalysts have been developed to fulfill this purpose. For example, Baudoin and Rocaboy (2019) reported the conversion of cinnamamides **510** to the corresponding α-arylidene γ-lactams **511**–**514** via a 1,4-Pd shift (**515**, **516**) enabling C(sp^3^)–H activation (**517**) (Scheme 102A) [171]. Similarly, Zhang and co-workers (2021) conducted a Pd-catalyzed synthesis of 6-membered lactams **519–521** from alkyne-tethered aryl iodides **518** through 1,4-Pd shift (**522**, **523**) followed by C(sp^2^)–H silylation (**524**) (Scheme 102B) [172].

**Scheme 103:**
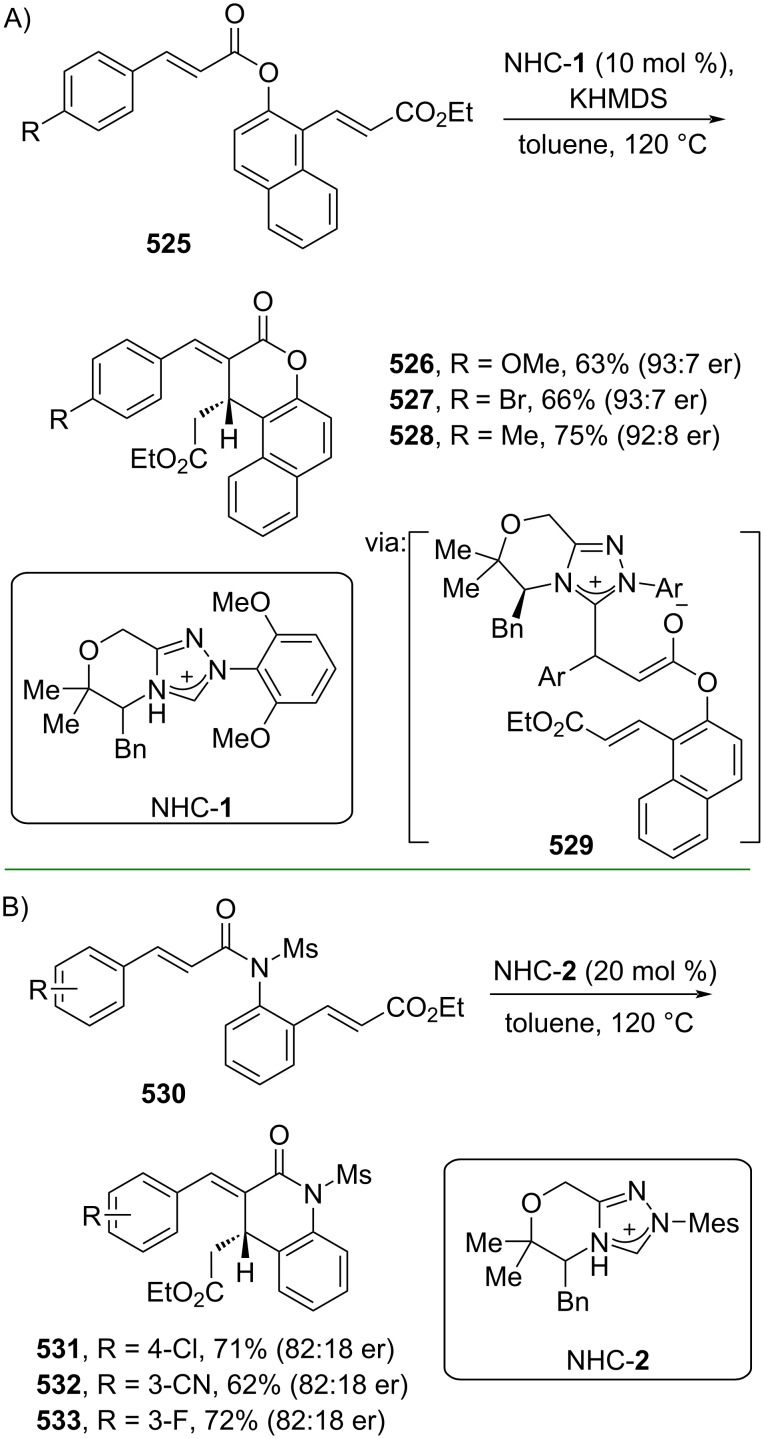
NHC-catalyzed Rauhut–Currier reactions.

In addition, the metal-free catalyzed synthesis of 6-membered lactam formation has been explored through Rauhut–Currier reactions. For instance, Lupton and co-workers (2019) utilized *N*-heterocyclic carbene (NHC-**1**) to catalyze the Rauhut–Currier reaction of bis(enoate) **525** to afford the corresponding hydrocoumarins **526–528** with excellent enantioselectivity (>20:1 dr) via intermediate **529** (Scheme 103A) [173]. The same group (2021) also reported an NHC (NHC-**2**)-catalyzed Rauhut–Currier reaction towards β-substituted acrylamides **530** to give the corresponding 2-quinolones **531–533** with good diastereoselectivity (Scheme 103B) [174].

**Scheme 104:**
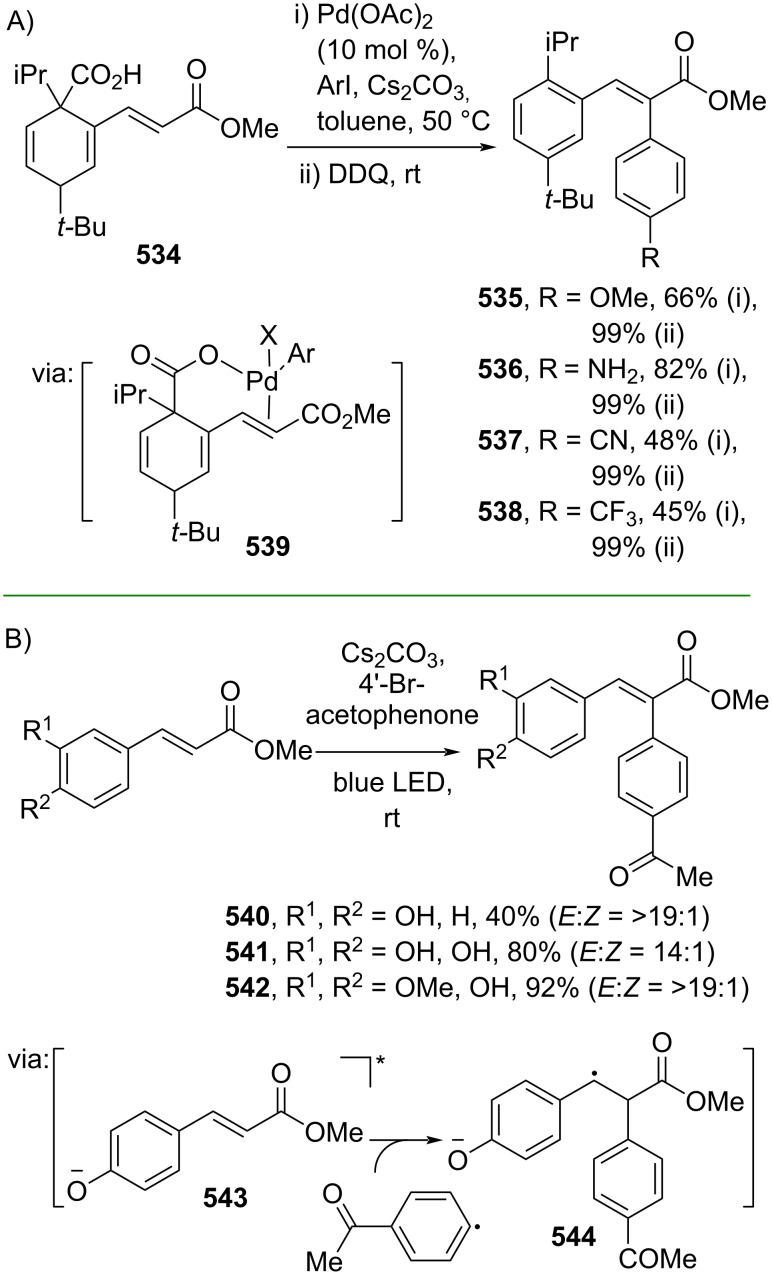
Heck-type reaction for Cα arylation.

The intermolecular Cα functionalization of cinnamic acid derivatives has been accomplished through several strategies, including Mizoroki–Heck coupling reactions and photocatalysis. For example, Chou and co-workers (2021) performed a regioselective Mizoroki–Heck coupling of β-cyclohexadienyl acrylate **534** with aryl iodides followed by decarboxylative aromatization to give the corresponding α-arylated cinnamate esters **535**–**538** (Scheme 104A) [175]. In this work, the carboxylate group directed the Pd catalyst insertion into the conjugated double bond via coordination **539**. Furthermore, Xia and co-workers (2020) performed a metal-free-photochemical Heck-type coupling of hydroxylated cinnamate esters and 4’-bromoacetophenone under blue LED irradiation to obtain the corresponding α-arylated cinnamate esters **540**–**542** in good yields. The reaction proceeds through visible light excitation followed by a single-electron transfer (SET) process (**543** and **544**) (Scheme 104B) [176].

**Scheme 105:**
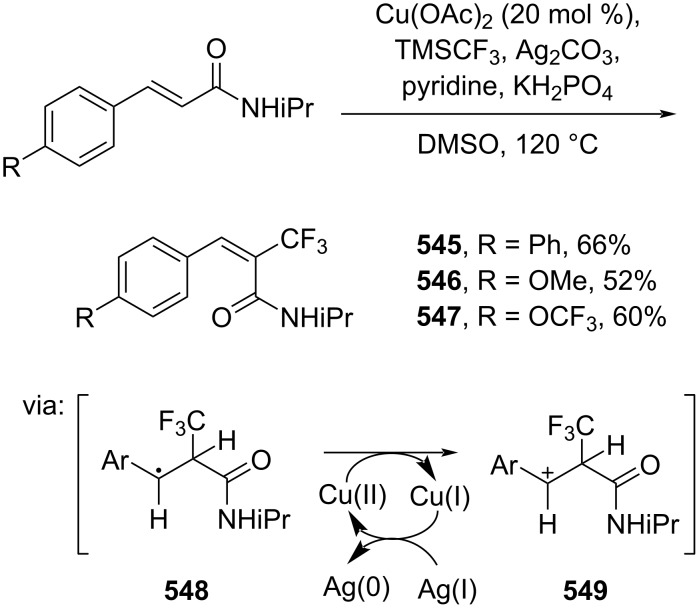
Cu-catalyzed trifluoromethylation of cinnamamide.

Dai and co-workers (2019) reported a Cu-catalyzed α-selective trifluoromethylation of cinnamamides using TMSCF_3_ (Scheme 105) [177]. In the presence of Ag(I), TMSCF_3_ was converted into CF_3_ radical species leading to the Cα attacked via single electron transfer (SET) (**548**) followed by benzylic carbocation **549** formation promoted by Cu(II).

**Scheme 106:**
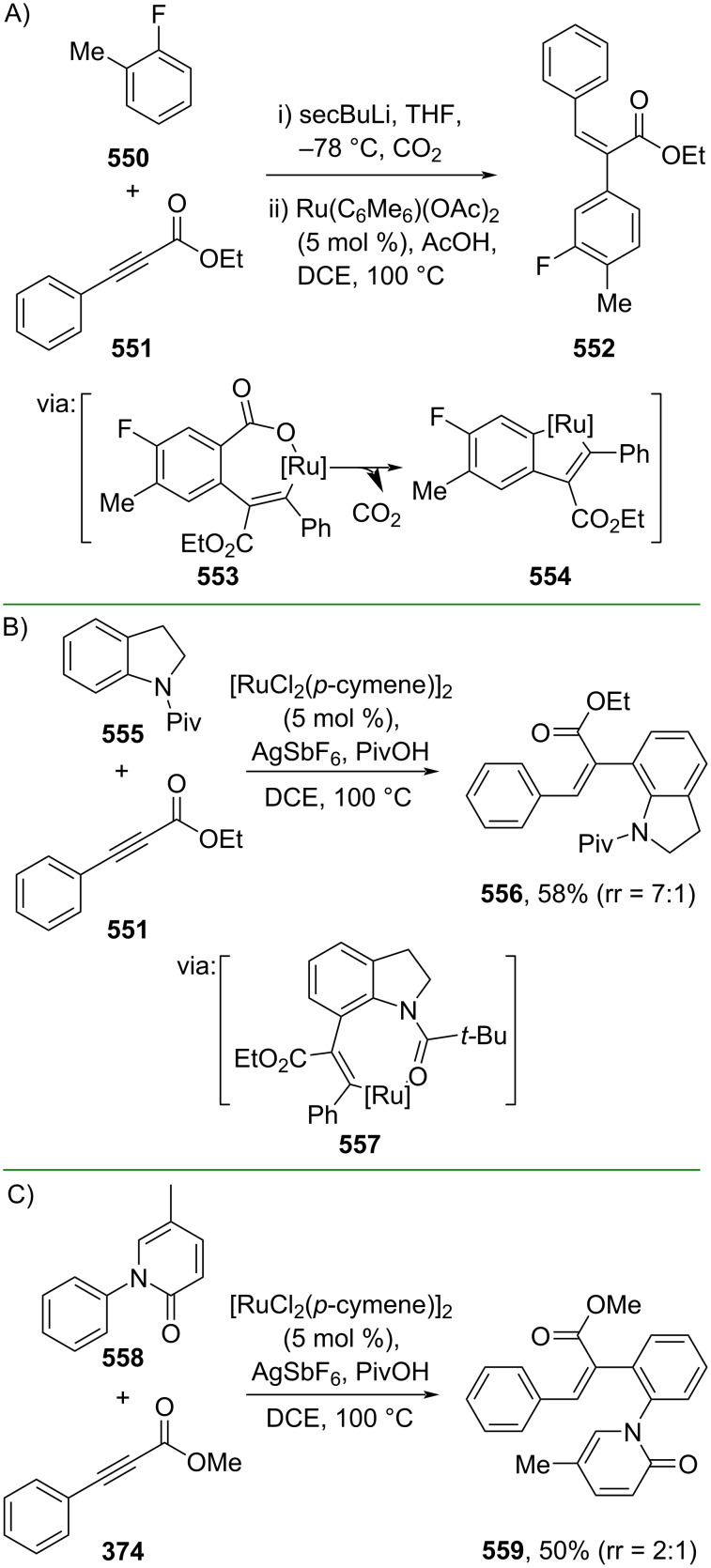
Ru-catalyzed alkenylation of arenes using directing groups.

In addition, alkynes have been subjected to hydroarylation reactions to access α-substituted cinnamic acid derivatives. For example, Larrosa and co-workers (2020) utilized CO_2_ as a traceless carboxylate directing group for *meta*-selective Ru-catalyzed olefination of fluoroarene **550** with α,β-alkynyl ester **551** to generate the corresponding (*Z*)-α-arylated cinnamate ester **552** (Scheme 106A) [178]. Herein, the Ru complex with the carboxylated fluoroarene led to alkyne insertion via **553** followed by decarboxylation to form metallacycle intermediate **554**. Similarly, Koley and co-workers (2021) reported a Ru-catalyzed alkenylation of indoline **555** with α,β-alkynyl ester **551** to give the corresponding (*Z*)-α-arylated cinnamate ester **556** using *N*-protected pivalic group as the internal directing group (**557**) (Scheme 106B) [179]. Later, the same group (2022) also reported the Ru-catalyzed alkenylation of pirfenidone (**558**) with α,β-alkynyl ester **374** to give the corresponding (*Z*)-α-arylated cinnamate ester **559** with pyridone acting as the directing group (Scheme 106C) [180].

**Scheme 107:**
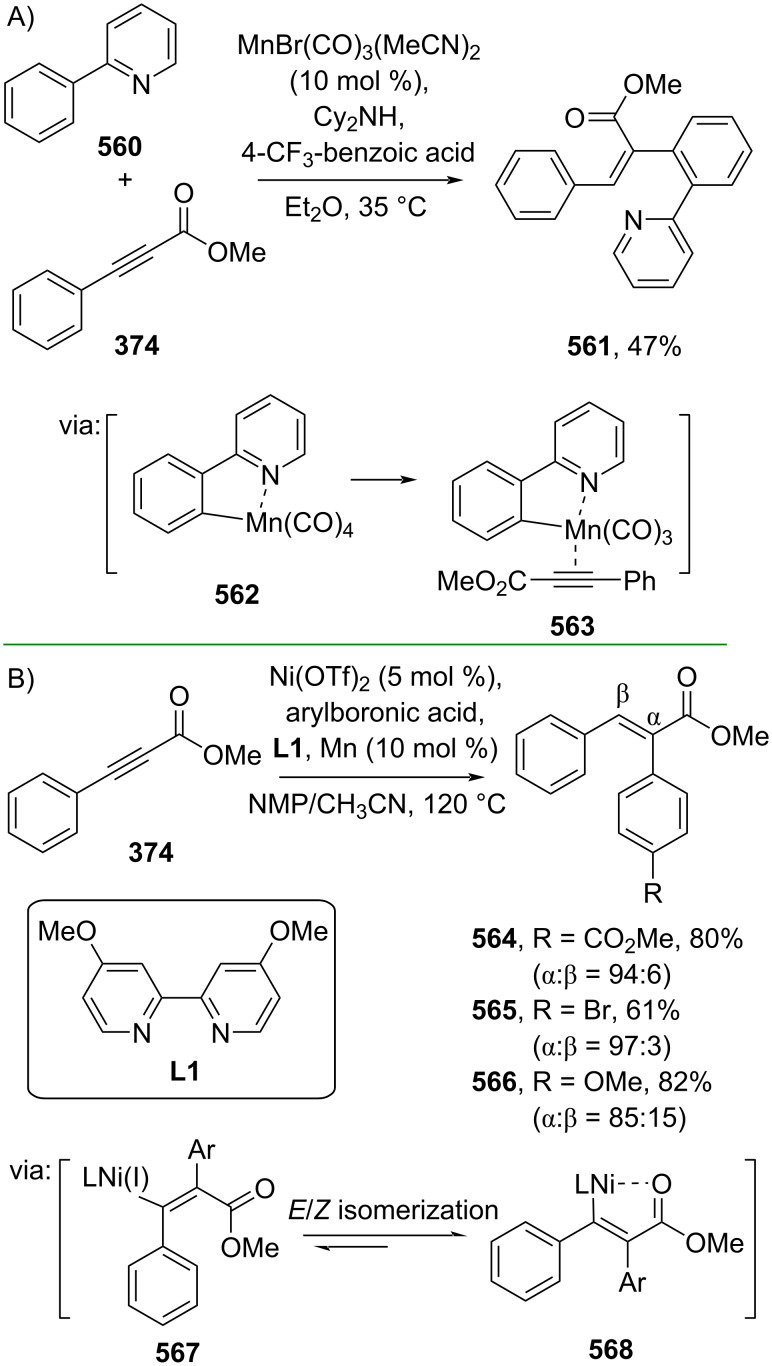
Earth-abundant transition-metal-catalyzed hydroarylation of α,β-alkynyl ester **374**.

On the other hand, earth-abundant transition metals, such as Mn and Ni, have also been applied to catalyze the hydroarylation of alkynes. In this context, Larrosa and co-workers (2022) utilized a Mn catalyst to achieve the hydroarylation of α,β-alkynyl ester **374** with 2-phenylpyridine (**560**) to afford the corresponding α-arylated cinnamate ester **561**. The reaction proceeds via pyridine–Mn coordination complex **562** as an off-cycle catalyst, subsequently leading to the active catalyst species **563** upon alkyne ligation (Scheme 107A) [181]. Recently, Wen and co-workers (2024) reported a Ni-catalyzed hydroarylation of α,β-alkynyl ester **374** with arylboronic acid to generate the corresponding (*E*)-α-arylated cinnamate esters **564**–**566** via formation of the active ArNi(I) species **567**, **568** after insertion into the alkynyl group (Scheme 107B) [182].

#### 3.3 Cβ functionalization

**Scheme 108:**
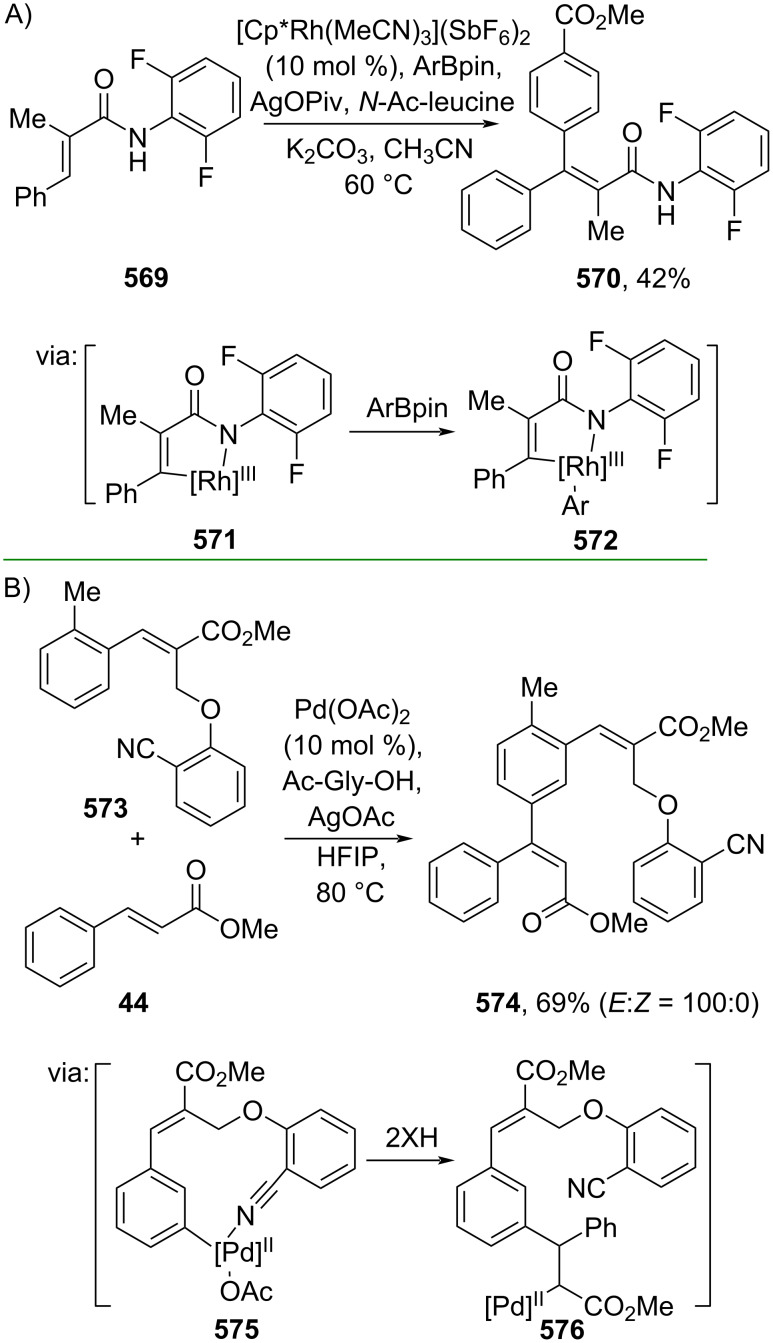
Precious transition-metal-catalyzed β-arylation of cinnamic acid amide/ester.

The direct Cβ-functionalization of cinnamic acid derivatives has also been studied in recent years, dominated by C–C/C–N cross-coupling methods. For instance, Lu and co-workers (2021) reported a Rh-catalyzed β-arylation of cinnamamide **569** with aryl pinacol boronates proceeding via an initial Rh–amidyl coordination triggering Cβ-activation to generate the five-membered rhodacycle species **571**, accompanied by transmetalation with the boronate to form **572** (Scheme 108A) [183]. In 2023, Bakthadoss and Reddy reported a Pd-catalyzed β-arylation of cinnamate ester **44** with an α-substituted cinnamate ester **573** as the coupling partner to give product **574** (Scheme 108B). In this study, the coupling reaction took place selectively at the *meta* position of **573** directed by the cyanide group, thus leading to the generation of a 12-membered palladacycle species **575** (Scheme 108B) [184].

**Scheme 109:**
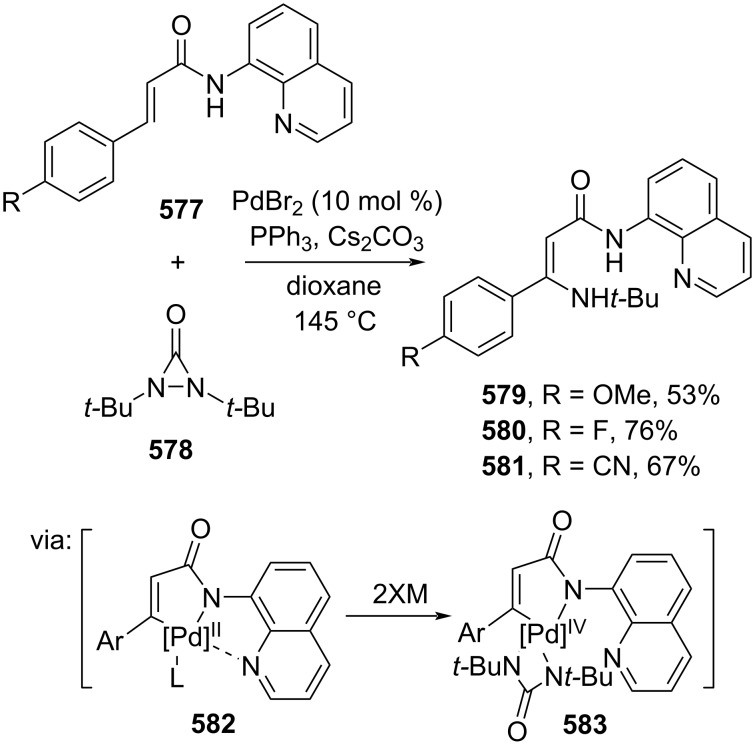
Pd-catalyzed β-amination of cinnamamide.

On the other hand, Shi and co-workers (2023) performed Cβ–N bond coupling of cinnamamide **577** with di-*tert*-butyldiaziridinone (**578**) catalyzed by Pd to give the corresponding β-amination products **579**–**581** (Scheme 109) [185]. The Cβ–H activation was driven by a ligand exchange with the cinnamamide **577** to form the palladacycle species **582**, which undergoes oxidative addition with di-*tert*-butyldiaziridinone (**583**).

**Scheme 110:**
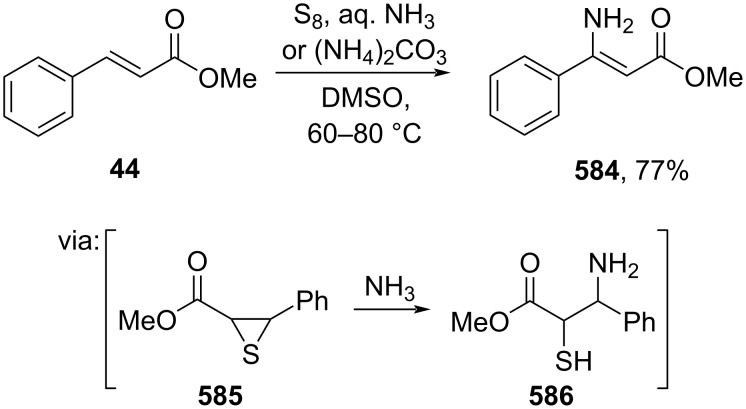
S_8_-mediated β-amination of methyl cinnamate (**44**).

In the absence of a metal catalyst, Phan and co-workers (2020) utilized S_8_ to mediate the β-amination of methyl cinnamate (**44**) via radical sulfuration of the cinnamate at the Cα-position (Scheme 110). Herein, a trisulfur radical anion reacted with the Cα/β of cinnamate to give a five-membered cyclic transition state followed by formation of thiirane **585** and subsequent NH_3_ attack at the Cβ position to give **586** [186].

**Scheme 111:**
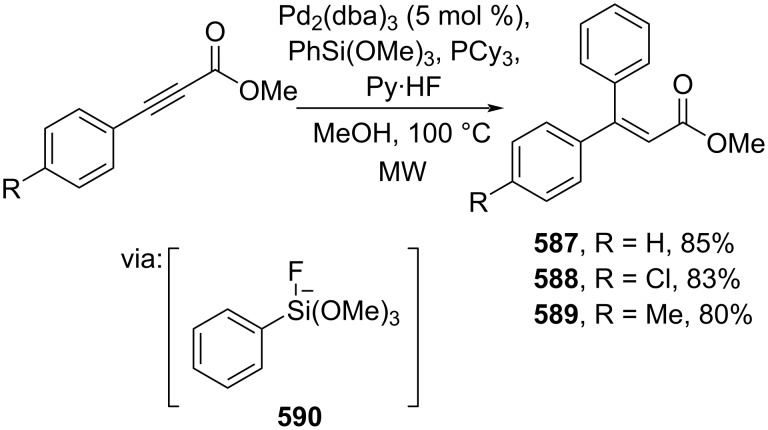
Pd-catalyzed cross-coupling reaction of alkynyl esters with phenylsilanes.

In addition to the electron-deficient alkenes discussed above, electron-deficient alkynes have also been used to prepare β-substituted cinnamic acid derivatives. Recently, Huang and co-workers (2024) investigated a Pd-catalyzed β-arylation of alkynyl esters with phenylsilanes as the coupling partner under microwave conditions to give the corresponding β-arylated cinnamate esters **587**–**589** (Scheme 111) [187]. Herein, the phenylsilane was activated by fluoride ions (**590**) and subsequently entered the cross-coupling reaction cycle.

**Scheme 112:**
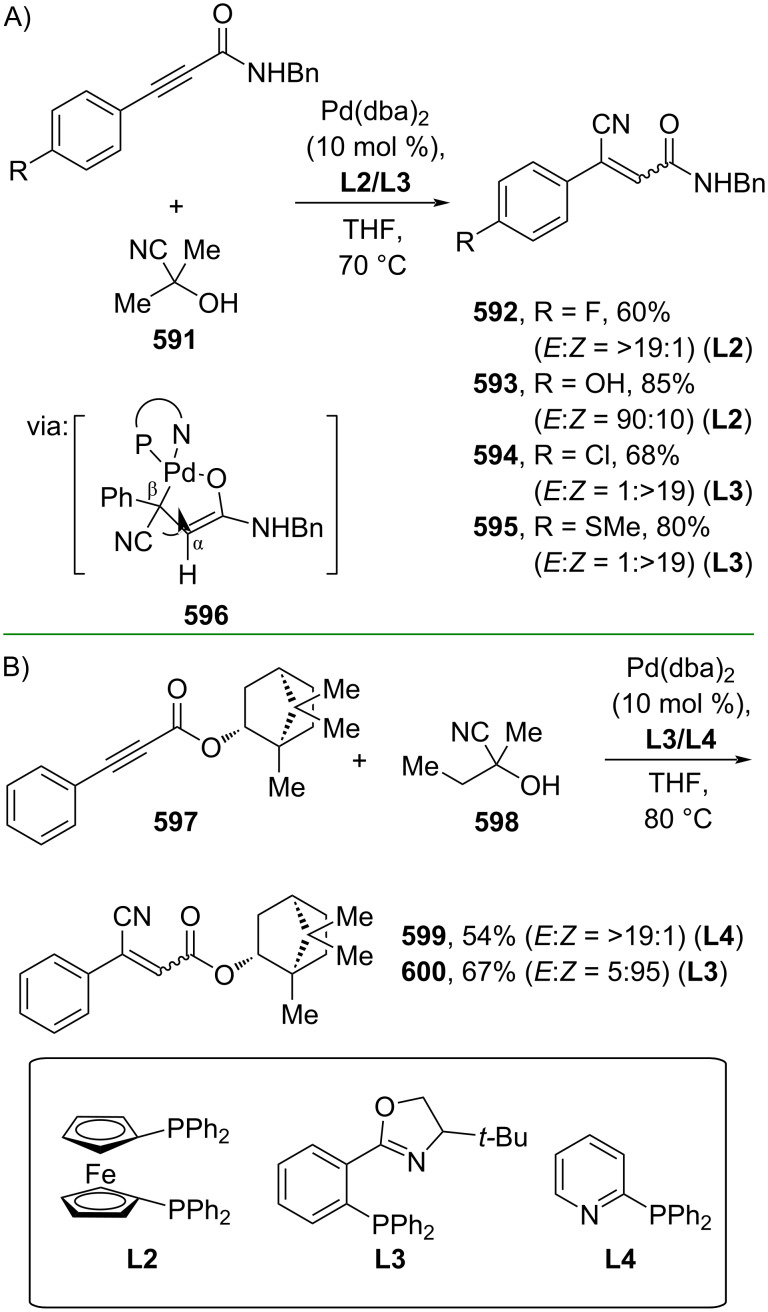
Pd-catalyzed β-cyanation of alkynyl amide/ester.

Fang and co-workers (2023) studied the Pd-catalyzed hydrocyanation of alkynyl amides using acetone cyanohydrin (**591**) as the cyanide source to give the corresponding β-cyanocinnamamides **592–595** with *E/Z*-selectivity depending on the ligand employed. The reaction proceeds via (*E*)-β-cyanocinnamamide as the intermediate for *Z*-isomer products (Scheme 112A) [188]. The *E*-to-*Z* isomerization occurred when Cα and Cβ rotated due to the steric effect promoted by the five-membered cyclopalladium ring in **596**. Recently, the same group also investigated the Pd-catalyzed hydrocyanation of alkynyl ester **597** using butanone cyanohydrin (**598**) as the cyanide reagent to generate the corresponding β-cyanocinnamate esters **599** and **600** with *E*/*Z*-selectivity depending on the ligand used (Scheme 112B) [189].

**Scheme 113:**
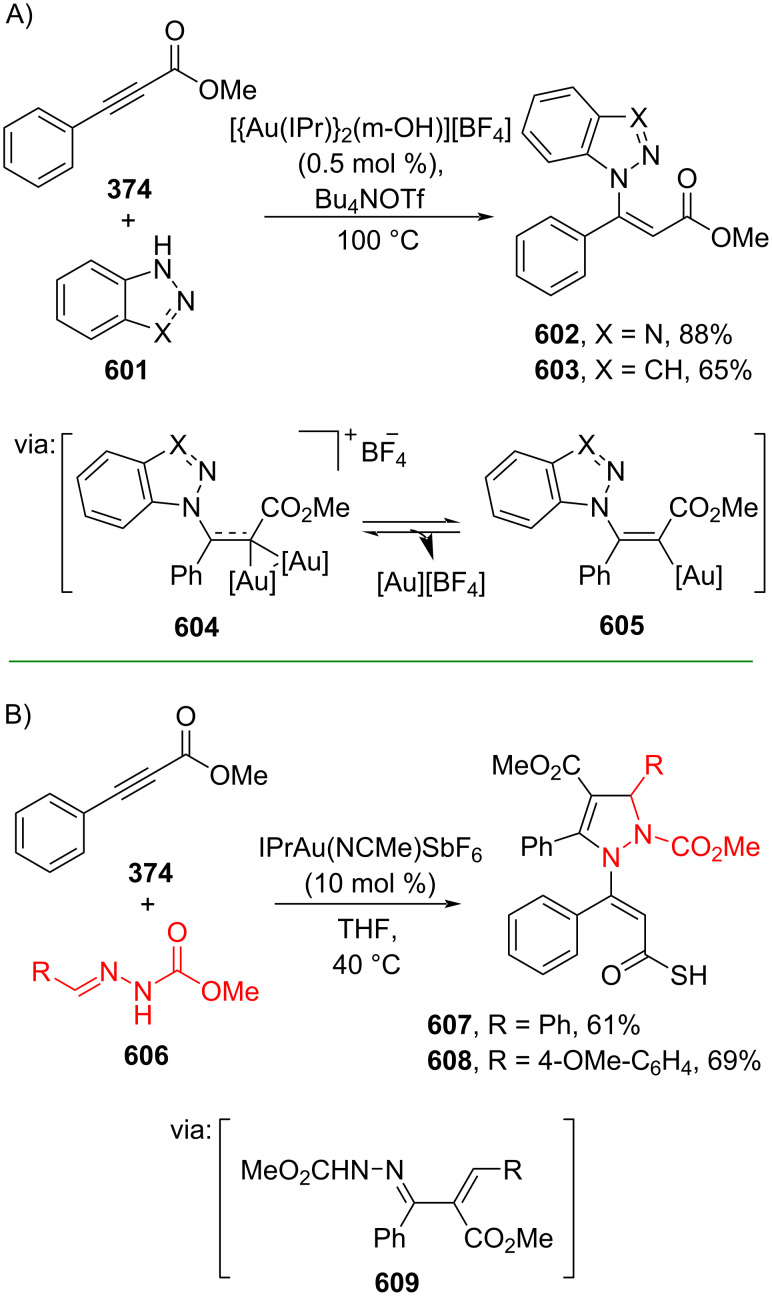
Au-catalyzed β-amination of alkynyl ester **374**.

Furthermore, Nolan and co-workers (2019) reported a Au-catalyzed regioselective hydroamination of alkynyl ester **374** and azole **601** to give the corresponding (*Z*)-β-substituted cinnamate esters **602**, **603** via the formation of a *gem*-diaurated species **604** equilibrated with s-monoaurated species **605** (Scheme 113A) [190]. Similarly, Matsuya and co-workers (2021) utilized Au(I) to catalyze the preparation of substituted pyrazolines **607** and **608** from alkynyl ester **374** and **606** via aza-enyne metathesis intermediate **609** followed by addition–6π-electrocyclization to afford the final product (Scheme 113B) [191].

**Scheme 114:**
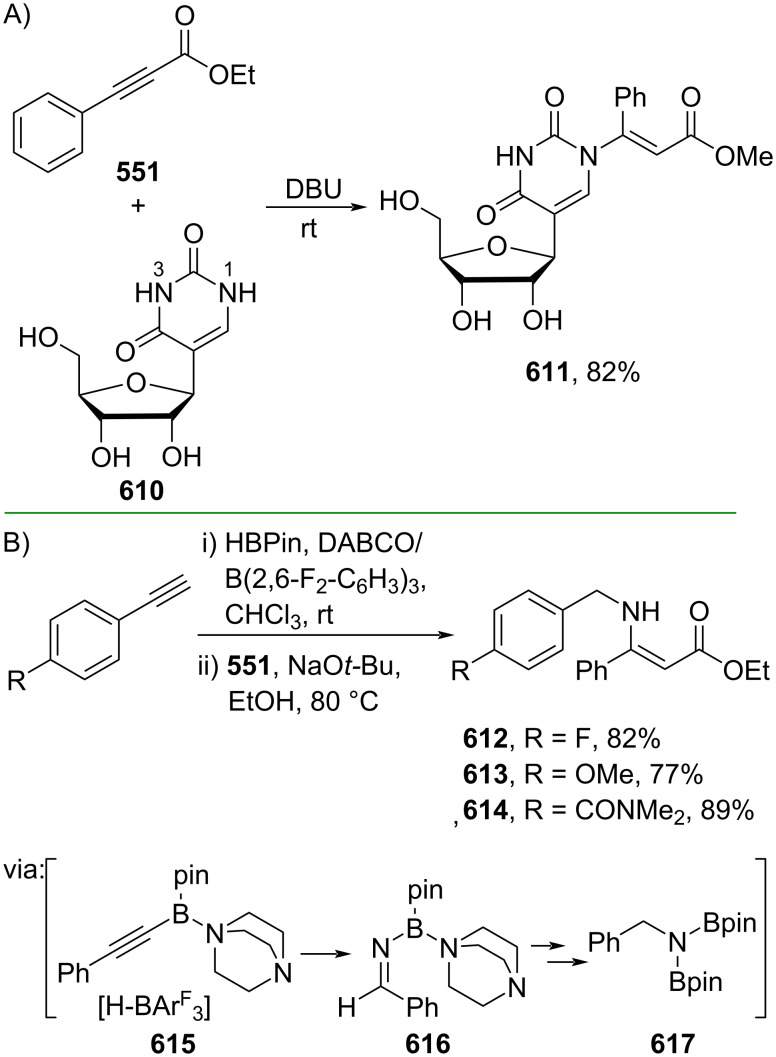
Metal-free-catalyzed Cβ-functionalizations of alkynyl esters.

In addition, a metal-free catalysis method was used to functionalize the Cβ position of electron-deficient alkynes, particularly through Michael addition. For instance, Kore and co-workers (2019) performed a Michael addition of alkynyl ester **551** with pseudouridine **610** using DBU as the base to furnish selective *N*1-acrylated product **611**, a useful building block for in vitro enzymatic introduction (Scheme 114A) [192]. The authors assumed that the bulkiness and non-nucleophilic nature of DBU played a major role in the observe N1- over N3-anion regioselectivity. On the other hand, Paradies and co-workers (2022) reported a frustrated Lewis pair-catalyzed hydroboration of nitriles to generate the nucleophilic diborylated amine **617**, followed by Cβ-addition of alkynyl ester **551** to give the final 3-amino acrylate products **612**–**614** in good yields (Scheme 114B) [193].

### 4 Aromatic group functionalization

#### 4.1 Cross-coupling reactions

The cross-coupling reaction is a versatile method for constructing various building blocks through C–C-bond formation, particularly aromatic group attachment, as demonstrated by its vast utilization in industrial setups and growing studies recorded over the years. Among other things, numerous improvements in the sustainability of the coupling methods, such as catalyst recyclability and waste reduction, have been the main focus of the development in this area. In addition, direct coupling reactions via C(sp^2^)–H activation using arenes instead of the common haloarenes have been developed with highly regioselective manners. Despite heavy dependency on rare transition metals utilization for the catalysts, many recent studies have demonstrated the application of more abundant metals, such as Ni, Co, and Cu. Herein, we selectively compile recent developments of cross-coupling reactions to access cinnamic acid derivatives in highly selective manners and diversely functional group-substituted aromatics with sustainability approaches.

**Scheme 115:**
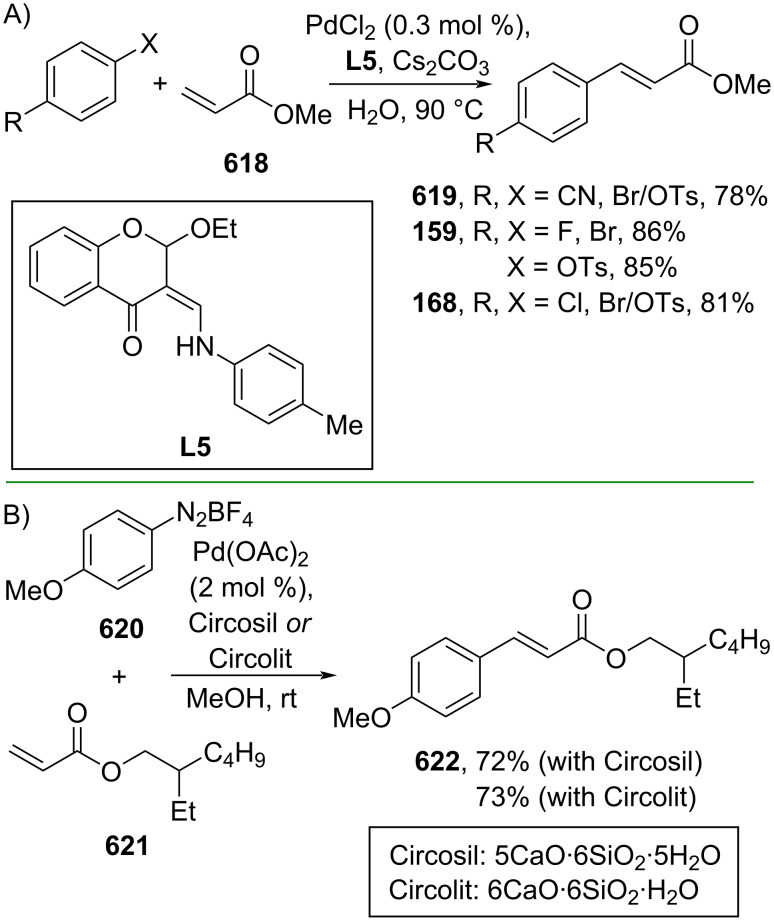
Heck-type reactions.

**4.1.1 C(sp^2^)–X alkenylation (X = halo, N_2_BF_4_, SO_2_NR_2_, CNR):** Khan and Parveen (2021) reported the palladium-catalyzed Mizoroki–Heck reaction of aryl halides/tosylates and methyl acrylate (**618**) in combination with the chroman-4-one-based ligand **L1** in water to give the corresponding methyl cinnamic acid derivatives **159**, **168**, and **619** (Scheme 115A) [194]. On the other hand, Brunner and Vedder (2019) utilized calcium-phyllosilicates (circosil/circolit) as the base for the Matsuda–Heck reaction of 4-methoxybenzenediazonium tetrafluoroborate (**620**) and the acrylate **621** to afford the commercial sunscreen incredient **622** in good yield (Scheme 115B) [195].

**Scheme 116:**
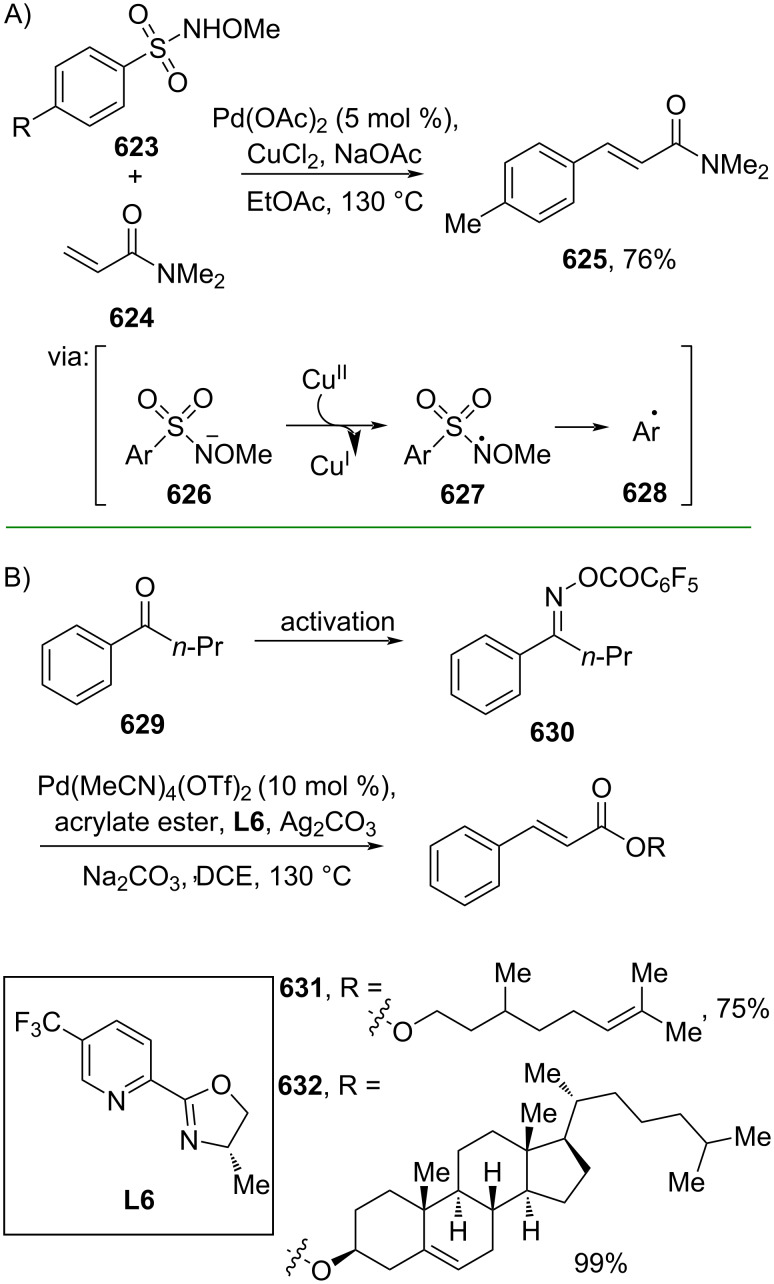
Mizoroki–Heck coupling reactions using unconventional functionalized arenes.

Several studies have explored non-conventional functionalized arenes as the aryl donor for Mizoroki–Heck coupling reactions. For instance, Panda and Ojha (2022) employed *N*-methoxysulfonamides **623** as aryl donor and acrylamide **624** to perform a palladium-catalyzed Mizoroki–Heck desulfitative arylation to give the corresponding cinnamamide **625**. The reaction was performed in the presence of CuCl_2_ as additive which is involved in a Cu-mediated SET mechanism (**626–628**) (Scheme 116A) [196]. Moreover, Dai and co-workers (2021) developed the Mizoroki–Heck reaction of aryl ketone **629** through C–C-bond cleavage (Scheme 116B) [197]. In this work, the aryl ketone **629** was converted into oxime ester **630** followed by Pd-catalyzed olefination to afford the corresponding cinnamate esters **631** and **632**. This method has been successfully scaled up to a gram-scale operation.

**Scheme 117:**
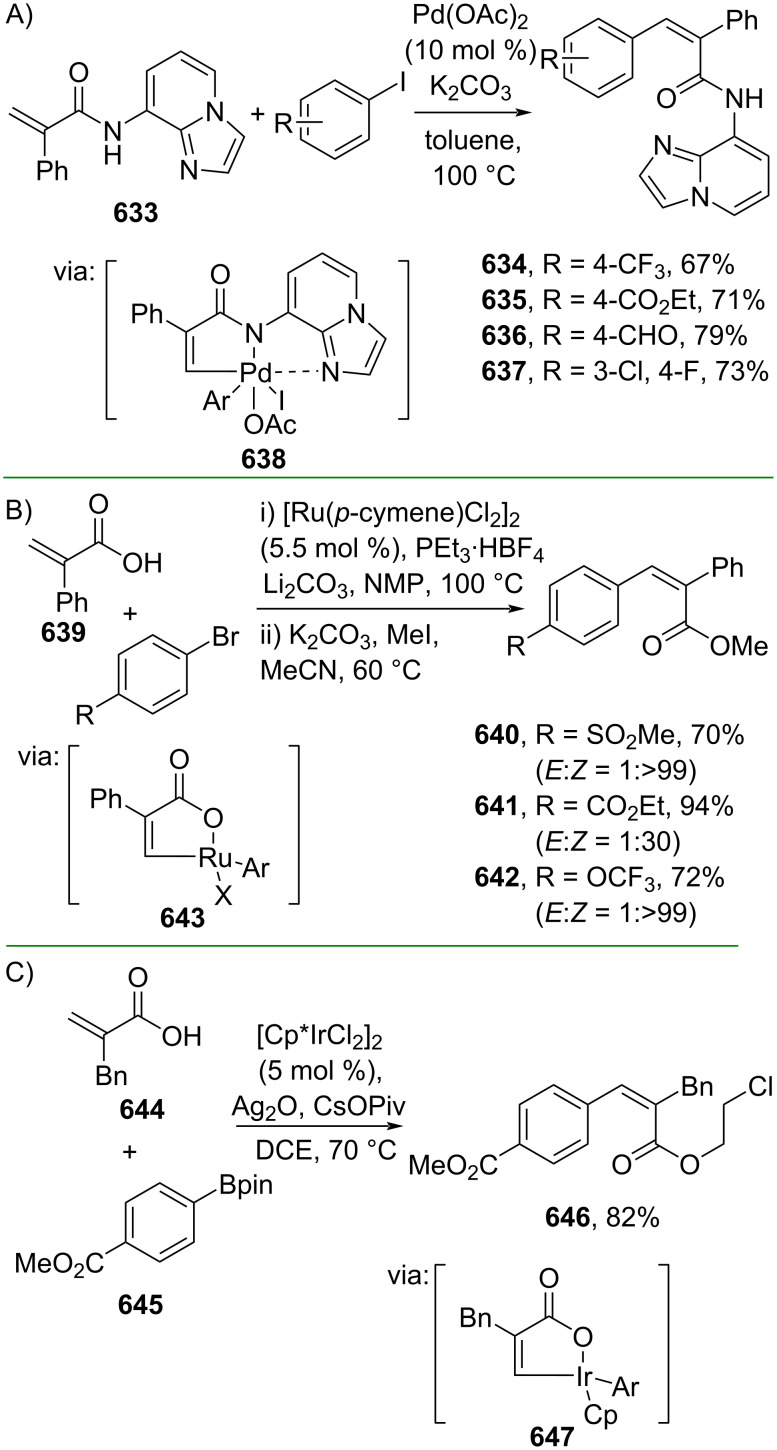
Functional group-directed Mizoroki–Heck coupling reactions.

Furthermore, (*Z*)-cinnamic acid derivatives could also be accessed through functional group-directed olefination of aryl halides. For example, Das and co-workers (2021) studied the Pd-catalyzed *Z*-selective arylation of acrylamide **633** using aminoimidazole[1,2-*a*]pyridine as the directing group (**638**) to give the corresponding (*Z*)-cinnamamides **634**–**637** (Scheme 117A) [198]. In addition, a gram-scale operation has been successfully conducted. Similarly, Gooßen and co-workers (2022) reported a Ru-catalyzed *Z*-selective arylation of acrylic acid **639** to obtain the corresponding methyl (*Z*)-cinnamates **640**–**642** after methylation (Scheme 117B) [199]. Herein, the stereoselectivity was directed by the -CO_2_H group to form metallacycle intermediate **643**. Wang and co-workers (2023) reported an Ir-catalyzed *Z*-selective arylation of acrylic acid **644** with arylboronate **645** to give the corresponding (*Z*)-cinnamate ester **646** following alkylation (Scheme 117C) [200].

**Scheme 118:**
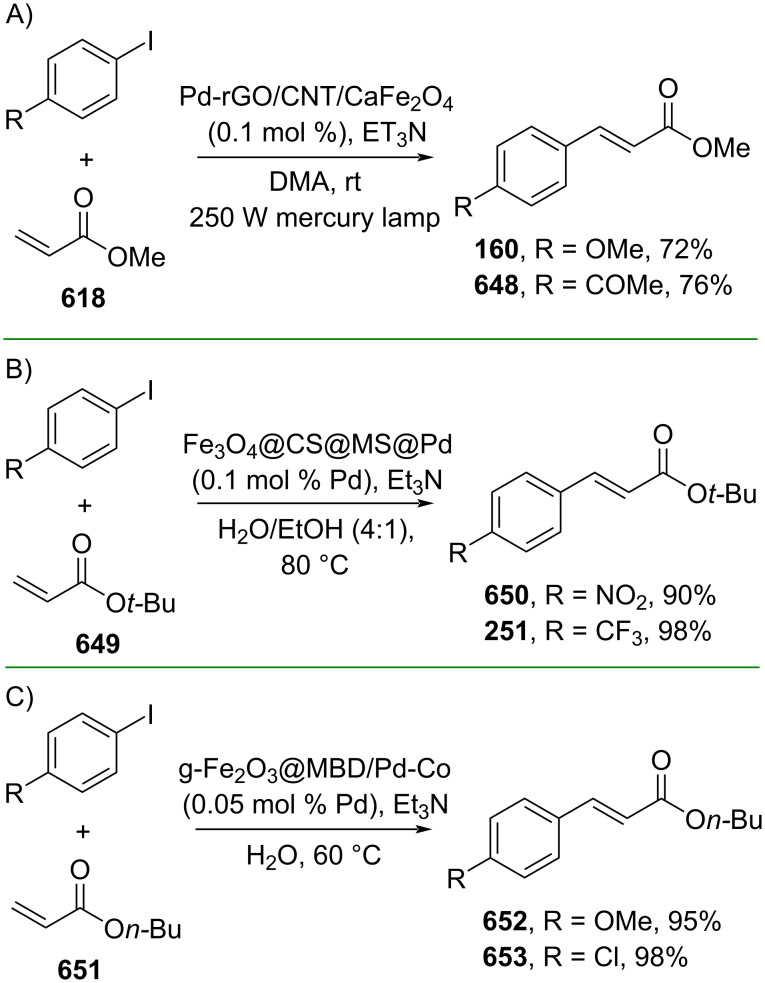
Pd nanoparticles-catalyzed Mizoroki–Heck coupling reactions.

Another attractive strategy employed material-supported Pd nanoparticles to catalyze the Mizoroki–Heck coupling reaction. This approach provided thermal stability, easy separation, and milder reaction conditions. For instance, Bagherzadeh and co-workers (2019) developed magnetic Pd-supported graphene oxide, carbon nanotube and ferrite (Pd-rGO/CNT/CaFe_2_O_4_) nanocomposites which were used as photocatalyst for the Mizoroki–Heck coupling reaction of aryl halides and methyl acrylate (**618**) to give the corresponding methyl cinnamates **160** and **648** (Scheme 118A) [201]. In addition, the catalyst could be recycled for up to six consecutive operations without significant activity loss. Similarly, Hasan (2020) prepared methyl salicylate-embedded magnetic chitosan-immobilized Pd nanoparticles (Fe_3_O_4_@CS@MS@Pd) to catalyze the Mizoroki–Heck coupling reaction of aryl halides and acrylate ester **649** in water to give the corresponding cinnamate esters **251** and **650** (Scheme 118B) [202]. Moreover, Sobhani and co-workers (2021) developed a Pd–Co bimetallic alloy immobilized on a melamine-based dendrimer on magnetic nanoparticles g-Fe_2_O_3_@MBD/Pd-Co and used it to catalyze the Mizoroki–Heck coupling reaction of aryl halides and acrylate ester **651** to obtain the corresponding cinnamate esters **652** and **653** (Scheme 118C) [203]. Similar studies utilizing Pd nanoparticles for Heck-type reactions have flourished in recent years [204–206].

**Scheme 119:**
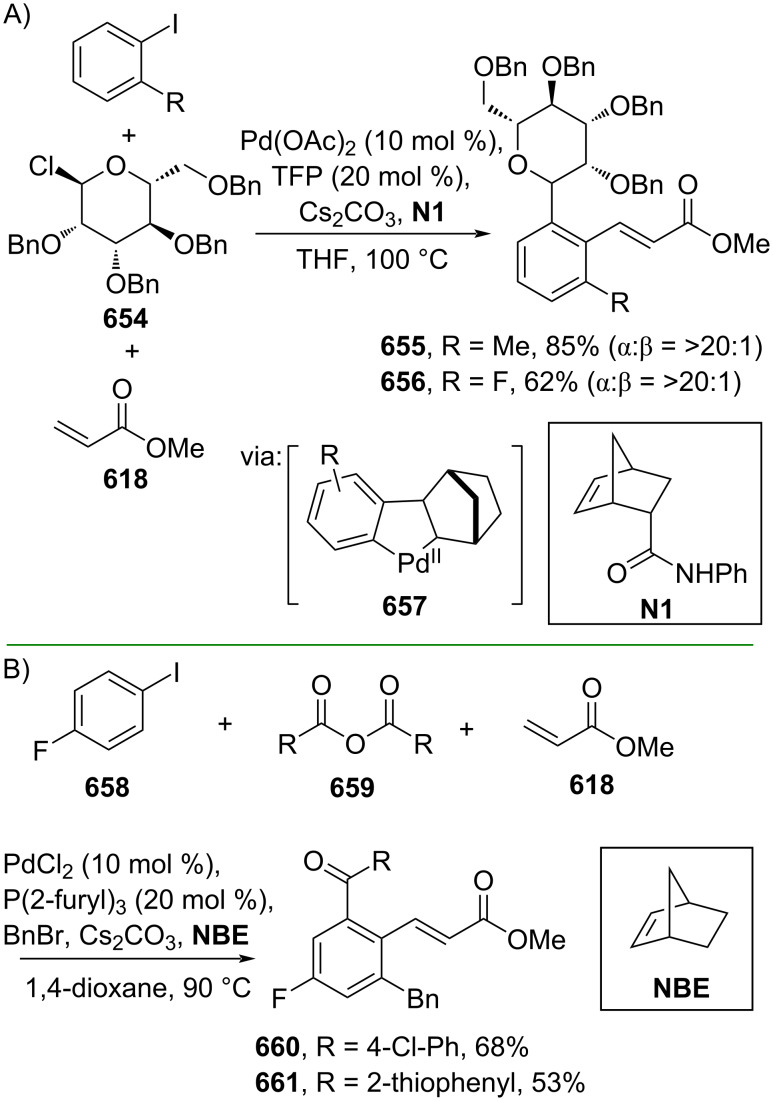
Catellani-type reactions to access methyl cinnamate with multifunctionalized arene.

Furthermore, multicomponent Heck-type reactions have been investigated to synthesize cinnamic acid derivatives with multifunctionalized arene core, particularly Catellani-type reactions. Catellani reactions allow multiple functional groups to be installed in the aryl ring in one pot. For instance, Cheng and co-workers (2020) reported the stereoselective synthesis of *C*-aryl glycosides using aryl iodides, tetrabenzyl-protected α-mannosyl chloride **654**, and methyl acrylate (**618**) through a Catellani mechanism (Scheme 119A) [207]. Herein, norbornenes **N1** are crucial to facilitate the multi-substitution of the arene ring through arylnorbornylpalladacycle **657** as the key species. In addition, a gram-scale operation has been successfully carried out. Similarly, Luan and co-workers (2020) conducted a four-component Catellani reaction using aryl iodide **658**, anhydrides **659**, acrylate ester **618**, and benzyl bromide to give the corresponding methyl cinnamates with multifunctionalized arenes **660** and **661** (Scheme 119B) [208].

**Scheme 120:**
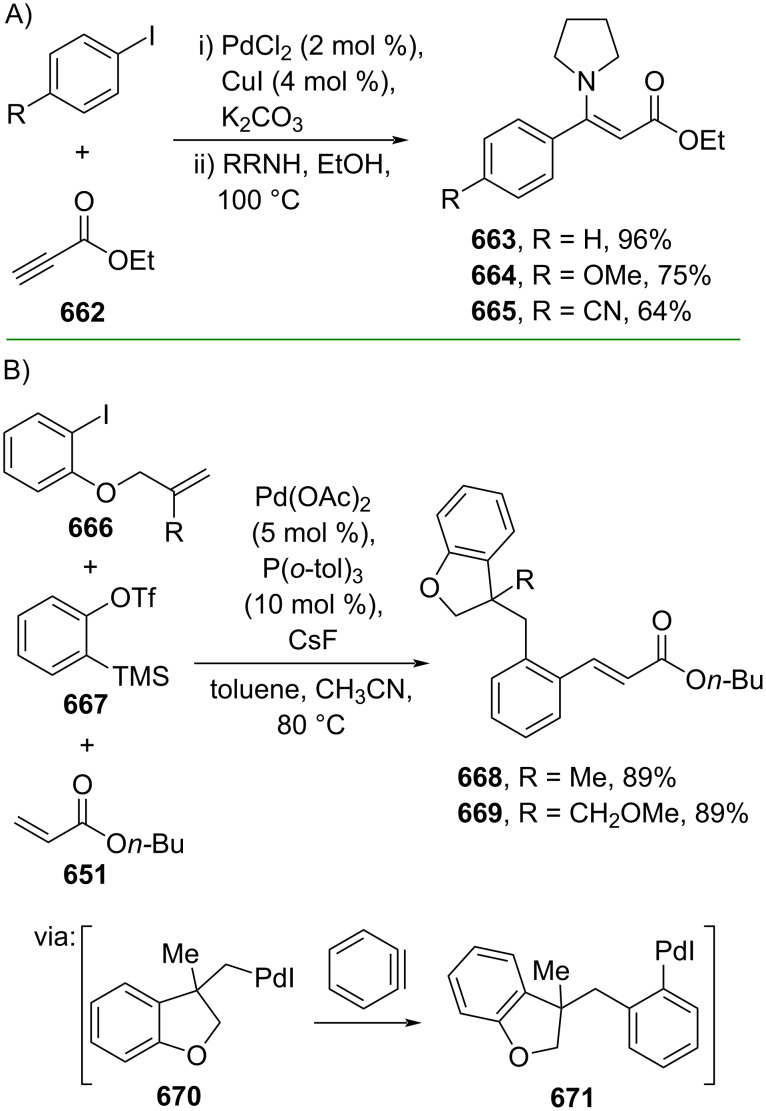
Multicomponent coupling reactions.

In addition, Müller and co-workers (2020) studied a three-component Sonogashira coupling reaction of aryl iodides, ethyl propiolate (**662**), followed by Michael addition of pyrrolidine to obtain β-amino enoates **663**–**665** (Scheme 120A) [209]. On the other hand, Yao and co-workers (2021) investigated a tandem intramolecular Heck coupling reaction/aryne dicarbofunctionalization (via **670** and **671**) of olefin-tethered aryl iodide **666**, aryne precursor **667**, and *n*-butyl acrylate (**651**) to give the corresponding *o*-alkylated arylacrylates **668** and **669** in excellent yields (Scheme 120B) [210]. As shown here, these multicomponent methods enabled the preparation of intricate and complex cinnamic acid derivatives more efficiently.

**Scheme 121:**
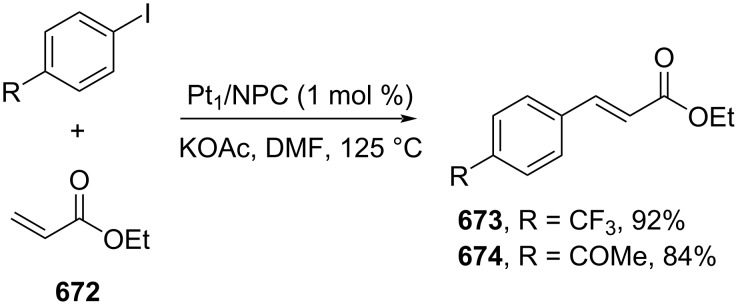
Single atom Pt-catalyzed Heck coupling reaction.

Müllen and co-workers (2023) reported a single atom Pt-catalyzed Heck coupling reaction of aryl iodides and ethyl acrylate (**672**) to give the corresponding cinnamate esters **673**, **674** (Scheme 121) [211]. The computational study revealed that a migratory insertion mechanism assisted by a base was operative.

**Scheme 122:**
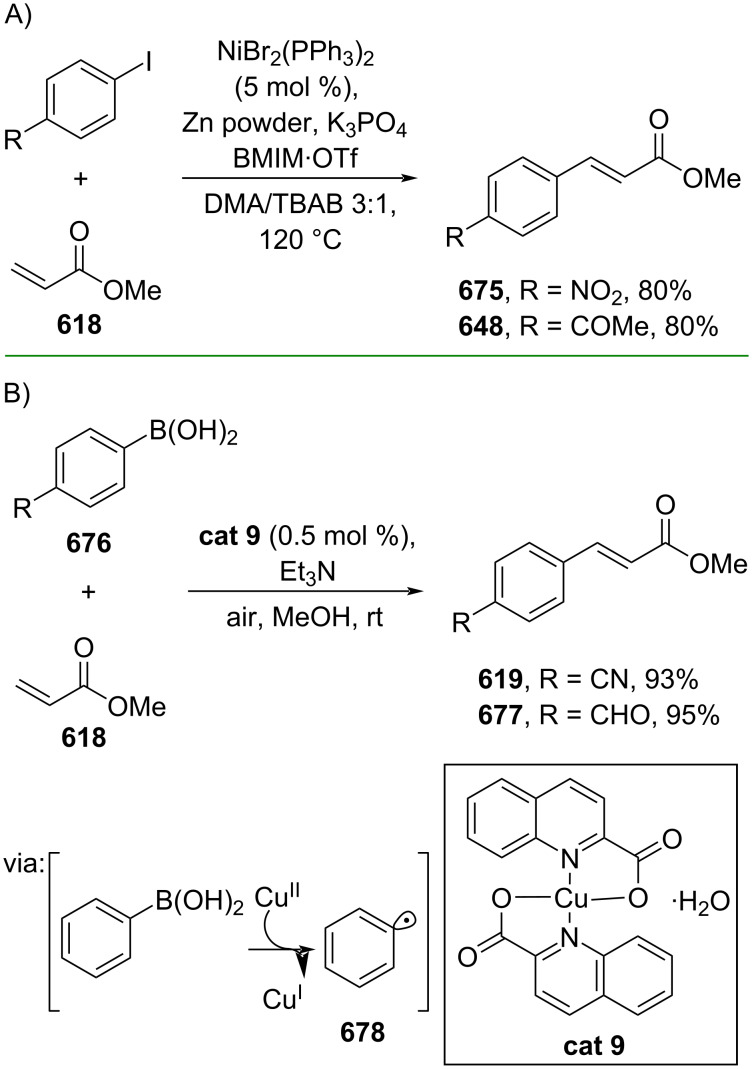
Earth-abundant transition metal-catalyzed Heck coupling reactions.

Despite massive dependency on rare transition metal catalysts applied for cross-coupling reactions, significant developments using earth-abundant transition metals have materialized in recent years to fulfill the sustainability agenda. For instance, Patel and co-workers (2019) utilized NiBr_2_(PPh_3_)_2_ to catalyze the Heck coupling reaction of aryl iodides and methyl acrylate (**618**) in the presence of Zn powder to obtain the corresponding methyl cinnamates **648** and **675** (Scheme 122A) [212]. The reducing agent (Zn) is necessary to recycle the Ni catalyst effectively. On the other hand, Chu and co-workers (2019) studied Mizoroki–Heck coupling reactions of arylboronic acids **676** and methyl acrylate (**618**) catalyzed by bis-quinoline-2-carboxylic acid Cu salt (**cat 9**) to give the corresponding methyl cinnamates **619**, **677** in excellent yields via phenyl radical **678** (Scheme 122B) [21]. In this study, air was used as the oxidant to recycle the catalyst.

**Scheme 123:**
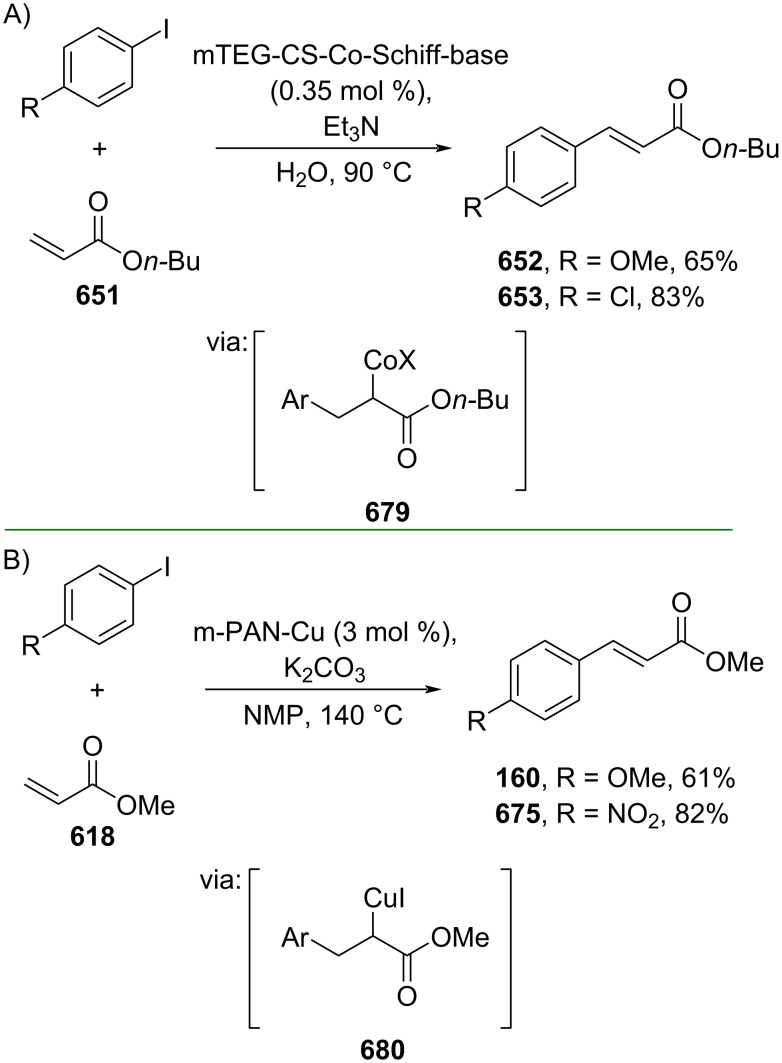
Polymer-coated earth-abundant transition metals-catalyzed Heck coupling reactions.

Furthermore, Sobhani and co-workers (2020) developed a hydrophilic heterogenous Co catalyst on chitosan (mTEG-CS-Co-Schiff-base) to catalyze Heck coupling reactions of aryl iodides and *n*-butyl acrylate (**651**) in water as environmentally benign solvent. The corresponding *n*-butyl cinnamates **652** and **653** were obtained in excellent yields via formation of the organocobalt species **679** (Scheme 123A) [213]. In addition, the catalyst could be successfully reused for up to 6 runs. On the other hand, Anas and co-workers (2020) reported Heck coupling reactions of aryl iodides and methyl acrylate **618** catalyzed by an amidoxime-modified PAN-immobilized Cu salt to give the corresponding methyl cinnamates **160** and **675** via the migratory insertion of Cu, generating organocopper species **680** (Scheme 123B) [214]. The catalyst was smoothly recycled for up to 5 runs without significant loss of its activity.

**Scheme 124:**
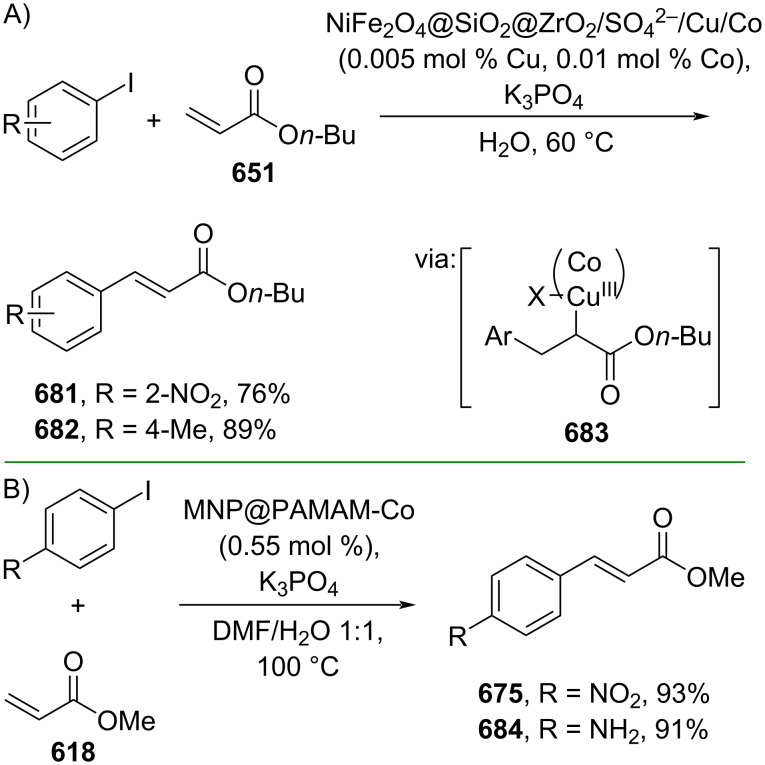
Earth-abundant transition-metal-based nanoparticles as catalysts for Heck coupling reactions.

Moreover, Alavi and co-workers (2021) prepared bimetallic nanoparticles supported on magnetic NiFe_2_O_4_ nanoparticles (NiFe_2_O_4_@SiO_2_@ZrO_2_/SO_4_^2–^/Cu/Co) to catalyze the Heck coupling reaction of aryl iodides and *n*-butyl acrylate (**651**) in water to afford the corresponding *n*-butyl cinnamates **681** and **682**. The catalyst could be smoothly recycled for up to 11 runs (Scheme 124A) [215]. The coupling mechanism was thought to proceed via migratory insertion of the Cu site to give an organocopper species **683**. Similarly, Koukabi and co-workers (2019) developed a polyamidoamine-Co complex immobilized on magnetic nanoparticles (MNP@PAMAM-Co) to catalyze Mizoroki–Heck coupling reactions of aryl iodides and methyl acrylate (**618**) giving rise to the corresponding methyl cinnamates **675**, **684** (Scheme 124B) [216]. The catalyst could be recycled for up to 5 operations without significant activity diminution.

**Scheme 125:**
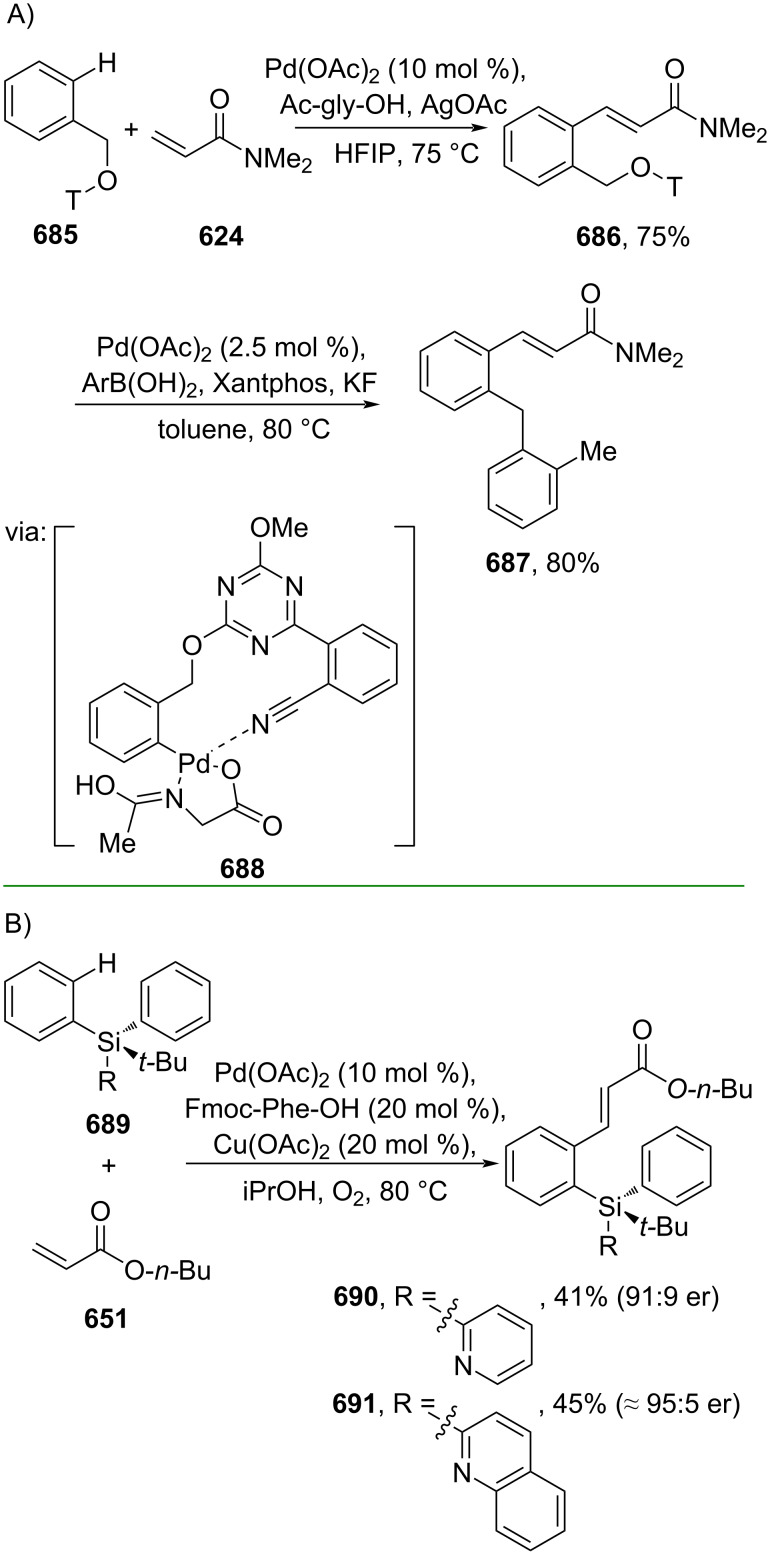
CN- and Si-based directing groups to access *o*-selective cinnamic acid derivatives.

**4.1.2 C(sp^2^)–H alkenylation:** The direct C(sp^2^)–H alkenylation of arenes has significantly flourished as the need to use or prepare haloarenes or heteroatom-substituted arenes involved in the coupling process diminishes, thus promoting reaction efficiency and atom economy. However, due to the weaker activity of the C(sp^2^)–H bond than its heteroatom-functionalized counterparts, its application was hampered by its low reactivity and poor regioselectivity. In this case, utilizing directing group-tethered arenes could overcome this issue elegantly. For instance, Jin and co-workers (2019) developed consecutive activation of *ortho*-C(sp^2^)–H bond and *ipso*-C(sp^3^)–O bond utilizing a nitrile-based directing group via CN–[Pd] coordination (**688**). In this study, a directing group-functionalized arene **685** was reacted with acrylamide **624** to give the corresponding *ortho*-selective substituted cinnamamide **686** followed by an *ipso*-C(sp^3^)–O arylation to obtain the final cinnamamide product **687** in good yield (Scheme 125A) [217]. Similarly, Xu and co-workers (2019) utilized a silane-functionalized arene **689** and reacted it with acrylate **651** to conduct an *ortho*-selective C(sp^2^)–H alkenylation to obtain the corresponding cinnamate esters **690** and **691** with good enantiomeric ratio (Scheme 125B) [218]. In addition, the reaction scale could be raised to a gram-scale operation.

**Scheme 126:**
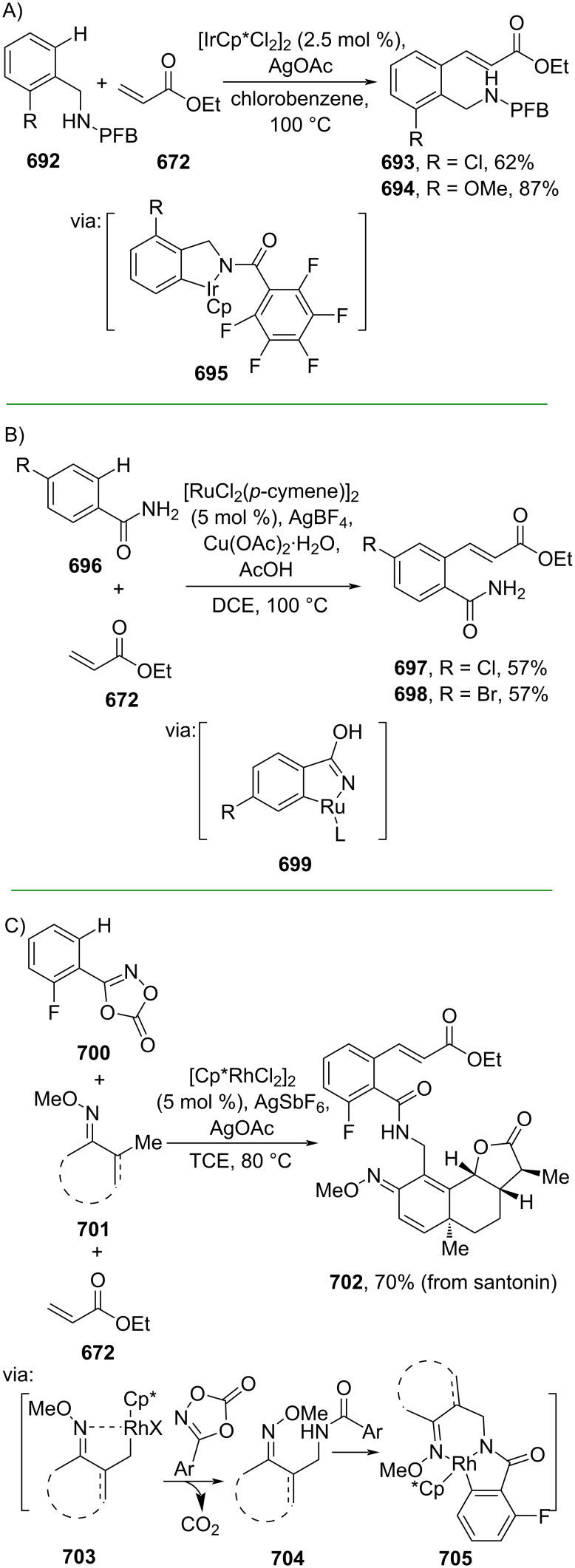
Amide-based directing group to access *o*-selective cinnamic acid derivatives.

Fu and co-workers (2019) employed Ir to catalyze the *o*-selective C(sp^2^)–H alkenylation of 2,3,4,5,6-pentafluorobenzoyl (PFB)-anchored benzylamine **692** using silver acetate as oxidant to obtain the corresponding *o*-substituted ethyl cinnamates **693** and **694** via a five-membered iridacycle **695** (Scheme 126A) [219]. On the other hand, Kumar and co-workers (2021) investigated the Ru-catalyzed *o*-selective C(sp^2^)–H alkenylation of benzamide **696** in the presence of copper acetate as oxidant to give the corresponding *o*-substituted ethyl cinnamates **697**, **698** (Scheme 126B) [220]. The amide group directed the Ru catalyst to activate *o*-C(sp^2^)–H by forming a five-membered ruthenacycle **699**. In addition, a gram-scale operation has been successfully achieved. Similarly, Yang and co-workers (2023) reported a Rh-catalyzed multicomponent coupling reaction using natural products-based fragments via dual C–H activation to generate the corresponding ketoxime **702** in good yield (Scheme 126C) [221]. Initially, the Rh catalyst activates the C(sp^3^)–H bond in oxime **701** enabling C–C bond formation with dioxazolone **700** (**703** and **704**). The newly installed amide group in intermediate **704**, then acts as directing group for the Rh catalyst to activate the *o*-C(sp^2^)–H bond of the arene by forming metalacycle species **705**.

**Scheme 127:**
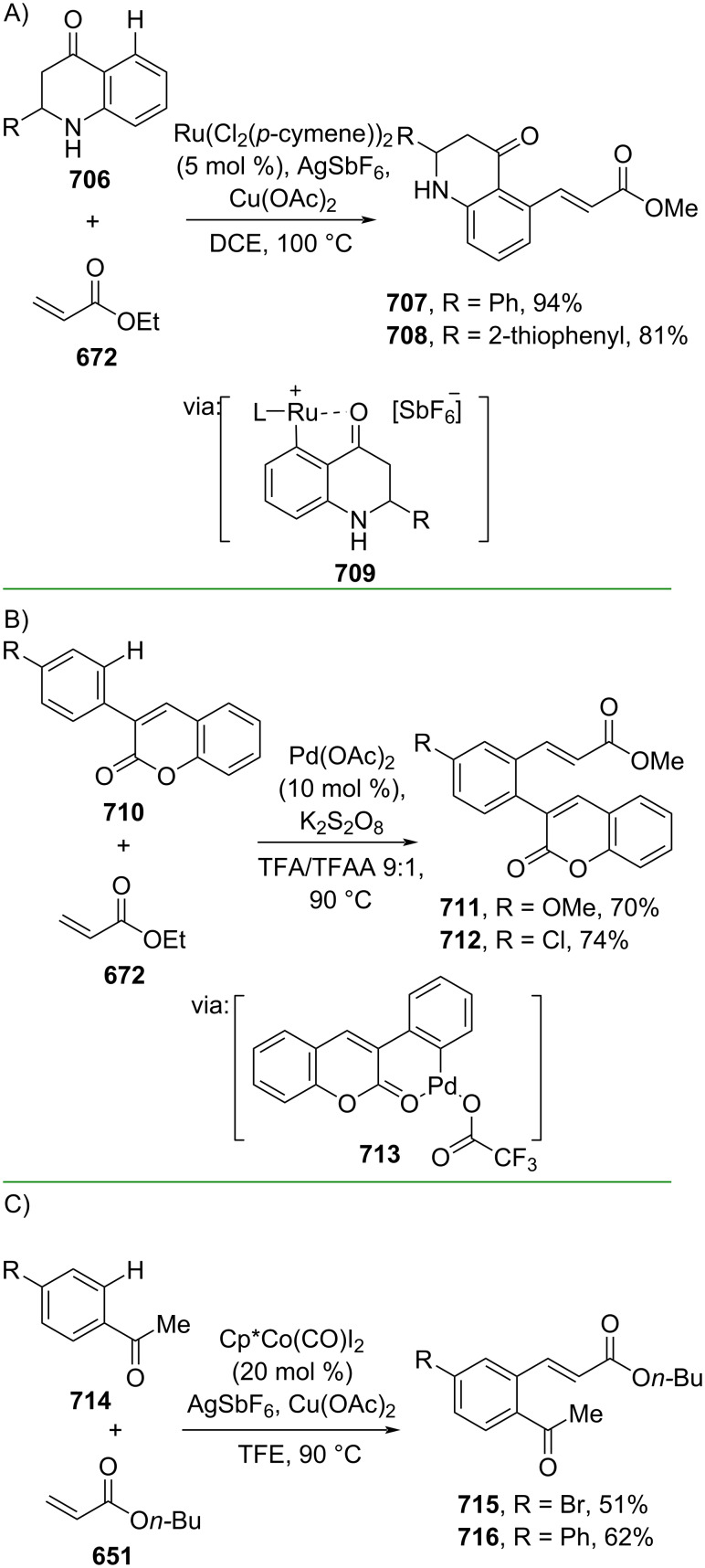
Carbonyl-based directing group to access *o*-selective cinnamic acid derivatives.

Beyond *N*-based directing groups, C=O groups could also direct C–H functionalization. For example, Bakthadoss and co-workers (2020) employed Ru to catalyze the *o*-C(sp^2^)–H alkenylation of azaflavanone **706** to give the corresponding methyl cinnamates **707** and **708** via C=O–[Ru] directing mode **709** (Scheme 127A) [222]. Similarly, Kumar and co-workers (2021) reported weakly chelating lactone-directed *o*-C(sp^2^)–H alkenylation (via **713**) of 3-arylcoumarins **710** and acrylate **672** to afford the corresponding methyl cinnamates **711** and **712** (Scheme 127B) [223]. In addition, a gram-scale operation has been successfully conducted.

However, the direct *o*-selective C(sp^2^)–H alkenylation catalyzed by earth-abundant transition metals is still extremely underdeveloped. One rare example was reported by Maji and co-workers in 2019. In this work, aromatic ketones **714** were selectively *o*-alkenylated catalyzed by Co(III) to give the corresponding *o*-ketocinnamate esters **715** and **716** in good yields via metallacycle species (Scheme 127C) [224].

**Scheme 128:**
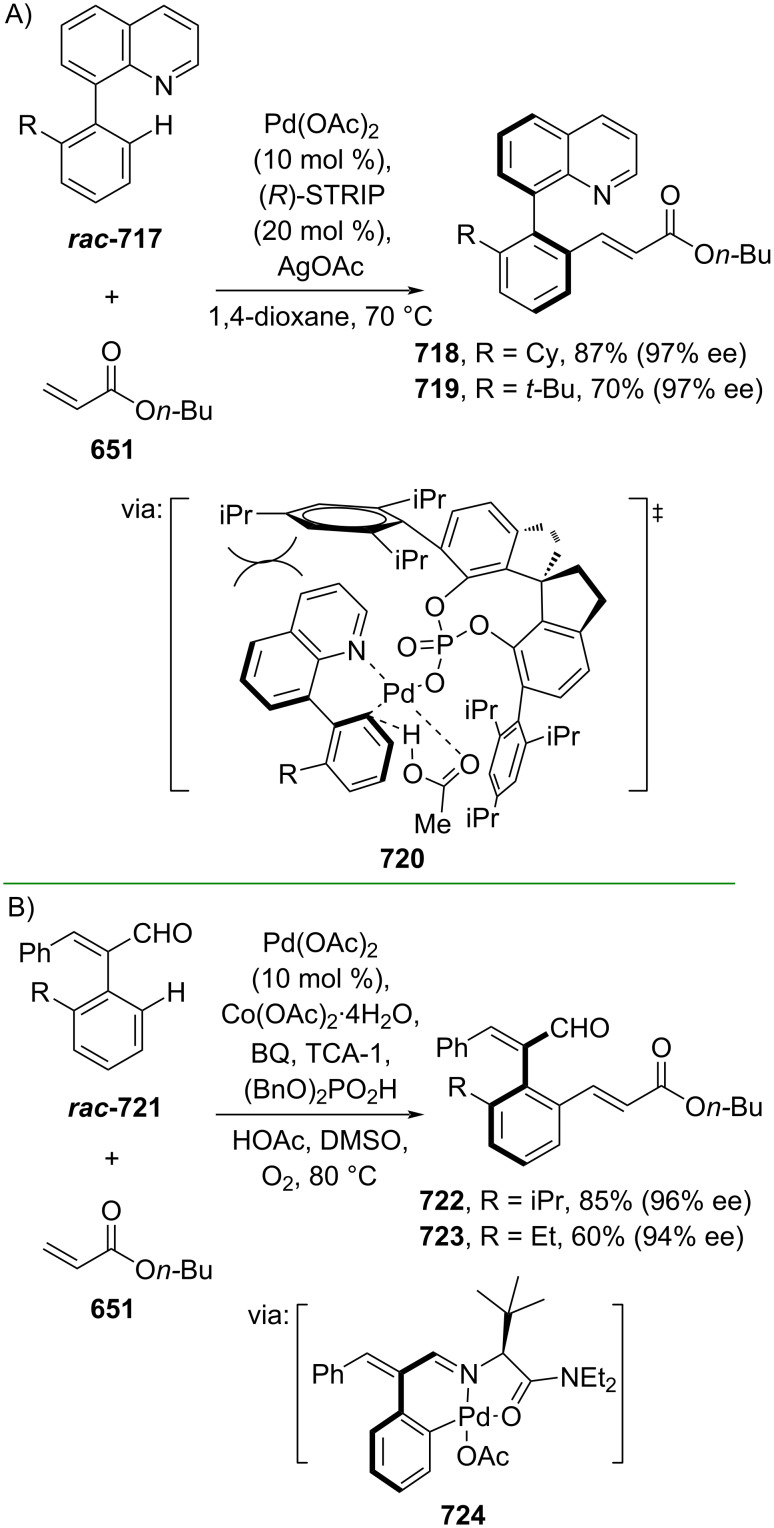
Stereoselective preparation of atropisomers via *o*-selective C(sp^2^)–H functionalization.

Numerous atropisomeric compounds are known for their bioactivities. Significant efforts have been dedicated to the stereoselective synthesis of atropisomers, by, for example, selective C–H functionalization and the *ortho*-selective C(sp^2^)–H functionalization is a powerful tool for achieving this goal. Shi and co-workers (2019) developed a Pd-catalyzed C(sp^2^)–H alkenylation of *rac*-quinoline-derived biaryl **717** with acrylate **651** to give the corresponding axially chiral cinnamate ester-anchored biaryls **718** and **719** with excellent enantiomeric excess utilizing chiral spiro phosphoric acids ((*R*)-STRIP) as ligands (Scheme 128A) [225]. DFT calculations revealed a concerted metalation–deprotonation (CMD)-type transition state of Spinol-ts-*S* **720** as the main pathway due to the less steric repulsion between quinolone and isopropyl groups. The same group (2019) also reported the preparation of axially chiral styrenes **722**, **723** from arenes **721** and a chiral auxiliary *tert*-leucine-derived amino amide via a palladacycle species **724** (Scheme 128B) [226]. In addition, the method was used for a gram-scale operation.

**Scheme 129:**
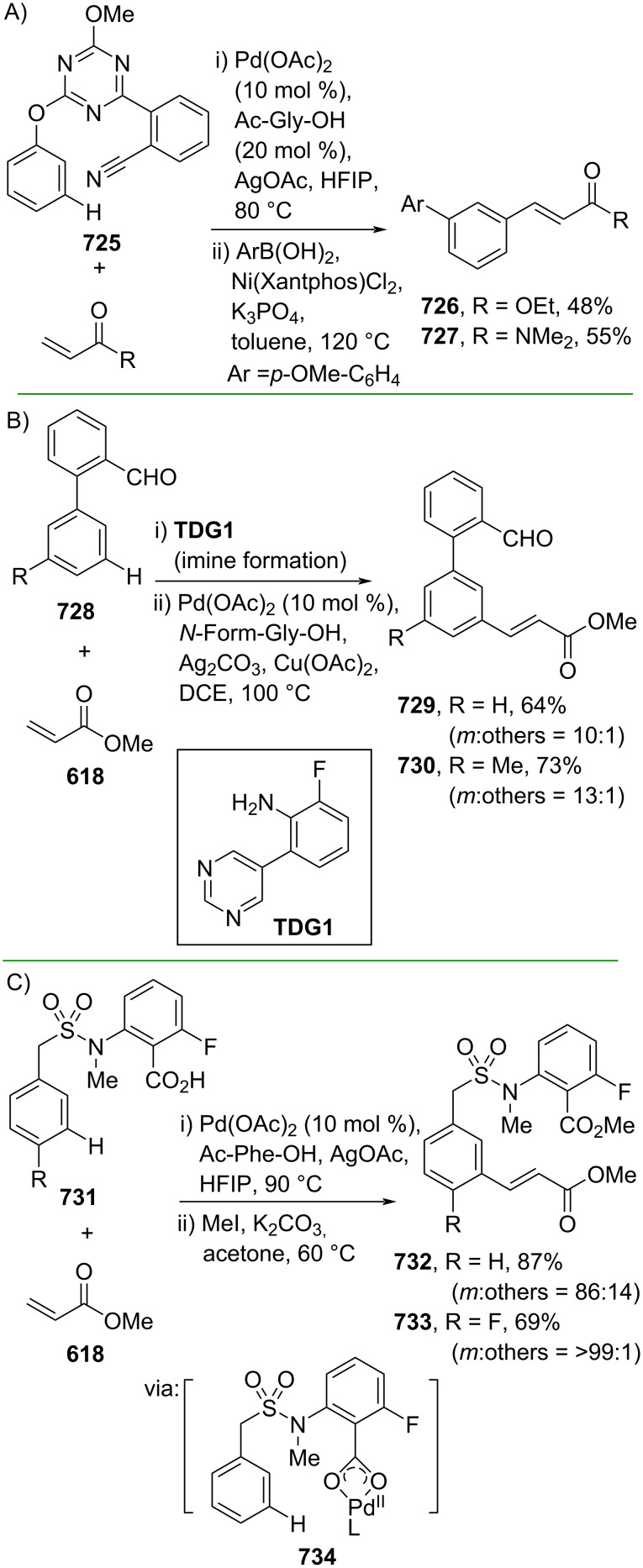
*meta*-Selective C(sp^2^)–H functionalization using directing group-tethered arenes.

On the other hand, *meta*-selective C(sp^2^)–H alkenylation reactions to access *m*-substituted cinnamic acid derivatives using directing groups are considerably less explored than their *ortho*-counterparts due to more remote activations. In this context, the formation of a thermodynamically stable, larger metallacycle during C–H activation was the key to achieving *m*-selectivity. Despite the challenges, several studies have been published in recent years to access *meta*-position functionalizations. Yu and co-workers (2019) investigated a one-pot Pd-catalyzed *m*-selective alkenylation/*ipso*-selective C(sp^2^)–O functionalization of directing group-tethered phenols **725** to give the corresponding *m*-functionalized cinnamate esters **726**, **727** in moderate yields (Scheme 129A) [227]. The nitrile-tethered template facilitated remote C(sp^2^)–H activation via a macrocyclic palladacycle species. Similarly, Maiti and co-workers (2021) successfully achieved *m*-selective C(sp^2^)–H functionalization of biphenyl aldehydes **728** to afford the corresponding *m*-arylated cinnamate esters **729** and **730** using a pyrimidine-based directing group (TDG1) via a macrocyclic C=N–[Pd] species (Scheme 129B) [228]. Moreover, Li and co-workers (2020) utilized carboxyl group-tethered benzylsulfonamides **731** to access *m*-sulfamoylmethyl cinnamate esters **732** and **733** via κ^2^ coordination (**734**) of the pendant carboxyl group with the Pd catalyst (Scheme 129C) [229].

**Scheme 130:**
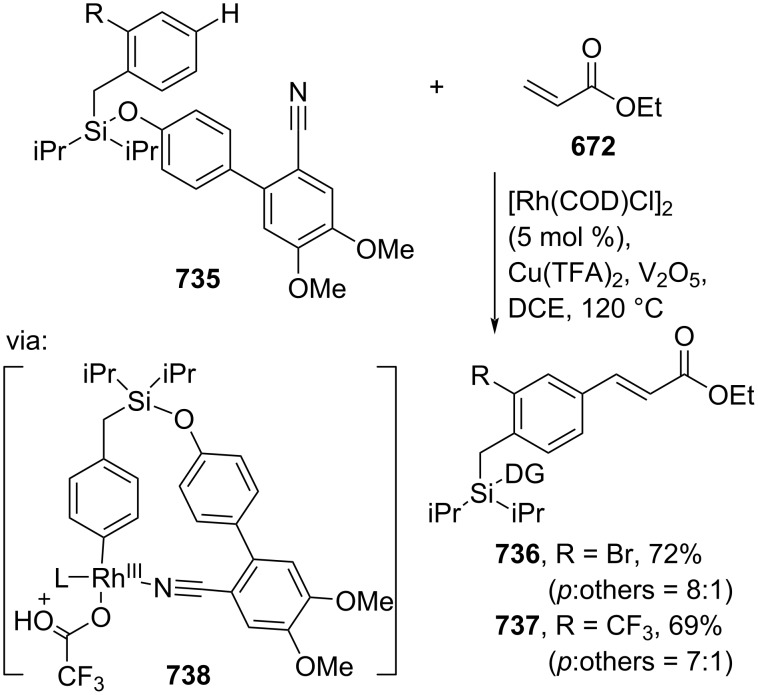
*para*-Selective C(sp^2^)–H functionalization using directing group-tethered arenes.

Furthermore, Maiti and co-workers (2019) studied the first distal *para*-selective C(sp^2^)–H alkenylation of silyl-tethered arenes **735** to afford the corresponding *p*-silylmethyl cinnamate esters **736** and **737** via remote CN–[Rh] coordination (**738**) (Scheme 130) [230].

**Scheme 131:**
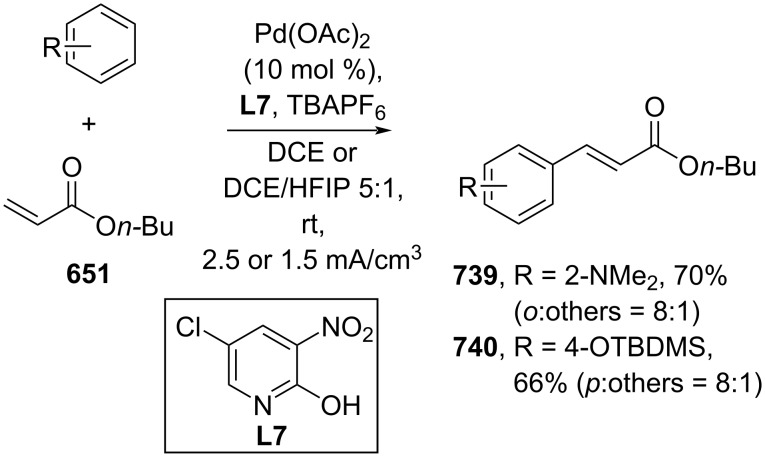
Non-directed C(sp^2^)–H functionalization via electrooxidative Fujiwara–Moritani reaction.

The same group (2022) also reported a non-directed C(sp^2^)–H functionalization of arenes through an electrooxidative Fujiwara–Moritani reaction to obtain the corresponding cinnamate esters **739** and **740** with high regioselectivity via a Pd^II^/Pd^IV^ cycle (Scheme 131) [231]. In addition, a gram-scale operation has been successfully achieved.

#### 4.2 Miscellaneous reactions

**Scheme 132:**
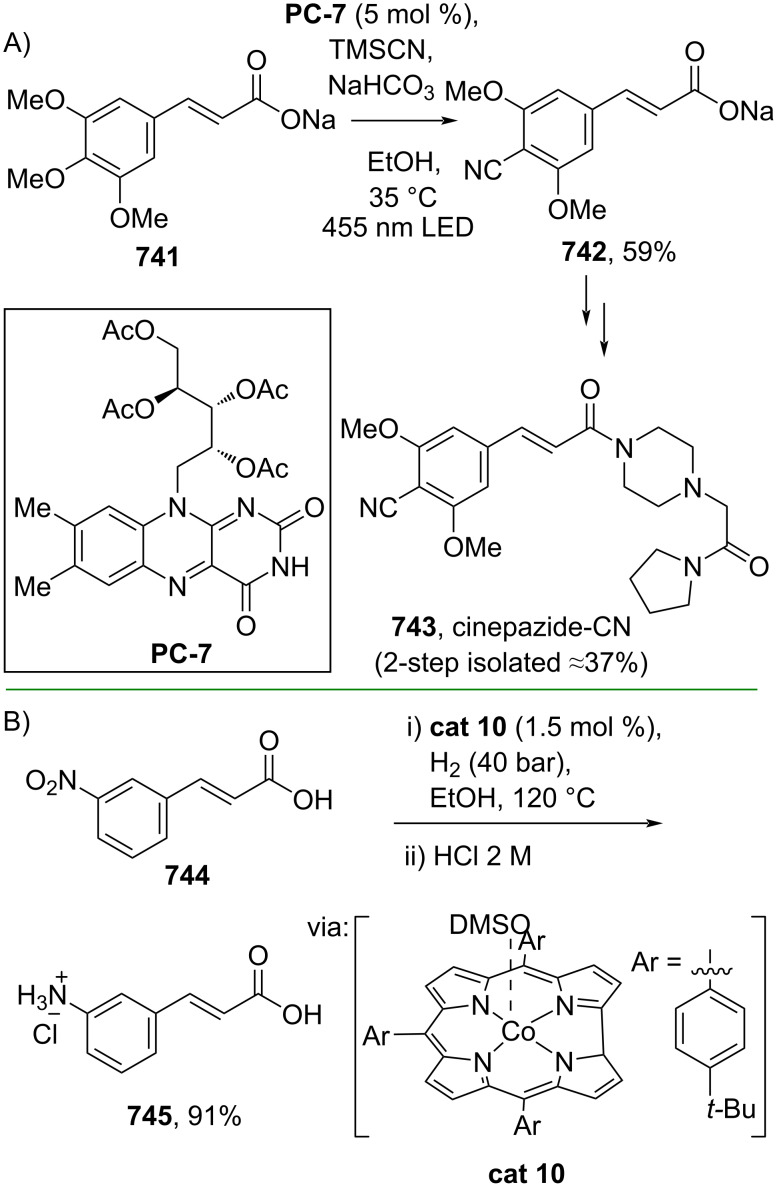
Interconversion of functional groups attached to cinnamic acid.

Li and co-workers (2023) developed a photocatalyzed cyanation of *p*-methoxycinnamate salt **741** utilizing TMSCN as the cyanide source and photocatalyst **PC7** to give the corresponding *p*-cyano product **742** in moderate yield via an arene cation radical species (Scheme 132A) [232]. The product **742** was then converted into a nitrile analog of cinepazide **743**, a drug used for treating cardio- and cerebrovascular diseases, in ≈37% isolated yield over two steps. On the other hand, Topf and co-workers (2021) utilized Co corrole (**cat 10**) to catalyze the hydrogenation of *m*-nitrocinnamic acid (**744**) to afford the HCl salt of *m*-aminocinnamic acid **745** upon acidification in excellent yield (Scheme 132B) [233].

**Scheme 133:**
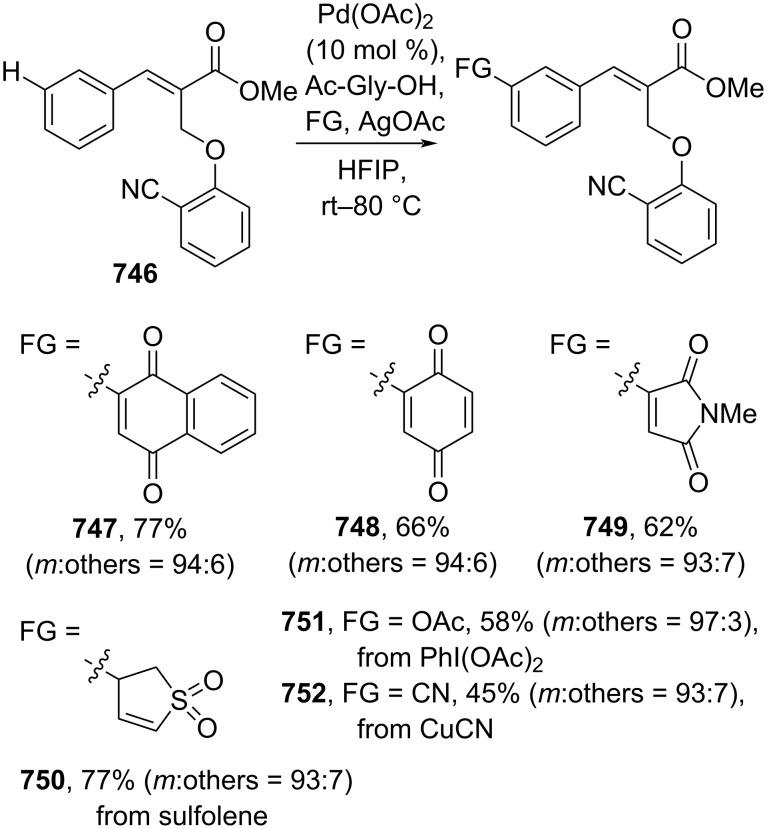
*meta*-Selective C(sp^2^)–H functionalization of cinnamate ester.

Moreover, a Pd-catalyzed direct C(sp^2^)–H functionalization of cinnamic acid derivatives has been recently reported. Bakthadoss and Reddy (2023) developed a protocol to functionalize the *meta*-position of a nitrile-based directing group-anchored cinnamate ester **746** with various functional groups to the corresponding *m*-substituted cinnamate esters **747–752** in moderate to good yields (Scheme 133) [184].

**Scheme 134:**
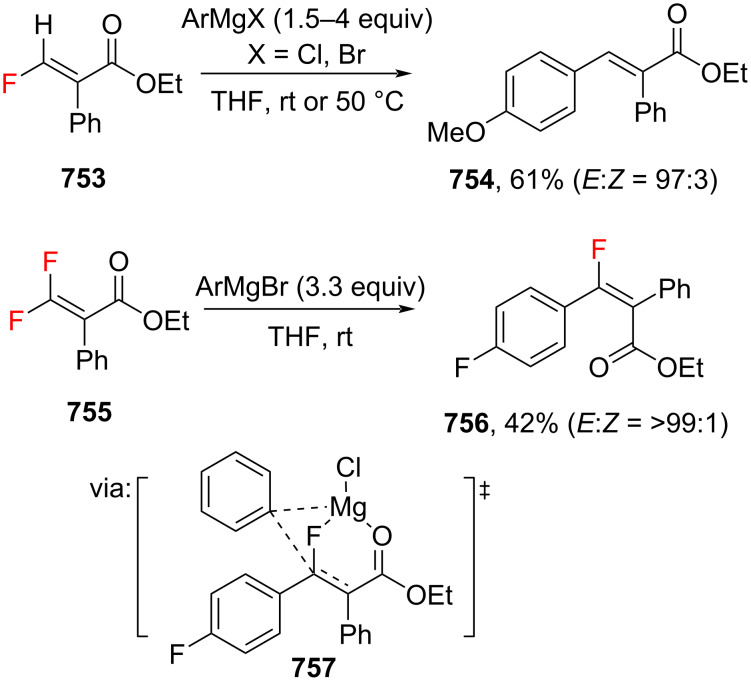
C(sp^2^)–F arylation using Grignard reagents.

Tsui and co-workers (2022) reacted Grignard reagents with monofluoroalkene **753** or *gem*-difluoroalkene **755** to give the corresponding β-arylated cinnamate esters **754** or **756**, respectively, via a concerted mechanism (**757**) (Scheme 134) [234].

**Scheme 135:**
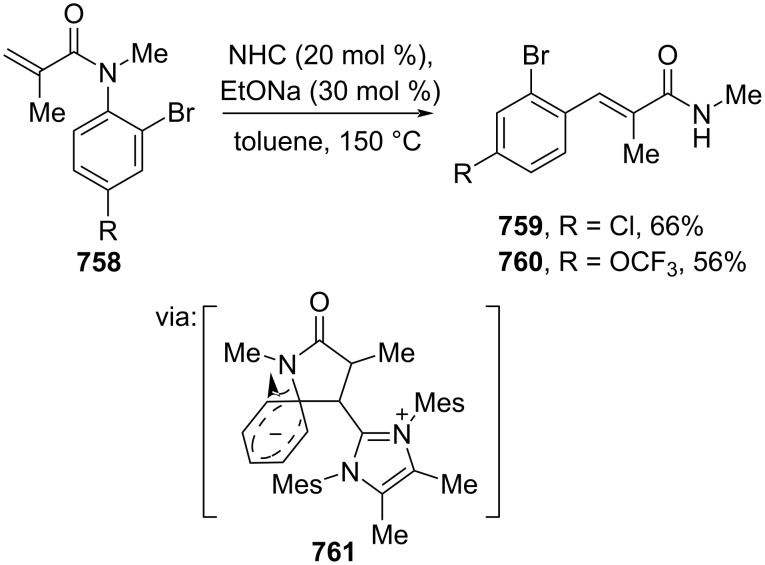
Truce–Smiles rearrangement of *N*-aryl metacrylamides.

Furthermore, Zhou and co-workers (2021) reported an NHC-mediated Truce–Smiles rearrangement of *N*-aryl metacrylamide **758** to prepare the corresponding (*E*)-cinnamamides **759** and **760** via lactam intermediate **761** (Scheme 135) [235].

**Scheme 136:**
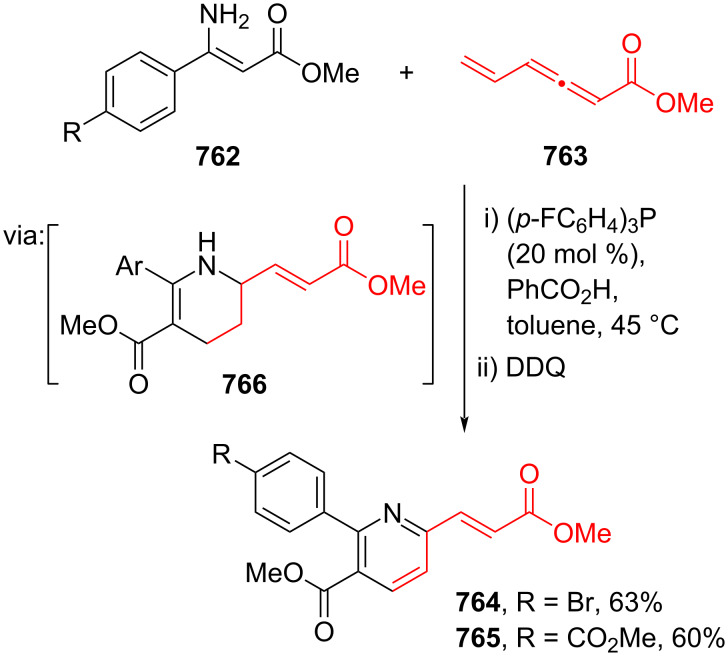
Phosphine-catalyzed cyclization of γ-vinyl allenoate with enamino esters.

In addition, Huang and co-workers (2022) investigated a one-pot two-step phosphine-catalyzed cyclization of γ-vinyl allenoate **763** with enamino ester **762** to give the corresponding 2,3,6-trisubstituted pyridines **764** and **765** after oxidative aromatization of intermediate **766** by DDQ (Scheme 136) [236]. The method has been successfully scaled up to a gram scale.

## Conclusion

Exploiting cinnamic acid building blocks for various purposes, especially for drug design, has led to more efficient, selective, and greener approaches in recent years. As shown in this review, plenty elegant one-step strategies have been developed to access not only common functional groups-anchored cinnamic acid derivatives, but also more exotic functional groups, thus potentially boosting hit-to-lead stages of drug discovery studies by providing new strategies with versatile functional groups. In addition, many methods shown here have been successfully carried out on a gram scale, potentially promoting scalable productions of bioactive cinnamic acid derivatives using these approaches.

However, the issue of strong dependency on rare transition metals in many areas should be addressed by increasing the exploration of novel strategies utilizing more sustainable sources, such as earth-abundant transition metals, and even metal-free transformations. In addition, designing recyclable catalysts (e.g., in cross-coupling reactions) is gaining considerable attention in the synthetic chemistry community. Similarly, tackling excessive reaction waste has been executed by designing recyclable coupling reagents. As cinnamic acid scaffolds are among prima donnas in drug design, there is no doubt that more advances will continue to emerge.

## Data Availability

Data sharing is not applicable as no new data was generated or analyzed in this study.
